# Identifying Traits Associated With Terminal Drought Tolerance in Sesame (*Sesamum indicum* L.) Genotypes

**DOI:** 10.3389/fpls.2021.739896

**Published:** 2021-12-10

**Authors:** Brij Bihari Pandey, P. Ratnakumar, B. Usha Kiran, Mangesh Y. Dudhe, G. Sowjanya Lakshmi, Kulasekaran Ramesh, Arti Guhey

**Affiliations:** ^1^Indian Council of Agriculture Research (ICAR)-Indian Institute of Oilseeds Research, Hyderabad, India; ^2^Department of Plant Physiology, Indira Gandhi Agricultural University, Raipur, India

**Keywords:** sesame, traits associations, genetic diversity, SSRs, terminal drought

## Abstract

Sesame is predominantly cultivated in rainfed and low fertile lands and is frequently exposed to terminal drought. *Sesamum* species inhabiting dryland ecosystems adaptively diverge from those inhabiting rainfed habitats, and drought-specific traits have a genetic basis. In sesame, traits associated with drought conditions have not been explored to date, yet studies of these traits are needed given that drought is predicted to become more frequent and severe in many parts of the world because of climate change. Here, 76 accessions from the available Indian core set were used to quantify variation in several traits under irrigated (WW) and terminal drought stress (WS) conditions as well as their association with seed yield over two consecutive years. The range of trait variation among the studied genotypes under WW and WS was significant. Furthermore, the traits associated with seed yield under WW and WS differed. The *per se* performance of the accessions indicated that the expression of most traits was reduced under WS. The correlation analysis revealed that the number of branches, leaf area (LA), leaves dry weight (LDW), number of capsules plant^–1^, and harvest index (HI) were positively correlated with seed yield under WW and WS, and total dry matter (TDM), plant stem weight, and canopy temperature (CT) were negatively correlated with seed yield under WW and WS, indicating that smaller and cooler canopy genotypes had higher yields. The genotypes IC-131936, IC-204045, IC-204861, IC-205363, IC-205311, and IC-73576 with the highest seed yields were characterized by low canopy temperature, high relative water content, and high harvest index under WS. Phenotypic and molecular diversity analysis was conducted on genotypes along with checks. Phenotypic diversity was assessed using multivariate analysis, whereas molecular diversity was estimated using simple sequence repeat (SSR) loci to facilitate the use of sesame in breeding and genetic mapping. SSRs showed low allelic variation, as indicated by a low average number of alleles (2.31) per locus, gene diversity (0.25), and polymorphism information content (0.22). Cluster analysis (CA) [neighbor-joining (NJ) tree] revealed three major genotypic groups and structure analysis showed 4 populations. The diverse genotypes identified with promising morpho-physiological traits can be used in breeding programs to develop new varieties.

## Introduction

Sesame is an ancient and important edible oilseed crop, but it has been neglected and underutilized compared with other crop species; consequently, there is still much room for its genetic improvement (Dossa et al., 2016; Ratnakumar and Ramesh, 2019). In India, the crop is predominantly cultivated under rainfed conditions in marginal soils. Therefore, productivity is affected by the vagaries of monsoon rains. The productivity of sesame in India is often low because of drought. An improved understanding of the traits associated with drought tolerance may open new avenues for sesame crop improvement.

Drought affects sesame productivity (Wang et al., 2016), growth, flower abortion, capsule development, and yield (Boureima et al., 2012; Dossa et al., 2017a,c). Several traits and genes involved in drought tolerance have been identified in oilseed crops, such as transpiration efficiency (Ratnakumar et al., 2009; Ratnakumar and Vadez, 2012; Vadez and Ratnakumar, 2016), quantitative trait loci (QTLs) in sesame (Dossa et al., 2019), and drought-responsive regulatory genes in canola (Bhardwaj et al., 2015) soybean (Chen et al., 2016), sunflower (Liang et al., 2017), and peanut (Bhogireddy et al., 2020). Most previous studies of drought in sesame were field or pot studies with few accessions (Shokoohfar and Yaghoubi, 2013; Golestani and Pakniyat, 2015; Boureima et al., 2016; Dossa et al., 2017b; Ravitej et al., 2019; Sravanthi et al., 2021). India has a large and diverse collection of sesame germplasm resources (Joshi et al., 1961; Nayar and Mehra, 1970; Simmonds (ed.), 1976). A core set developed by Bisht et al. (1998) of 314 genotypes represents approximately 3,000 sesame germplasm accessions.

The phenotypic and genetic variability of these Indian genotypes for drought adaptability has not yet been estimated using multivariate analysis techniques such as principal component analysis (PCA) and cluster analysis (CA). However, these techniques have been employed in other crops (Gharib-Eshghi et al., 2016; Iqbal et al., 2018; Saljooghianpour and Javadzadeh, 2018) to establish relationships among genotypes, identify superior genotypes (Tabrizi et al., 2011; Dudhe et al., 2018), and explore genotypic diversity (Dudhe et al., 2019). CA has also been used to cluster accessions with similar characteristics into homogenous categories in multidimensional space.

DNA markers coupled with phenotypic diversity provide a more complete understanding of genotypes. In sesame, multi-locus DNA markers such as amplified fragment length polymorphism (AFLP) (Laurentin and Karlovsky, 2006; Ali et al., 2007; Laurentin et al., 2008), random amplified polymorphic DNA (RAPD) (Bhat et al., 1999; Ercan et al., 2004; Abdellatef et al., 2008; Pham et al., 2009; Tabatabaei et al., 2011) inter-SSR (Kim et al., 2008; Kumar and Sharma, 2011; Parsaeian et al., 2011; Kumar et al., 2012; Nyongesa et al., 2013), and sequence-related amplified polymorphism (Peng et al., 2010; Zhang et al., 2010, 2012) markers have been widely used to characterize genetic diversity and species relationships. SSR markers are ideal genetic markers for the assessment of genetic diversity in germplasm because of their availability in large numbers, genome-wide distribution, locus specificity, reproducibility, and co-dominant, multi-allelic, selectively neutral, and highly polymorphic properties. Significant effort has been made to develop SSR markers (Badri et al., 2014; Wei et al., 2014) and utilize them for the characterization of sesame germplasm (Pandey et al., 2015; Anandan et al., 2018).

Here, the traits involved in terminal drought stress (WS) of different sesame genotypes and their association with seed yield plant^–1^ (SWP) were identified. The objectives of this study were to: (i) identify the best-performing accessions under irrigated (WW) and WS, (ii) characterize the association of traits with SWP, (iii) estimate the phenotypic diversity among the studied genotypes, and (iv) characterize the molecular diversity among genotypes under WS.

## Materials and Methods

### Plant Materials

A set of 76 promising sesame (*Sesamum indicum* L.) genotypes from the sesame core set collection (developed by Bisht et al., 1998) were used as experimental materials, which included two national checks (i.e., GT-10 and TKG-22; better seed yielders under irrigated conditions). The selected accessions were mostly indigenous landraces adapted to different agro-ecological zones in India and from the same gene pool species: *S. indicum* L. (Supplementary Table 1).

### Experimental Conditions

The experiments were conducted during the late *Rabi* season of 2018 and 2019 at ICAR-IIOR Research Farm, Narkhoda, Hyderabad, India (17°15′16″ N, 78°18′30″ E; 542 m above sea level). The weather parameters of both years are presented in Supplementary Figure 1.

### Crop Management and Drought Treatment

The experiments were carried out using a strip-plot design. Each accession was sown in 4 m^2^ plots with a spacing of 45 cm (between rows) × 15 cm (between plants); there were three replicates for each treatment. Sowing was done by dibbling, and the recommended fertilizer dose (40 kg N + 20 kg P_2_O_5_ + 20 kg K_2_O/ha) was applied; other standard practices and need-based plant protection measures were followed to ensure a healthy crop. The soil was classified as sandy loam, which is poor in nutrients (C: 0.4%; N: 235 kg ha^–1^) and water-holding capacity (18%). The crop was initially irrigated twice after sowing. At the flowering stage (40–45 days after sowing), irrigation was withheld in WS plots. Soil moisture levels were monitored on an hourly basis with field-installed soil sensors (Manufactures: Proximal; Brand: SoilSenS, Model: V1.0). In WW plots, soil moisture was maintained up to 80% field capacity with need-based irrigation. The moisture levels in WS plots were decreased to 35–40% soil moisture, which was equivalent to −4.5 to −5 bars of soil water potential, and maintained until crop physiological maturity (90–95 days after sowing). Eight soil sensors were installed at 45 cm of soil depth for moisture measurements in WW and WS plots. These soil sensors were powered with a solar compartment and provided online data of soil moisture (in %), ambient temperature, and humidity at 6 h intervals. Irrigation was scheduled for WW plots based on the data provided by the sensor systems. WS plots were not irrigated from the flowering stage to physiological maturity. To determine the soil metric potential, the soil moisture values in WW and WS were plotted against the values derived from the soil surface tensiometer. The soil metric potential ranged from −0.55 to −0.75 bars in WW plots and from −4.5 to −5 bars in WS plots. Graphical representations of the soil metric potential in WW and WS are presented in Supplementary Figure 2.

### Morpho-Physiological and Yield Traits

Data on morphological parameters such as number of days to flower (NDF), number of days to maturity (NDM), plant height (PH), number of branches (NBP), leaf area (LA), leaves dry weight (LDW), total dry matter (TDM), and plant stem weight (PST) were recorded from 10 randomly selected plants of each genotype in the three replications of each treatment. The physiological traits, namely chlorophyll meter readings (SPAD), canopy temperature (CT), and relative water content (RWC), were recorded after 30 days of stress exposure using SPAD-502 Plus (Konica Minolta, Inc.) and an IR thermal gun (AGRI-THERM-6210L; Everest Inter-science Inc.). The measurements were made on sunny days between 10:00 and 13:00 h. RWC content was determined using fresh leaves collected after CT measurements. The yield traits SWP, number of capsules plant^–1^ (NCP), test weight (TW), capsules weight plant^–1^ (CWP), and harvest index (HI) were recorded at crop maturity.

### Simple Sequence Repeat Analysis

DNA was extracted from the pooled leaf samples (10 plants per genotype) using the protocol developed by Doyle and Doyle (1987) with slight modifications. Specifically, 1 g of leaf tissue was grind to a fine powder in liquid nitrogen, followed by incubation in 5 ml of pre-heated extraction buffer (3% w/v CTAB, 1.4 M NaCl, 100 mM Tris–HCl (pH 8), 20 mM EDTA, 2% w/v PVP, and 0.2% v/v ß-mercaptoethanol) for 1 h at 65°C. The homogenate was extracted with chloroform:isoamyl alcohol (24:1) and centrifuged at 6,000 × *g* at 25°C for 15 min; DNA was precipitated with equal volumes of isopropanol, washed once with 70% ethanol, air-dried, and dissolved in TE buffer (10 mM Tris–HCl (pH 8), 1 mM EDTA). DNA was then treated with bovine pancreatic RNase, extracted once with phenol:chloroform (1:1), and twice with chloroform:isoamyl alcohol (24:1). After precipitation with absolute ethanol, the precipitate was washed with wash buffer I (absolute ethanol and 3 M sodium acetate) and wash buffer II (absolute ethanol and 1 M ammonium acetate). The pellet was air-dried, suspended in an appropriate volume of T_10_E_0_._1_ buffer, and quantified in a spectrophotometer.

About 75 SSR primer pairs were used to estimate molecular diversity in sesame genotypes published by Wei et al. (2014). The details of the SSR primer pairs are provided in Supplementary Table 21. The PCR was carried out with the following conditions: 94°C for 5 min (initial denaturation), 35 cycles of 94°C for 30 s (denaturation), 55°C for 30 s (annealing), 72°C for 30 s (extension), and 72°C for 7 min (final extension) in a Master Cycler Gradient Eppendorf version 2.1 (Eppendorf, United States). Different annealing temperatures were used depending on the requirements for a specific primer pair. The PCR products were resolved using 3.5% agarose gel electrophoresis, and the polymorphisms were visualized in the gel documentation system. The polymorphic SSR alleles were scored as co-dominant markers using different characters (e.g., 1, 2, 3).

### Statistical Analysis

The data were summarized using descriptive statistics and analyzed using correlation analysis, CA, and PCA. Analysis of variance (ANOVA) was conducted for each trait under WW and WS conditions as described by Panse and Sukhatme (1964). Phenotypic correlations were determined following Johnson et al. (1955). CA was used to cluster the sesame genotypes based on their quantitative traits. PCA was used to characterize trait variation. R (version 3.1.3) was used for PCA and CA (R Core Team, 2013), and the command “hclust” was used to draw a dendrogram. The biplot was generated using “FactoMineR” (Factor analysis and data processing with R) package (Husson et al., 2009). Path coefficient analysis was carried out to indicate the direct and indirect association between different traits under both WW and WS conditions. The software R (version 3.1.3) was used for correlation co-efficient and path coefficient analysis (R Core Team, 2013). Several molecular genetic diversity estimates—the number of alleles (NA), gene diversity (expected heterozygosity; He), and the polymorphic information content (PIC)—were obtained using Power marker version 3.25 (Liu and Muse, 2005). A neighbor-joining (NJ) tree was constructed based on simple matching coefficients as implemented in Dissimilarity Analysis and Representation for Windows (DARwin) V.5.0.158 (Perrier and Jacquemoud-Collet, 2006) to depict the genetic diversity among genotypes. Genetic structure among the sesamum genotypes was studied based on a model-based clustering using STRUCTURE v.2.3.4 (Pritchard et al., 2000) to infer the population structure using a brn-in of 50,000, a run length of 100,000 and a model allowing for a admixture an correlated allele frequencies. The delta K measure (Evanno et al., 2005) was used to determine the K as implemented in the online version of STRUCTURE HARVESTER^1^ (Earl and VonHoldt, 2012).

## Results

### Mean Performance of Sesame Genotypes Under Well-Watered and WS Conditions

The sesame genotypes showed notable variation among most of the traits under WW as well as WS conditions (Supplementary Tables 2, 3). ANOVA indicated that there were significant differences among treatments (A), accessions (B), their interaction (A × B), and replicates (Tables 1, 2). There was variation among genotypes in NDF across years and treatments. NDM ranged from 88 to 110 days with an average of 95.97 days under WW condition and from 86 to 106 days with an average 93.39 under WS conditions during both the years. The PH was reduced under WS in all sesame genotypes, the observed average reduction in PH was 18.07%; although the reduction under WS was less pronounced in 2019. There was high variation in NBP among genotypes; it ranged from 3 to 7 (WW) and from 2 to 6 (WS). The development of optimal LA is important for dry matter accumulation. In both years, LA varied from 300.23 to 1248.31 cm^2^ (WW) and from 255.45 to 799.18 cm^2^ (WS). A drastic reduction under WS was also noted for LDW, which ranged from 3 to 7 g across both years. Traits such as TDM and PST showed large variation under both WW and WS. The maximum TDM ranged from 29 to 32 g (WW) and was 27 g (WS). PST ranged from 6.74 to 16.96 g (WW) and from 2.22 to 12.35 g (WS) during the first year but ranged from 7.67 to 17.66 g (WW) and from 2.13 to 16.73 g (WS) during the second year.

**TABLE 1:** Analysis of variance (ANOVA) of 76 sesame genotypes along with checks during the year 2018.

		NDF		NDM		PH		NBP		LA		LDW		TDM		PST	
Source	DF	MSS	*F* value	MSS	*F* value	MSS	*F* value	MSS	*F* value	MSS	*F* value	MSS	*F* value	MSS	*F* value	MSS	*F* value
Replication	2	210.6	0.0910 NS	56.0	0.0201*	67.9	0.0278*	16.5	0.0309*	40.0	0.0299*	202.50	0.0167*	317.1	0.0217*	74.9	0.182*
Treatments (A)	1	13182.5	0.0821 NS	47.1	0.0036*	842.3	0.0160*	13.2	0.0283*	51.7	0.0303*	13096.1	0.0021*	38564.0	0.0084*	456.8	0.0442*
Genotype (B)	75	148.6	<0.0003*	1.52	0.0007*	8.26	<0.0001*	0.63	<0.0001*	1.69	0.0006*	139.53	<0.0001*	471.33	<0.0001*	3.66	<0.0001*
A × B	75	177.9	<0.0004*	1.89	<0.0003*	8.99	<0.0001*	0.13	<0.0001*	1.73	<0.0001*	198.91	<0.0001*	859.02	<0.0001*	2.86	<0.0001*
CV (%) (A)		1.22		17.26		7.24		5.43		16.73		0.55		3.19		4.38	
CV (%) (B)		1.83		15.36		9.09		10.46		18.37		2.16		2.29		5.71	
CV (%) (A × B)		0.98		10.83		3.22		1.52		12.56		0.72		0.88		4.58	
SE (A)		0.06		0.07		0.07		0.02		0.09		0.04		0.31		0.18	
SE (B)		0.98		0.58		0.54		0.31		0.62		1.04		1.31		1.22	
SE (A × B)		0.52		0.61		0.29		0.04		0.60		0.49		0.77		0.77	
SE (B × A)		1.17		0.98		0.52		0.27		0.75		1.10		1.41		1.41	

		SPAD		CT		RWC		SWP		NCP		TW		CWP		HI	
Source	DF	MSS	*F* value	MSS	*F* value	MSS	*F* value	MSS	*F* value	MSS	*F* value	MSS	*F* value	MSS	*F* value	MSS	*F* value

Replication	2	76.7	0.0381*	15418.34	0.111*	69.5	0.0278*	200.8	0.0280*	57.4	0.367*	248.5	0.0073*	0.96	0.129*	89.58	0.120*
Treatments (A)	1	1580.3	0.0084*	3138395.4	0.0079*	804.1	0.0170*	566.4	0.0167*	422.1	0.228*	8494.6	0.0012*	502.98	0.154*	1021.97	0.0117*
Genotype (B)	75	21.9	<0.0001*	54444.3	<0.0001*	9.4	<0.0001*	224.2	0.3405*	82.8	<0.0001*	149.7	<0.0001*	4.58	<0.0001*	3.00	<0.0001*
A × B	75	22.6	<0.0001*	55244.2	<0.0001*	10.6	<0.0001*	156.1	<0.0001*	111.1	<0.0001*	219.2	<0.0001*	5.33	<0.0001*	5.03	<0.0001*
CV (%) (A)		2.80		3.73		6.01		3.98		8.26		1.25		5.34		8.08	
CV (%) (B)		5.06		2.46		7.01		5.40		6.27		2.72		7.82		8.50	
CV (%) (A × B)		1.77		1.14		4.33		1.91		3.19		1.53		2.41		3.69	
SE (A)		0.06		0.27		0.09		0.07		0.04		0.02		0.84		0.37	
SE (B)		0.67		1.21		0.66		1.20		0.26		0.39		0.34		0.68	
SE (A × B)		0.34		0.42		0.37		0.29		0.83		0.38		0.96		0.52	
SE (B × A)		0.71		1.25		0.71		0.82		0.26		1.42		0.47		0.73	

The meaning of symbol “*” is significant at 5% level.

**TABLE 2:** Analysis of variance (ANOVA) of 76 sesame genotypes along with checks during the year 2019.

			NDF		NDM		PH		NBP		LA		LDW		TDM		PST	
Source	DF	MSS	*F* value	MSS	*F* value	MSS	*F* value	MSS	*F* value	MSS	*F* value	MSS	*F* value	MSS	*F* value	MSS	*F* value
Replication	2	202.50	0.0799 NS	40.00	0.0167*	65.89	0.0221*	12.89	0.0283*	74.96	0.382*	317.1	0.0317*	15418.3	0.151*	76.79	0.0381*
Treatments (A)	1	13096.14	0.0703 NS	51.79	0.0021*	768.21	0.0148*	10.78	0.0301*	456.87	0.0442*	38564.0	0.0084*	3138395.4	0.0079*	1580.35	0.0084*
Genotype (B)	75	139.53	<0.0001*	1.69	0.0006*	7.19	<0.0001*	0.58	<0.0001*	3.66	<0.0001*	471.3	<0.0001*	54444.3	<0.0001*	21.95	<0.0001*
A × B	75	198.91	<0.0001*	1.73	<0.0001*	6.83	<0.0001*	0.17	<0.0001*	2.86	<0.0001*	859.0	<0.0001*	55244.2	<0.0001*	22.65	<0.0001*
CV (%) (A)		0.55		16.73		6.18		6.83		8.38		0.55		3.73		2.80	
CV (%) (B)		2.16		18.37		7.53		8.49		7.71		2.16		2.46		5.06	
CV (%) (A × B)		0.72		12.56		4.02		2.19		0.58		0.72		1.14		1.77	
SE (A)		0.04		0.09		0.06		0.04		0.18		0.04		2.60		0.06	
SE (B)		1.04		0.62		0.47		0.35		0.68		1.04		10.21		0.67	
SE (A × B)		0.49		0.60		0.27		0.09		0.33		0.49		7.12		0.34	
SE (B × A)		1.10		0.75		0.43		0.54		0.71		1.10		11.25		0.71	

		SPAD		CT		RWC		SWP		NCP		TW		CWP		HI	
Source	DF	MSS	*F* value	MSS	*F* value	MSS	*F* value	MSS	*F* value	MSS	*F* value	MSS	*F* value	MSS	*F* value	MSS	*F* value

Replication	2	200.88	0.0280*	69.56	0.0378*	112.56	0.0247*	248.50	0.0073*	48.61	0.203*	85609.11	0.358*	0.96	0.129*	89.58	0.120*
Treatments (A)	1	566.40	0.0167*	804.15	0.0170*	2845.97	0.0129*	8494.69	0.0012*	367.48	0.189*	4074297.54	0.0200*	502.98	0.154*	1021.97	0.0117*
Genotype (B)	75	1.10	<0.0001*	9.42	<0.0001*	51.40	<0.0001*	149.76	<0.0001*	79.58	<0.0001*	37108.64	<0.0001*	4.58	<0.0001*	3.00	<0.0001*
A × B	75	27.21	<0.0001*	10.60	<0.0001*	32.67	<0.0001*	219.20	<0.0001*	111.19	<0.0001*	43205.86	<0.0001*	5.33	<0.0001*	5.03	<0.0001*
CV (%) (A)		1.98		9.01		2.10		0.25		5.58		6.43		5.34		8.08	
CV (%) (B)		5.40		8.01		2.29		2.72		4.61		5.59		7.82		8.50	
CV (%) (A × B)		0.91		1.33		0.77		0.53		2.63		0.96		2.41		3.69	
SE (A)		0.07		0.09		0.13		0.02		0.05		7.52		0.84		0.37	
SE (B)		1.20		0.66		0.84		1.39		0.74		1.91		0.34		0.68	
SE (A × B)		0.29		0.37		0.42		0.38		0.89		0.34		0.96		0.52	
SE (B × A)		1.22		0.71		0.88		1.42		0.91		0.92		0.47		0.73	

The meaning of symbol “*” is significant at 5% level.

The chlorophyll content is important for plants to harness light energy for dry matter synthesis. SPAD values were higher under WS and ranged from 43 to 65 (WS) during both years and under WW condition from 31 to 58 with an average of 48.1. The SPAD values showed significant correlation with CT values under WW and WS condition for 2018 and 2019 years, respectively. CT values during both years ranged from 27.0 to 32.2°C (WW) and from 30.1 to 35.3°C (WS). RWC values were reduced under WS and ranged from 40.8 to 83.0% (WS) and from 59.5 to 91.6% (WW) (Supplementary Tables 2, 3). Leaf RWC is an important trait for drought adaptation.

A significant variation was observed among the sesame genotypes in terms of yield parameters, including total seed weight and HI. NCP ranged from 43 to 149 (WW) and from 43 to 101 (WS). A significant reduction was observed in TW (1,000-seed weight) under WW as well as WS conditions, it ranged from 1.45 to 4.50 g (WW) and from 2.07 to 4.21 g (WS) in 2018. In year 2019, TW ranged from 1.91 to 4.94 g under WW condition and from 2.20 to 4.26 g under WS condition. The seed weight per plant (SWP) also reduced under WS condition. It ranged from 3 to 12 g (WW) and from 2 to 8 g (WS) across both the years (Supplementary Tables 2, 3). High variation was observed in CWP in both years. The HI ranged from 12 to 43% (WW) and from 7 to 46% (WS) in 2018; whereas from 9 to 45% (WW) and from 13 to 39% (WS) in 2019.

Based on the *per se* performance, promising genotypes were identified for maximum PH (IC-41920), higher NBP (IC-204861), LA (IC-73576), SPAD (IC-131546), RWC (IC-43036), NCP (IC-204753), SWP (IC-73576),TW (IC-205285), and lower CT (IC-132558) under WW. Under WS, promising genotypes were identified for maximum PH (IC-204666), NBP, RWC, and SWP (IC-205311); LA (IC-204046); SPAD (IC-23279); NCP (IC-204300); TW (IC-16244); and lower CT (IC-204861) compared with the checks.

### Association of Traits With Seed Yield

The oil quantity and quality of sesame depends on the SWP and TW. The traits associated with SWP can be used to aid the selection of superior genotypes. The correlation analysis revealed that in both years under WS, the traits NBP, LA, LDW, RWC, NCP, and HI were positively correlated with SWP, and TDM, PST, and CT were negatively correlated with SWP. The same positive correlations were observed under WW (Supplementary Tables 4, 5). The traits NBP, LA, LDW, TDM, PST, and HI were highly correlated with SWP under WW and WS during both years. CT was negatively correlated with SWP, indicating that cooler CTs ensure higher yields of genotypes under WS. Furthermore, a negative correlation between TDM and PST under WS indicates that the translocation of photosynthates was reduced. We found that HI, the measurement of economic yield was positively correlated with NBP and SWP, whereas it negatively correlated with NDF, NDM, TDM, PST, and CT under WS condition in both the years. Under WW condition, the HI positively correlated with NBP, LA, RWC, SWP, and NCP; and negatively correlated with TDM, PST, CT, TW, and CWP in both the years.

### Path Analysis

The correlation studies were carried out to find the correlation co-efficient of different morpho-physiological traits to seed yield at both genetic and phenotypic levels under different water regimes for both the years (Supplementary Tables 6–13; Dewey and Lu, 1959). The characteristics such as NBP, LA, LDW, NCP, and HI showed the positive correlation with seed yield; whereas TDM and PST were found to be negatively correlated with seed yield at genotypic as well as phenotypic levels under WW conditions during both the years. Correspondingly, during first year the traits such as NBP and HI correlated positively under WS conditions; while, TDM and CT correlated negatively at genotypic and phenotypic levels during both the years. However, it was observed that during second year the traits such as CT negatively correlated at genotypic levels only; while TDM were found negatively correlating with seed yield at phenotypic levels only; indicating that CT is a genetically determined trait; whereas, TDM is influenced by the environment conditions and highly depend on the growing conditions.

As the correlation studies can only provide one way information regarding the effect of a particular trait on yield, therefore, to partition direct and indirect effects of each trait on seed yield path co-efficient analysis was done separately for both the years and conditions (WW and WS). The path analysis revealed that under WW conditions the traits such as HI, TDM, LDW, LA, and NDM had a direct and positive effect on seed yield at both genotypic and phenotypic levels. In contrast, under WS conditions traits like TDM, HI, and RWC had highest and positive direct effect on seed yield at both genotypic and phenotypic levels. In addition, trait HI with NBP, LA and LDW under WW conditions; whereas TDM with NCP, HI with NBP and LA under WS conditions had an indirect effect on seed yield at both genotypic and phenotypic levels.

### Principal Component Analysis

**FIGURE 1:**
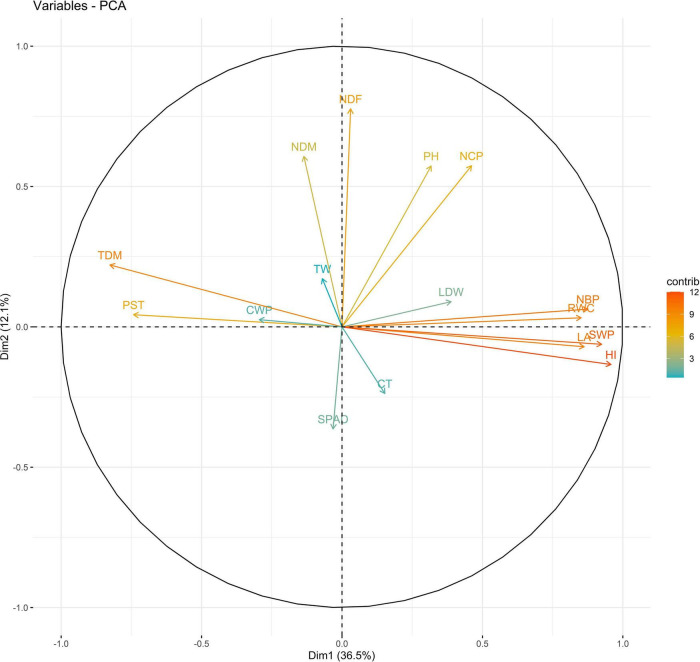
Biplot between PC1 and PC2 showing contribution of various traits during year 2018 season under well-watered (WW) conditions.

**FIGURE 2:**
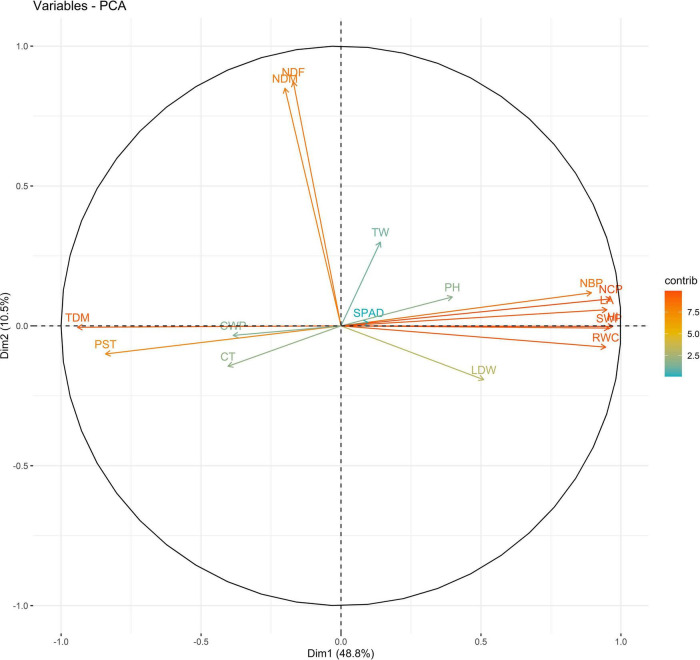
Biplot between PC1 and PC2 showing contribution of various traits during year 2018 season under WS conditions.

Principal components (PCs) with eigen values greater than 1 explained more than 70% of the variation in the data for the PCA of traits under WW and WS. In 2018, the first five PCs explained 72.2% of the variation in the data under WW; the first four PCs explained 75.5% of the variation in the data under WS (Supplementary Table 14). PC1 and PC2 explained 36 and 12% of the variation in the data under WW and 48 and 10% of the variation in the data under WS, respectively. Under WS, HI, NCP, and SWP loaded positively on PC1, and NDF loaded negatively on PC1. NDF and NDM loaded positively on PC2, and HI loaded negatively on PC2 under WW (Figures 1, 2 and Supplementary Table 15).

**FIGURE 3:**
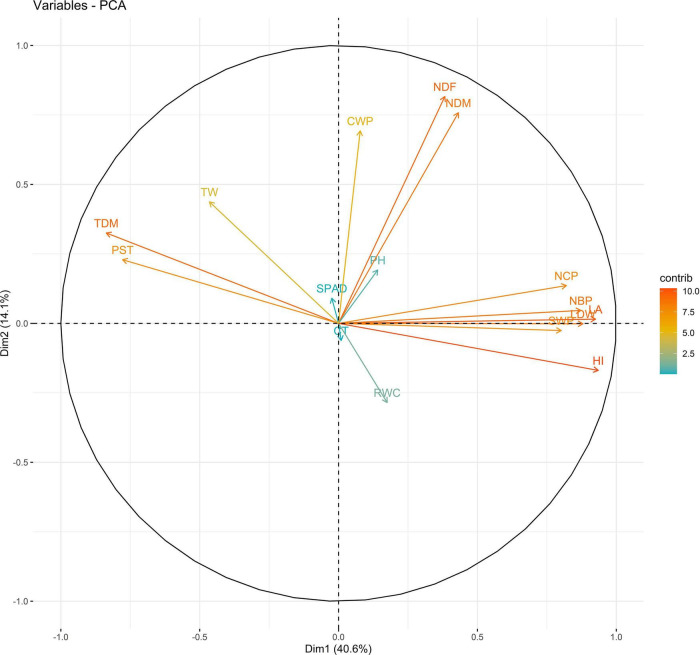
Biplot between PC1 and PC2 showing contribution of various traits during year 2019 season under well-watered (WW) conditions.

**FIGURE 4:**
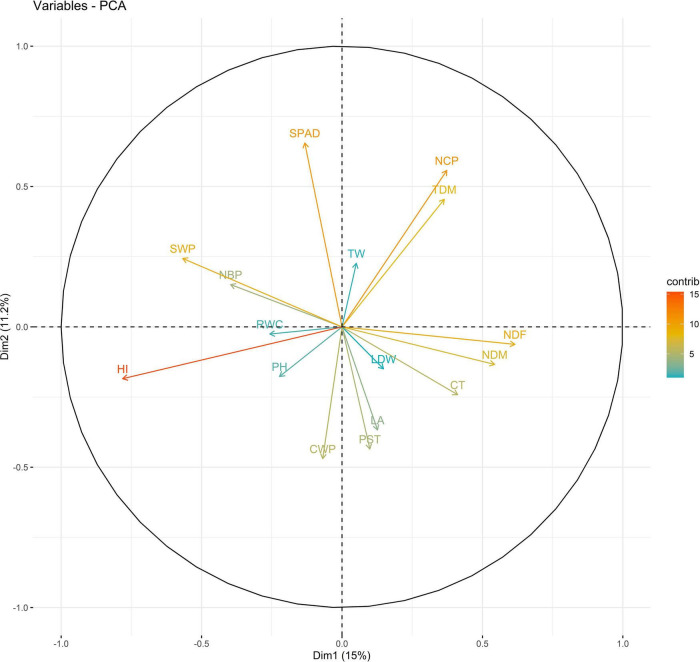
Biplot between PC1 and PC2 showing contribution of various traits during year 2019 season under WS conditions.

In 2019, PC1 to PC5, all of which had eigen values greater than 1, explained more than 75% to the variation in the data under WW. PC1 and PC2 explained the most variation in the data. Under WS, PC1 to PC7, all of which had eigen values greater than 1, explained 68% of the variation in the data. PC1 and PC2 explained 14 and 11% of the variation in the data, respectively (Supplementary Table 14). Under WW, HI and LA loaded positively on PC1, and SPAD loaded negatively on PC1; NDM and NDF loaded positively on PC2. Under WS, NDF, and NDM loaded positively on PC1; NCP and SPAD loaded positively on PC2, and RWC loaded negatively on PC2 (Figures 3, 4 and Supplementary Table 15).

**FIGURE 5:**
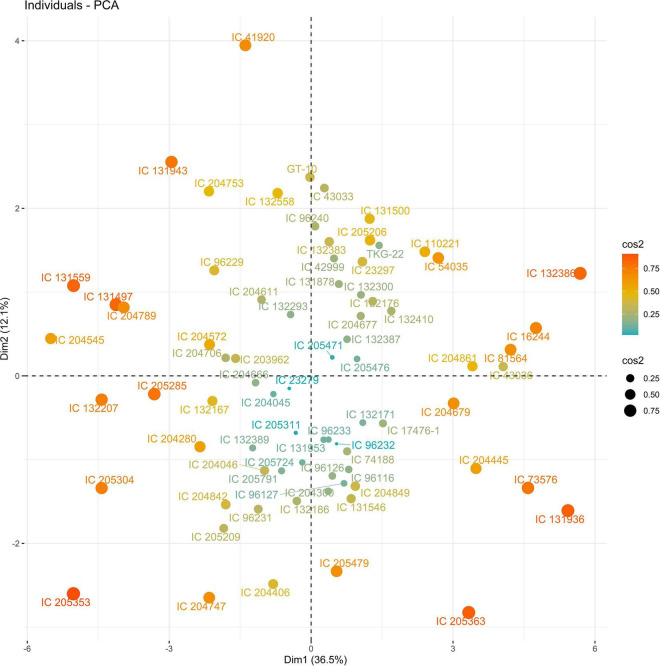
Principal scatter plot of the first two principal component analysis (PCA) of 76 sesame genotypes during year 2018 under well-watered (WW) conditions.

**FIGURE 6:**
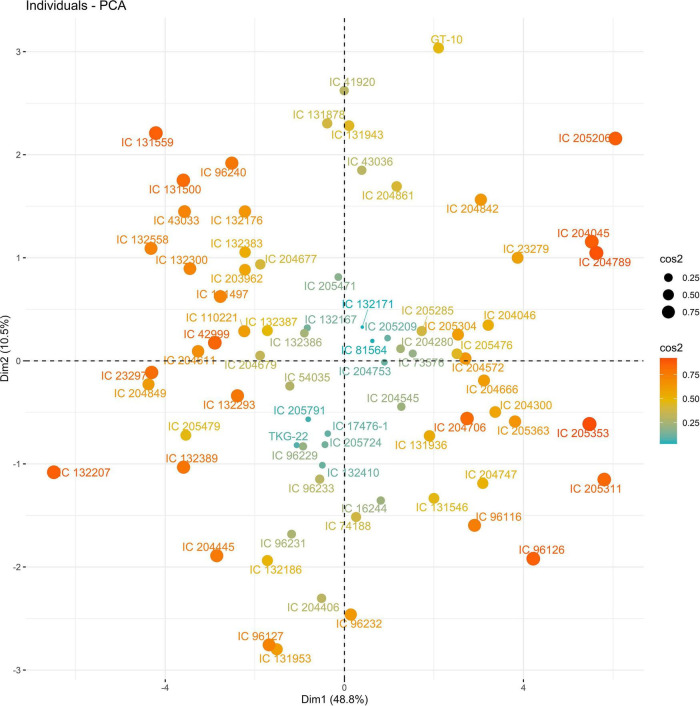
Principal scatter plot of the first two principal component analysis (PCA) of 76 sesame genotypes during year 2018 under WS conditions.

**FIGURE 7:**
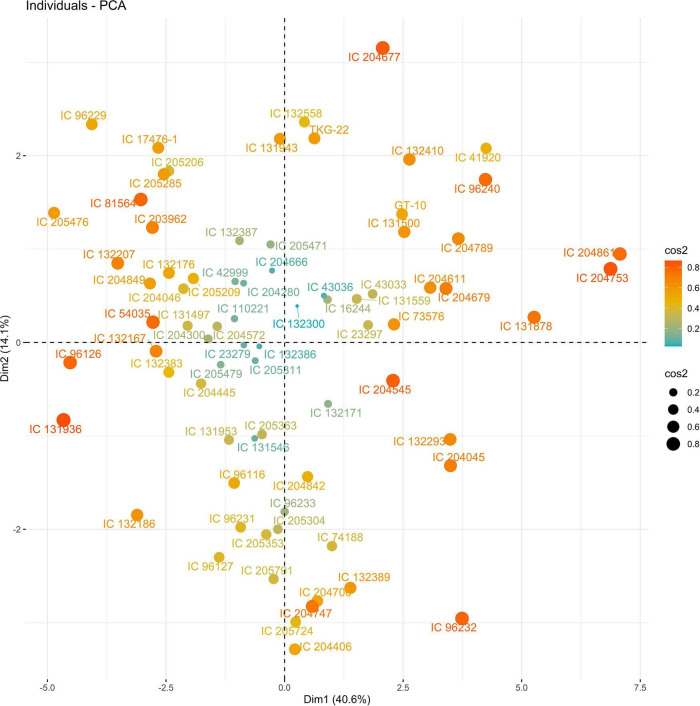
Principal scatter plot of the first two principal component analysis (PCA) of 76 sesame genotypes during year 2019 under well-watered (WW) conditions.

**FIGURE 8:**
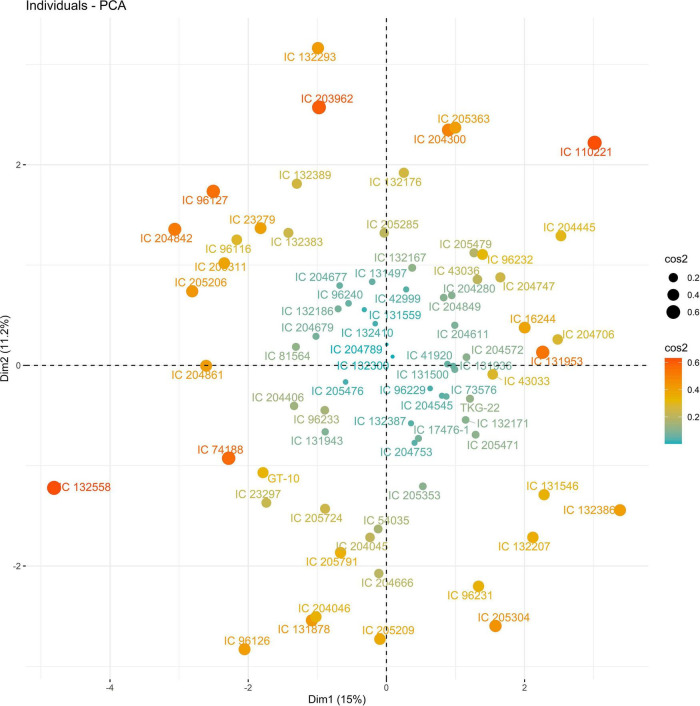
Principal scatter plot of the first two principal component analysis (PCA) of 76 sesame genotypes during year 2019 under WS conditions.

The PC biplot under WW shows the variables on the plot as vectors. The biplot shows that the traits NDF, NDM, LA, SPAD, NCP, SWP, and HI contributed the most to variation among genotypes under WW and WS. Furthermore, the principal scatter plot suggested that the accessions IC-132171, IC-204666, IC-205209, IC- 205311, IC-205471, and IC-23279 under WW and IC-132410, IC-204545, IC-204753, IC-205209, IC-205471, IC-73576, IC-17476-1, and IC-81564 under WS were positioned near the axis, suggesting that these genotypes performed similarly and show the least amount of variation tested (Figures 5–8).

### Cluster Analysis

A large variation was observed among the sesame genotypes between water regimes when the data were subjected to hierarchical clustering (grouped into 10 clusters). During the first year under WW, cluster IV consisted of 19 accessions, whereas cluster VIII and cluster X had only one accession each, suggesting that these accessions were distinct. Under WS, cluster III consisted of 15 accessions, followed by cluster II with 14 accessions (Supplementary Table 16). Based on the cluster mean values, the genotypes belonging to clusters V and VII had the highest SWP under WW, whereas genotypes belonging to cluster IV and cluster V had the highest SWP under WS. These genotypes were superior in other secondary traits, such as LA, RWC, NCP, and HI (Supplementary Table 18). The CA for the second year revealed that cluster IV (15 genotypes) and cluster II (11 genotypes) had the highest SWP under WW. In addition, the genotypes of these two clusters were late maturing and had the highest values for LA, NBP, NCP, and RWC. Under WS, cluster I had 19 genotypes including the checks, suggesting that the performance of cluster I genotypes and the checks was similar (Supplementary Table 17). Based on the mean values, clusters VI and X were the most promising. The genotypes belonging to these clusters had the highest SWP, HI, and SPAD (Supplementary Table 19).

Based on the SWP and cluster means under WW, genotypes IC-204445, IC-205209, IC-205304, IC-205471, and IC-205479 were the most promising accessions; under WS, genotypes IC-131936 and IC-205209 were the most promising accessions. IC-205209 was the most promising genotype under both WW and WS and during both years. The secondary traits NCP and LA might be genetically determined given that these traits had a significant effect on SWP under both WS and WW.

### Genetic Diversity

**FIGURE 9:**
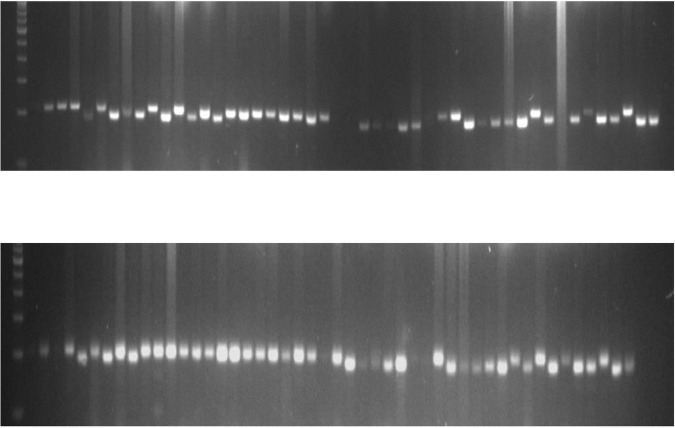
Gel picture showing the allelic variation of SSR locus sesamum EST marker (SEM) 53 and SEM 4 across sesame genotypes.

A total of 75 polymorphic markers previously reported by Wei et al. (2014) were used to evaluate the diversity of sesame landraces in India. Out of 75 SSR primer pairs, 48 (65%) were polymorphic in the genotypes studied. SSR primer pairs produced a total of 111 alleles across 76 sesame genotypes. The number of SSR alleles ranged from 2 to 4 with an average of 2.31 per locus. Most primer pairs (36) produced only two alleles. The major allele frequency ranged from 0.43 to 1.00 with an average of 0.84. There were four rare alleles detected. Observed heterozygosity ranged from 0.000 to 0.035 with an average of 0.003. He ranged from 0.01 to 0.51 with an average of 0.25. The PIC values of SSR primer pairs ranged from 0.03 to 0.46 with an average of 0.22. Details of the loci are provided in Supplementary Table 20. The gel profile showing allelic variation of the SSR loci SIM 19 and SIM 53 and across sesame genotypes was shown in Figure 9, which may be more useful for diagnostic applications in sesame.

**FIGURE 10:**
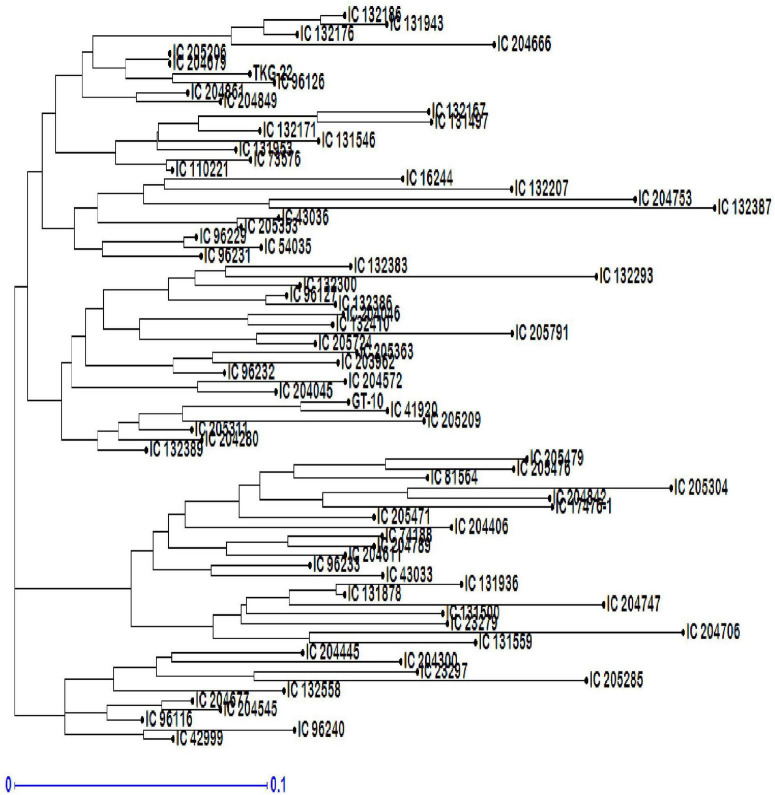
Neighbor-joining (NJ) tree-based dendrogram depicting genetic diversity among 76 genotypes of sesame.

The NJ tree showed three major genotypic clusters within the collection of 76 genotypes (Figure 10). Cluster 1 was a major group (G1) including 46 genotypes with several subgroups, cluster 2 (G2) included 20 genotypes, and cluster 3 consisted of 10 genotypes. Overall, pair-wise simple matching coefficients ranged from 0.01 (IC 204679–IC 205206) to 0.60 (IC 132387–IC 204747) with an average of 0.250. The pair-wise simple matching coefficients of G1 ranged from 0.01 (IC 204679–IC 205206) to 0.589 (IC 204706–IC 205285), G2 ranged from 0.053 (IC 13878–IC 131936) to 0.607 (IC 132387–IC 204747), and G3 ranged from 0.035 (IC 132389–IC 205311) to 0.446 (IC 131559–IC 23297).

### Population Structure

**FIGURE 11:**
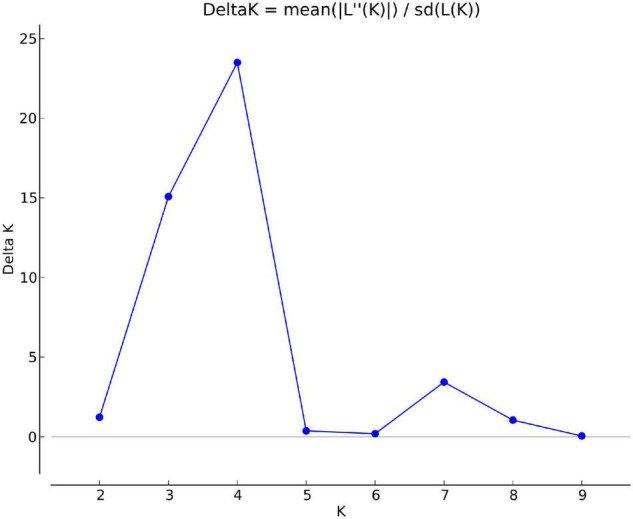
Delta *K* values for different numbers of populations assumed (K) in the STRUCTURE analysis.

**FIGURE 12:**
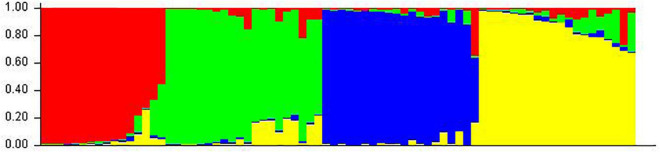
Classification of 76 sesame accessions into four populations (*K* = 4) using STRUCTURE v2.3.4. Each accession is represented by a single row, which is partitioned into colored segments (P1 = Red, P2 = Green, P3 = Blue, and P4 = Yellow) in proportion to the estimated membership in the four subpopulations.

Structure analysis was performed by setting a possible number of clusters (K) from 1 to 10 with 10 replications for each K. The LnP(D) value for each given K increased with the increase in K, but as there was no sudden change in LnP(D), the probable *K* value could not be inferred. However, delta-K (DK) analysis of LnP(D) (Evanno et al., 2005), showed a sharp peak at *K* = 4, suggesting four populations within the collection of 76 sesamum genotypes (Figure 11). Based on the threshold value of the membership coefficient (≥0.75), 69 genotypes were assigned to four populations (namely, P1, P2, P3, and P4) and 7 genotypes to the admixture group. The bar plot showing the population structure for *K* = 4 was shown in Figure 12. P1 comprised of 14 (18.4%), P2 comprised of 18 (23.68%), P3 comprised of 19 genotypes (25%), and P4 comprised of 18 (23.68%) genotypes. The average gene diversity between individuals in the same cluster was 0.112, 0.192, 0.223, and 0.251 for P1, P2, P3, and P4, respectively. The mean Fst values within P1, P2, P3, and P4 were 0.555, 0.366, 0.329, and 0.177.

## Discussion

### Mean Performance of Different Traits Under Irrigated and Stress Conditions

Sesame is one of the important oil seed crops grown for the high-quality oil. The cultivation of sesame is mostly confined to the arid/semi-arid regions where it is often exposed to prolonged water deficit. Maintaining the yield potential of sesame genotypes under WS conditions is important given that sesame crops are generally grown under rainfed conditions and often experience WS. Therefore, evaluating the yield attributing traits of sesame genotypes and their associations with yield under WS is critically important. The availability of this information is must to maintain and improve the crop production under water deficient environment. Here, we experimentally characterized the variation in phenotypic and genotypic traits in 76 sesame accessions over the two consecutive years (2018 and 2019). ANOVA revealed that genotypes differed significantly in most of the traits under WW and WS conditions. Our results showed that the NDM was lower in both the years under WS. These genotypes displayed the drought avoidance/escape under WS (Kumar and Abbo, 2001).

Drought in sesame has been shown to reduce yield by 28% and negatively affect yield attributing traits (Kassab et al., 2005; Mensah et al., 2006; Golestani and Pakniyat, 2007; Heidari et al., 2011). Similarly, our results showed that drought decreased SWP by 36–38% under WS during both years compared with WW. The scarcity of water during the reproductive phase reduces SWP. In addition, a severe reduction in yield attributing traits such as NCP (37%), CWP (44%), and TW (13%) was also observed under WS. The sesame crop is more prone to drought stress during reproductive phase in comparison to vegetative phase. Onset of drought during flowering and anthesis drastically damages the yield quantity and quality (Sun et al., 2010). The number of capsules is decreased under WS in sesame (Nadeem et al., 2015; Dossa et al., 2017b; Ozkan, 2018). WS at flowering results in a considerable reduction in capsule production and capsule dry weight up to 90% (Amani et al., 2012). Our results also show that WS caused a reduction in TW, which is consistent with the results of studies of sunflower and other oilseed crops (Razi and Assad, 1999; Seyni et al., 2010; Golestani and Pakniyat, 2015; Dissanayake et al., 2017). This reduction in SWP could stem from limitations in the translocation, availability, and utilization of assimilates (Badiel et al., 2017) induced by WS.

Under WS, the traits PH, NBP, LA, LDW, TDM, and PST varied to different degrees; for example, the 17% reduction in PH under WS observed in our study is similar to the findings of previous studies (Hassanzadeh et al., 2009; Heidari et al., 2011) showing that PH is reduced under WS in sesame. Likewise, NBP was reduced by 16–17% under WS, which stemmed from the reduction in PH under WS. A decrease in NBP under WS during our experiments in sesame is consistent with the results of previous studies of sesame (Amani et al., 2012; Zehra et al., 2017). Moisture stress has been shown to decrease LA and plant dry biomass production in sesame (Mensah et al., 2006). A drastic reduction in LA, LDW, TDM, and PST was observed under WS. LA decreased by almost 31–32%, LDW by almost 33.61%, TDM by 26%, and PST by almost 36–37%. These results are similar to the findings of Alizadeh (2002). Furthermore, Bhargava and Sawant (2013) studied the mechanism by which water stress affects sesame growth and found that a decrease in leaf expansion might be related to decreased growth, and this decrease in LA stemming from a reduction in cell division, elongation, and differentiation is related to water availability. These results are also consistent with the findings of Bahrami et al. (2012), Nadeem et al. (2015), Boureima et al. (2016), Badiel et al. (2017), Zehra et al. (2017), and Ozkan (2018). The plants are known to be sensitive to water shortages and greatly affected by reductions in turgor pressure (Taiz and Zeiger, 2006). Severe water deficiency conditions restrict water flow from the xylem to the surrounding cells, leading to impaired cell division, cell elongation, and cell differentiation (Hussain et al., 2008; Bhargava and Sawant, 2013). Thus, drought stress severely inhibits the growth and development of sesame and reduces dry weight, resulting in yield losses.

Relative water content of plant greatly influences the plant–water relation. The crops under drought conditions show a visible reduction in the RWC of leaves where in drought stressed plants have lower RWC in comparison to properly irrigated crops (Lata et al., 2011). Leaf RWC is also considered as one of the most important physiological/biochemical parameters for indicating the intensity of stress (Alizadeh, 2002). The maintained RWC under WS conditions indicates the ability of osmotic adjustment to maintain cell turgescence and physiological activities (Bayoumi et al., 2008). Yield reduction is also directly related to water stress conditions, as plants use stomatal closure to protect themselves from water loss through transpiration (Taiz and Zeiger, 2009). This stomatal closure results into compromised CO_2_ supply required in mesophyll cells (Chaves et al., 2009). In present study, we observed almost 14% reduction in RWC under WS conditions; the similar findings have been reported in case of common bean (*Phaseolus vulgaris* L.) (Ramos et al., 2003) and sesame (Fazeli et al., 2006). We noticed that most of the parameters showed a significant reduction that under WS condition in present study, however, an increase was observed in case of CT and SPAD. The SPAD increased by almost 12–13% under WS; Khayatnezhad et al. (2011) and Nabateregga et al. (2018) reported the similar observations in *P. vulgaris* and in maize, respectively. Plants grown under WS have higher chlorophyll content (Shahriari, 1999), and drought-tolerant lines have higher chlorophyll content compared with drought-sensitive genotypes (Alaei, 2011). CT has also been considered as an important trait for drought-tolerant genotypes (Ainsworth and Rogers, 2007; Blum, 2009). The CT is highly dependent on external environmental factors, which might mask its underlying genetic variation. In present study, we found that sesame accessions had higher CT (11–12%) under WS than under WW. WS led to a reduction in stomatal conductance and an increase in leaf temperature (Sehgal et al., 2017). Overall, reduced stomatal conductance and increased leaf temperature loosen the stomata and lower carbon fixation efficiency (Guendouz et al., 2012). Genotypes with lower CTs had higher SWP, consistent with the results of previous studies (Blum, 2009; Beebe et al., 2013).

### Association of Traits With Seed Yield and Selection of Genotypes for Drought Tolerance

The relationship between the different traits shown in Supplementary Tables 4, 5. SWP was strongly related to NBP, LA, LDW, TDM, PST, CT, and HI under WW and WS, indicating that these traits directly affect sesame SWP. These results are in line with the findings of previous studies (Gharib-Eshghi et al., 2016; Anastasi et al., 2017; Saljooghianpour and Javadzadeh, 2018). A stronger relationship with NBP and NCP indicates that these are important secondary traits contributing to the final SWP of sesame. The yield of sesame plants is highest in highly branched plants with higher NCP (Pham et al., 2010; Abdou et al., 2015; Fiseha et al., 2015). SWP was negatively related to TDM and CT, suggesting that genotypes with lower CTs have higher yields under WS. The results of Blum (2009) and Khan et al. (2011) in soybean and Beebe et al. (2013) are consistent with these findings. Considering both conditions (WW and WS) and seasons, NBP, LA, LDW, TDM, PST, CT, and HI were highly correlated with SWP and could be used as important secondary traits in sesame improvement programs under WS as well as under WW.

An effective selection for better performance entirely depends upon the identification of various associated traits (Chrigui et al., 2021). The path analysis helps in identify the important morpho-physiological and yield attributing traits despite the lack of their direct correlation with yield. Our findings suggested that the traits such as TDM, NBP, HI, NCP, LA, and LDW were associated with seed yield under both WW and WS conditions. These results are found in close conformity with the findings of Goudappagoudra et al. (2011), Gangadhara et al. (2012), Ibrahim and Khidir (2012), Fazal et al. (2015), and Gogoi and Sarma (2019), indicating the importance of these characters for consideration in the selection program.

### Multivariate Analysis

The vectors for different traits had small angles, which indicated that the traits were positively correlated. The traits NDF, NDM, LA, SPAD, NCP, SWP, and HI showed the highest degree of variation among years and conditions and may be further used to select genotypes. The combination of these indices provides more reliable estimates relative to independent indices for sesame drought tolerance. As the study material consists of genotypes, landraces, and elite lines adapted to various agro-ecological conditions, the clustering of these genotypes was not associated. However, they were grouped based on morphology. This might stem from the re-location of genotypes and the fact that environmental conditions may direct gene flow between populations from diverse geographical origins. However, 64 sunflower accessions were grouped into nine clusters, indicating the absence of a relationship between genetic and geographic diversity (Banumathy et al., 2010; Reddy et al., 2012).

### Genetic Diversity in Sesame Accessions

Genetic diversity in genotypes is the basis for crop breeding programs. In this study, the genetic diversity of 76 sesame genotypes was determined using 48 genome-wide SSR markers. SSR markers are considered ideal for genetic diversity studies of crops (UshaKiran et al., 2015). Sesame genotypes have been characterized using SSR markers developed by Li-Bin et al. (2008), Wei et al. (2011), Spandana et al. (2012), Wang et al. (2012), Badri et al. (2014), and Wei et al. (2014), but most sesame genotypes have not yet been genetically mapped. The genetic diversity results showed that SSR allelic diversity in sesame genotypes was low (*N*_*A*_ = 2.31, He = 0.25, and PIC = 0.22). Similarly, a previous study indicated that the number of SSR alleles with an average of 2.31 per locus in a collection of 24 cultivated sesame accessions (Zhang et al., 2012). PIC values indicated that only two out of 48 primer pairs (SIM19 and SIM53) showed high PIC values, which may be more useful for diagnostic applications in sesame. Low SSR polymorphism in sesame is also evident from previous studies. Wu et al. (2014) reported an average of 3.69 alleles per SSR locus among 130 sesame cultivars, landraces, and wild germplasm. Higher PIC values were reported by Cho et al. (2011) and Yue et al. (2012). Pandey et al. (2015) reported an average of 3.37 alleles per SSR locus and a PIC value of 0.57 in a collection of 60 sesame genotypes. Sehr et al. (2016) also observed low SSR polymorphism (PIC:0.26 and He:0.39) in a collection that included 121 accessions of Ugandan sesame landraces. Surapaneni et al. (2014) reported an average N_*A*_ of 2.5 and PIC of 0.66 in a collection of 68 sesame accessions using 72 polymorphic SSR markers. The level of polymorphism is influenced by the number of markers, population size, and the type of plant material studied. Nevertheless, low SSR polymorphism may stem from the lack of outcrossing between the genotypes studied. Some studies have indicated that the low polymorphism of SSR markers in sesame stems from its narrow genetic basis (Dixit et al., 2005; Wei et al., 2008). Furthermore, given that sesame is a self-pollinated crop, low genetic diversity may arise from the limited gene flow. Low polymorphism in sesame is a concern because this would reduce their use in genetic mapping.

Neighbor-joining tree analysis revealed at least three possible major clusters (G1, G2, and G3) in the genotypes. Several sub-clusters within the major clusters were also clearly observable. The genotypes were distributed across all three clusters. These results indicated that there was no relationship between genotypic clusters and the geographical distribution of these genotypes. The genotypes IC-204679 (Kerala) and IC-205206 (Maharashtra) were the closest, whereas IC-132387 (Punjab) and IC-204747 (Maharashtra) were the most divergent based on simple matching coefficients. The range of simple matching coefficients within G1, G2, and G3 suggested that substantial diversity existed within these clusters. The dendrogram did not place sesame accessions into clear groupings. Landraces from different areas within India are distributed in all of the clusters. Studies of the genetic diversity of sesame using diverse germplasm collections (wild species, landraces, and cultivars) across several countries have characterized the distribution of the accessions based on geographical sources (Laurentin and Karlovsky, 2006).

### Population Structure in the Sesamum Accessions

Population analysis helps to assess the patterns of genetic structure in a subset of samples (Porras-Hurtado et al., 2013). The analysis of structures revealed four populations with 76 sesamum genotypes. In addition to the accessions that belonged to a single population, that is, greater than 90.7% of their inferred ancestry was derived from one of the model-based populations, 43 accessions (9.21%) in the sample were categorized as belonging to admixed group. Populations were considered to have little differentiation when FST < 0.05, moderate differentiation when 0.05 < FST < 0.15, strong differentiation when 0.15 < FST < 0.25, and very strong differentiation when FST > 0.25 (Cho et al., 2011). In our study, the Fst values P1, P2, and P3 within the main populations were high, showing very strong differentiation of genotypes and P4 have moderate differentiation (FST = 0.11). Both the NJ tree has grouped the genotypes into 3 main clusters in which cluster 1 has 2 major sub groups and STRUCTURE grouped the genotypes into 4 major clusters that were almost comparable. The most divergent population P3 detected by STRUCTURE consisted of 19 accessions, which were also present in cluster I of diversity studies. Cho et al. (2011) reported three groups based on a model-based structure analysis, which was consistent with the clustering based on genetic distance. Similarly Dossa et al. (2016) reported three groups among 150 sesamum accessions collected from 22 countries. Basak et al. (2019) also studied genetic diversity and population structure in Mediterranean sesamum core collection of 103 accessions.

## Conclusion

The traits associated with SWP under WW and WS differed among the sesame genotypes studied. The genotypes were indigenous, and local selection and landraces with different genetic backgrounds resulted in trait variation. Variation in the specific traits measured among genotypes under WS aids the selection of these traits. These trait-specific genotypes could be used in sesame breeding programs to develop location-specific varieties. The values of most of the traits were reduced under WS, and the maximum values of traits were either positively or negatively associated with SWP under WS in both years. The observed patterns of phenotypic and genetic variation may stem from the geographical sources of genotypes, limited gene flow, and the low polymorphism of sesame given that it is a self-pollinated crop.

## Data Availability Statement

The original contributions presented in the study are included in the article/Supplementary Material, further inquiries can be directed to the corresponding author.

## Author Contributions

BP worked on sesame drought tolerance, recorded, and analyzed the experimental data. PR designed and executed the experiments, manuscript preparation. UK designed the primers, marker development, and diversity analysis. MD helped in cluster, PCA, and R statistical analysis. GL helped in yield measurements for a single season. KR and AG contributed to manuscript refinement. All authors contributed to the article and approved the submitted version.

## Conflict of Interest

The authors declare that the research was conducted in the absence of any commercial or financial relationships that could be construed as a potential conflict of interest.

## Publisher’s Note

All claims expressed in this article are solely those of the authors and do not necessarily represent those of their affiliated organizations, or those of the publisher, the editors and the reviewers. Any product that may be evaluated in this article, or claim that may be made by its manufacturer, is not guaranteed or endorsed by the publisher.

## Footnotes

1
http://taylor0.biology.ucla.edu/structureHarvester/

## Abbreviations

WW: Well-watered
WS: Terminal drought
NDF: Number of days to flower
NDM: Number of days to maturity
PH: Plant height
NBP: Number of branches
LA: Leaf area
LDW: Leaves dry weight
TDM: Total dry matter
PST: Plant stem weight
SPAD: chlorophyll meter readings
CT: Canopy temperature
NCP: Number of capsules plant^–1^
TW: Test weight
CWP: Capsules weight plant^–1^
SWP: seed yield plant^–1^
HI: Harvest index
PIC: Polymorphic information content
N_*A*_: Number of alleles.
