# Supplementary material for: Identifying Traits Associated With Terminal Drought Tolerance in Sesame (Sesamum indicum L.) Genotypes
Source: Front Plant Sci. 2021 Dec 10;12:739896. doi: 10.3389/fpls.2021.739896 (PMC8709571; doi:10.3389/fpls.2021.739896)
Supplement: Supplementary file 1 [file Data_Sheet_1.docx]

**Supplementary Table 1** List of sesame genotypes along with two checks used during two season study

| **S. No.** | **Genotypes** | **Source** | **Nature** | **S. No.** | **Genotypes** | **Source** | **Nature** |
| --- | --- | --- | --- | --- | --- | --- | --- |
| 1 | IC 110221 | Odisha | Landraces | 38 | IC 204706 | Maharashtra | Landraces |
| 2 | IC 131497 | Maharashtra | Landraces | 39 | IC 204747 | Maharashtra | Landraces |
| 3 | IC 131500 | Punjab | Landraces | 40 | IC 204753 | Maharashtra | Landraces |
| 4 | IC 131546 | Maharashtra | Landraces | 41 | IC 204789 | Maharashtra | Landraces |
| 5 | IC 131559 | Maharashtra | Landraces | 42 | IC 204842 | Maharashtra | Landraces |
| 6 | IC 131878 | Maharashtra | Landraces | 43 | IC 204849 | Maharashtra | Landraces |
| 7 | IC 131936 | Maharashtra | Landraces | 44 | IC 204861 | Maharashtra | Landraces |
| 8 | IC 131943 | Maharashtra | Landraces | 45 | IC 205206 | Maharashtra | Other |
| 9 | IC 131953 | Maharashtra | Landraces | 46 | IC 205209 | Maharashtra | Other |
| 10 | IC 132167 | Madhya Pradesh | Landraces | 47 | IC 205285 | Andhra Pradesh | Other |
| 11 | IC 132171 | Tamil Nadu | Landraces | 48 | IC 205304 | Uttar Pradesh | Landraces |
| 12 | IC 132176 | Tamil Nadu | Landraces | 49 | IC 205311 | Maharashtra | Landraces |
| 13 | IC 132186 | Tamil Nadu | Landraces | 50 | IC 205353 | Uttar Pradesh | Landraces |
| 14 | IC 132207 | Uttar Pradesh | Landraces | 51 | IC 205363 | Uttar Pradesh | Landraces |
| 15 | IC 132293 | Arunachal Pradesh | Landraces | 52 | IC 205471 | Himachal Pradesh | Landraces |
| 16 | IC 132300 | Arunachal Pradesh | Landraces | 53 | IC 205476 | Himachal Pradesh | Landraces |
| 17 | IC 132383 | Punjab | Landraces | 54 | IC 205479 | Kerala | Landraces |
| 18 | IC 132386 | Punjab | Landraces | 55 | IC 205724 | Rajasthan | Primitive cultivar |
| 19 | IC 132387 | Punjab | Landraces | 56 | IC 205791 | Rajasthan | Primitive cultivar |
| 20 | IC 132389 | Punjab | Landraces | 57 | IC 23279 | Maharashtra | Landraces |
| 21 | IC 132410 | Punjab | Landraces | 58 | IC 23297 | Maharashtra | Other |
| 22 | IC 132558 | Maharashtra | Landraces | 59 | IC 41920 | Maharashtra | Landraces |
| 23 | IC 16244 | Maharashtra | Landraces | 60 | IC 42999 | Rajasthan | Landraces |
| 24 | IC 17476-1 | Manipur | Landraces | 61 | IC 43033 | Rajasthan | Landraces |
| 25 | IC 203962 | Uttar Pradesh | Elite line | 62 | IC 43036 | Rajasthan | Landraces |
| 26 | IC 204045 | Uttar Pradesh | Landraces | 63 | IC 54035 | Uttarkhand | Landraces |
| 27 | IC 204046 | Uttar Pradesh | Landraces | 64 | IC 73576 | Tamil Nadu | Landraces |
| 28 | IC 204280 | Maharashtra | Landraces | 65 | IC 74188 | Tamil Nadu | Release variety |
| 29 | IC 204300 | Rajasthan | Elite line | 66 | IC 81564 | Assam | Other |
| 30 | IC 204406 | Rajasthan | Elite line | 67 | IC 96116 | Maharashtra | Other |
| 31 | IC 204445 | Maharashtra | Landraces | 68 | IC 96126 | Bihar | Other |
| 32 | IC 204545 | Kerala | Landraces | 69 | IC 96127 | Maharashtra | Other |
| 33 | IC 204572 | Maharashtra | Landraces | 70 | IC 96229 | Karnataka | Other |
| 34 | IC 204611 | Maharashtra | Landraces | 71 | IC 96231 | Karnataka | Landraces |
| 35 | IC 204666 | Maharashtra | Other | 72 | IC 96232 | Karnataka | Landraces |
| 36 | IC 204677 | Maharashtra | Landraces | 73 | IC 96233 | Karnataka | Landraces |
| 37 | IC 204679 | Kerala | Landraces | 74 | IC 96240 | Karnataka | Landraces |
| Check | GT-10 | Gujarat |  | Check | TKG-22 | Madhya Pradesh | Landraces |

**Supplementary Table 2** Mean performance of accessions including two checks (GT-10 and TKG-22) under WW condition during the year 2018 and 2019

| **S. No.** | **Genotypes** | **NDF** | **NDM** | **PH**  **(cm)** | **NBP** | **LA (cm^2^)** | **LDW (g)** | **TDM (g)** | **PST**  **(g)** | **SPAD** | **CT**  **(^0^C)** | **RWC**  **(%)** | **SWP**  **(g)** | **NCP** | **TW**  **(g)** | **CWP**  **(g)** | **HI**  **(%)** |
| --- | --- | --- | --- | --- | --- | --- | --- | --- | --- | --- | --- | --- | --- | --- | --- | --- | --- |
| 1 | GT-10 | 43.83 | 106.83 | 79.90 | 5.69 | 821.27 | 8.60 | 20.57 | 10.35 | 49.60 | 28.84 | 76.28 | 6.66 | 116.53 | 3.91 | 7.78 | 24.49 |
| 2 | IC 110221 | 41.50 | 98.33 | 72.57 | 5.70 | 675.81 | 5.66 | 21.65 | 9.82 | 45.74 | 29.63 | 75.71 | 8.17 | 97.82 | 3.76 | 8.98 | 27.69 |
| 3 | IC 131497 | 42.66 | 97.83 | 70.85 | 3.90 | 469.43 | 6.53 | 27.47 | 12.86 | 45.02 | 29.82 | 64.91 | 6.54 | 63.53 | 3.15 | 9.44 | 18.86 |
| 4 | IC 131500 | 43.99 | 103.83 | 79.15 | 6.00 | 753.71 | 8.45 | 21.27 | 10.02 | 49.18 | 29.88 | 76.30 | 8.08 | 108.05 | 3.02 | 9.02 | 27.55 |
| 5 | IC 131546 | 37.83 | 92.17 | 71.53 | 5.53 | 642.16 | 7.87 | 22.85 | 11.99 | 54.57 | 29.73 | 71.49 | 8.39 | 84.25 | 3.77 | 7.73 | 26.94 |
| 6 | IC 131559 | 41.66 | 101.50 | 67.80 | 4.50 | 552.16 | 7.68 | 23.59 | 11.51 | 50.27 | 28.65 | 71.29 | 6.03 | 91.58 | 3.90 | 11.10 | 20.44 |
| 7 | IC 131878 | 43.00 | 103.83 | 67.68 | 5.95 | 836.89 | 9.40 | 18.81 | 8.94 | 47.83 | 30.28 | 86.77 | 9.08 | 113.05 | 3.73 | 10.49 | 32.84 |
| 8 | IC 131936 | 38.17 | 92.17 | 73.45 | 5.15 | 687.18 | 5.51 | 21.11 | 10.81 | 52.04 | 29.67 | 79.54 | 7.70 | 84.03 | 3.45 | 7.59 | 28.33 |
| 9 | IC 131943 | 43.50 | 104.16 | 79.73 | 4.60 | 581.05 | 6.55 | 24.48 | 11.36 | 46.09 | 29.76 | 69.17 | 5.76 | 97.82 | 3.88 | 9.65 | 19.07 |
| 10 | IC 131953 | 40.33 | 92.83 | 71.13 | 5.05 | 614.22 | 6.74 | 21.64 | 10.36 | 53.99 | 30.87 | 74.86 | 6.55 | 96.15 | 3.51 | 8.34 | 23.31 |
| 11 | IC 132167 | 41.33 | 97.66 | 71.34 | 4.50 | 486.04 | 4.94 | 25.49 | 11.65 | 47.30 | 30.26 | 73.03 | 6.01 | 76.95 | 3.97 | 8.07 | 19.10 |
| 12 | IC 132171 | 38.66 | 97.33 | 70.60 | 5.90 | 770.85 | 8.94 | 20.89 | 11.58 | 51.37 | 30.18 | 75.23 | 8.52 | 93.47 | 3.86 | 9.72 | 28.94 |
| 13 | IC 132176 | 41.33 | 99.00 | 76.53 | 4.80 | 664.05 | 5.50 | 25.83 | 11.82 | 52.67 | 30.78 | 78.08 | 8.17 | 95.39 | 3.99 | 8.69 | 24.40 |
| 14 | IC 132186 | 36.66 | 91.33 | 73.55 | 4.35 | 484.33 | 5.79 | 24.10 | 11.04 | 48.59 | 30.02 | 73.40 | 6.70 | 76.80 | 3.26 | 9.07 | 22.14 |
| 15 | IC 132207 | 41.00 | 97.00 | 68.20 | 3.55 | 488.58 | 4.23 | 26.12 | 13.79 | 48.33 | 30.28 | 64.75 | 5.71 | 63.50 | 3.75 | 10.35 | 17.77 |
| 16 | IC 132293 | 42.50 | 95.83 | 78.52 | 5.60 | 769.57 | 8.05 | 19.60 | 9.40 | 47.24 | 29.53 | 78.77 | 8.06 | 95.30 | 3.10 | 9.31 | 29.16 |
| 17 | IC 132300 | 43.50 | 99.33 | 76.15 | 5.30 | 645.51 | 7.33 | 21.25 | 10.23 | 48.40 | 30.28 | 81.03 | 7.64 | 106.50 | 4.06 | 7.06 | 26.47 |
| 18 | IC 132383 | 42.00 | 100.83 | 71.60 | 4.55 | 604.07 | 5.52 | 23.99 | 11.86 | 45.00 | 29.53 | 83.29 | 7.10 | 93.38 | 3.74 | 7.76 | 23.08 |
| 19 | IC 132386 | 41.33 | 98.33 | 82.05 | 5.85 | 934.14 | 7.83 | 19.79 | 9.51 | 43.40 | 29.78 | 77.98 | 8.97 | 105.10 | 3.73 | 8.19 | 31.40 |
| 20 | IC 132387 | 41.33 | 98.16 | 81.58 | 5.65 | 644.68 | 7.26 | 23.71 | 11.73 | 50.97 | 30.09 | 76.07 | 7.09 | 99.80 | 3.76 | 8.94 | 23.35 |
| 21 | IC 132389 | 36.16 | 94.66 | 70.44 | 5.40 | 638.85 | 6.17 | 20.58 | 11.40 | 49.93 | 29.98 | 73.59 | 7.89 | 111.93 | 3.76 | 6.89 | 27.53 |
| 22 | IC 132410 | 46.00 | 98.66 | 76.30 | 5.60 | 840.86 | 8.33 | 20.84 | 10.36 | 46.89 | 29.61 | 78.99 | 8.53 | 118.58 | 3.92 | 8.85 | 29.09 |
| 23 | IC 132558 | 44.17 | 103.83 | 70.88 | 5.35 | 624.91 | 7.26 | 21.92 | 11.77 | 48.29 | 28.20 | 71.44 | 7.24 | 96.80 | 3.37 | 10.57 | 24.83 |
| 24 | IC 16244 | 40.83 | 93.50 | 86.88 | 5.90 | 876.93 | 7.66 | 18.93 | 10.43 | 46.84 | 30.77 | 79.92 | 9.68 | 115.00 | 4.16 | 9.79 | 34.29 |
| 25 | IC 17476-1 | 41.83 | 94.50 | 78.25 | 5.25 | 644.76 | 6.68 | 24.33 | 11.59 | 44.92 | 29.20 | 72.67 | 7.74 | 68.00 | 4.29 | 9.60 | 24.45 |
| 26 | IC 203962 | 41.16 | 97.17 | 66.64 | 4.55 | 582.26 | 7.18 | 26.21 | 12.50 | 50.68 | 29.66 | 74.51 | 5.99 | 86.30 | 3.85 | 10.77 | 18.75 |
| 27 | IC 204045 | 39.33 | 96.33 | 78.10 | 5.70 | 866.52 | 9.28 | 19.97 | 9.84 | 45.12 | 29.56 | 73.96 | 7.93 | 94.85 | 3.77 | 9.09 | 28.40 |
| 28 | IC 204046 | 40.66 | 98.50 | 72.85 | 4.40 | 604.04 | 6.53 | 24.84 | 11.72 | 49.69 | 30.64 | 76.57 | 7.15 | 67.90 | 3.93 | 9.89 | 22.57 |
| 29 | IC 204280 | 41.83 | 97.17 | 63.20 | 4.95 | 476.17 | 5.58 | 24.73 | 11.26 | 51.77 | 29.99 | 73.47 | 7.07 | 72.35 | 3.17 | 11.02 | 22.14 |
| 30 | IC 204300 | 38.33 | 95.17 | 69.20 | 4.85 | 801.05 | 8.03 | 24.69 | 12.77 | 48.08 | 28.82 | 76.93 | 7.67 | 72.50 | 3.45 | 9.60 | 24.19 |
| 31 | IC 204406 | 36.80 | 92.17 | 61.69 | 4.10 | 712.40 | 8.64 | 21.07 | 9.00 | 42.04 | 30.97 | 76.79 | 7.10 | 73.56 | 2.97 | 7.90 | 25.21 |
| 32 | IC 204445 | 36.83 | 92.00 | 71.40 | 5.55 | 706.95 | 6.17 | 19.86 | 10.33 | 46.99 | 28.83 | 78.02 | 8.28 | 97.45 | 3.63 | 9.44 | 29.88 |
| 33 | IC 204545 | 41.50 | 95.00 | 71.93 | 5.05 | 629.08 | 6.72 | 23.95 | 13.77 | 43.63 | 29.19 | 67.89 | 6.32 | 102.35 | 3.34 | 7.35 | 21.10 |
| 34 | IC 204572 | 40.83 | 97.50 | 67.92 | 4.40 | 593.76 | 6.13 | 24.53 | 11.15 | 49.99 | 29.50 | 75.38 | 5.84 | 93.75 | 3.52 | 9.88 | 19.21 |
| 35 | IC 204611 | 43.00 | 99.66 | 76.08 | 5.78 | 783.51 | 8.73 | 20.53 | 9.93 | 52.87 | 29.88 | 66.00 | 8.12 | 91.78 | 3.91 | 9.59 | 28.38 |
| 36 | IC 204666 | 42.83 | 94.50 | 82.03 | 5.20 | 637.51 | 6.14 | 22.42 | 10.76 | 46.80 | 30.19 | 69.39 | 7.04 | 80.78 | 3.92 | 9.38 | 23.95 |
| 37 | IC 204677 | 44.50 | 100.83 | 81.10 | 5.75 | 770.04 | 8.16 | 20.45 | 10.17 | 49.72 | 29.79 | 74.22 | 8.03 | 90.10 | 3.78 | 11.57 | 28.18 |
| 38 | IC 204679 | 43.17 | 98.50 | 73.88 | 6.15 | 947.71 | 8.61 | 17.93 | 9.14 | 50.08 | 29.42 | 81.24 | 9.69 | 84.93 | 3.56 | 9.97 | 35.24 |
| 39 | IC 204706 | 36.33 | 96.67 | 78.50 | 5.25 | 688.68 | 6.68 | 22.68 | 11.11 | 41.94 | 29.10 | 72.58 | 7.82 | 89.75 | 3.37 | 8.87 | 25.53 |
| 40 | IC 204747 | 35.99 | 91.50 | 75.45 | 4.80 | 649.16 | 6.73 | 21.70 | 10.56 | 50.03 | 30.12 | 76.01 | 7.01 | 77.78 | 3.69 | 8.37 | 24.20 |
| 41 | IC 204753 | 44.83 | 98.33 | 77.55 | 5.70 | 873.15 | 7.72 | 20.26 | 10.33 | 49.08 | 29.07 | 75.83 | 9.74 | 132.86 | 3.58 | 11.22 | 33.21 |
| 42 | IC 204789 | 43.33 | 102.83 | 77.75 | 5.05 | 796.37 | 7.10 | 25.03 | 10.81 | 46.75 | 29.45 | 68.02 | 7.85 | 97.67 | 3.22 | 9.85 | 23.47 |
| 43 | IC 204842 | 38.83 | 98.00 | 63.05 | 4.85 | 614.58 | 6.42 | 20.98 | 10.15 | 48.19 | 29.48 | 75.74 | 7.20 | 79.70 | 3.65 | 9.79 | 25.59 |
| 44 | IC 204849 | 41.00 | 94.16 | 70.25 | 4.75 | 560.73 | 5.91 | 23.97 | 12.61 | 52.15 | 30.32 | 78.96 | 7.84 | 77.86 | 4.19 | 8.63 | 25.29 |
| 45 | IC 204861 | 44.16 | 100.50 | 75.65 | 6.25 | 939.29 | 9.55 | 15.64 | 7.41 | 50.95 | 28.80 | 82.00 | 10.62 | 113.35 | 2.85 | 9.85 | 40.42 |
| 46 | IC 205206 | 44.66 | 103.33 | 68.15 | 4.75 | 623.57 | 7.24 | 24.71 | 12.81 | 50.18 | 29.87 | 82.49 | 7.38 | 93.45 | 4.00 | 8.87 | 23.43 |
| 47 | IC 205209 | 41.00 | 96.66 | 67.95 | 4.10 | 715.14 | 7.10 | 24.19 | 14.12 | 49.73 | 30.94 | 67.94 | 7.81 | 66.65 | 3.68 | 10.13 | 24.47 |
| 48 | IC 205285 | 41.33 | 98.00 | 72.95 | 4.25 | 501.81 | 5.86 | 25.76 | 13.21 | 43.84 | 29.50 | 66.91 | 5.98 | 74.61 | 4.52 | 9.56 | 18.87 |
| 49 | IC 205304 | 39.00 | 96.33 | 63.55 | 4.50 | 453.75 | 5.03 | 24.20 | 12.60 | 52.05 | 28.73 | 75.52 | 7.24 | 82.71 | 3.64 | 7.93 | 22.80 |
| 50 | IC 205311 | 41.17 | 92.00 | 70.75 | 5.80 | 711.96 | 7.15 | 23.70 | 11.44 | 48.57 | 29.06 | 67.91 | 7.13 | 99.15 | 2.92 | 9.97 | 23.21 |
| 51 | IC 205353 | 36.67 | 91.50 | 71.90 | 4.24 | 546.21 | 5.27 | 23.32 | 12.17 | 51.08 | 29.16 | 66.23 | 6.39 | 70.82 | 3.60 | 8.74 | 21.49 |
| 52 | IC 205363 | 39.00 | 91.50 | 67.80 | 5.55 | 803.83 | 6.95 | 19.86 | 9.29 | 49.18 | 30.00 | 81.01 | 8.41 | 73.46 | 3.28 | 8.73 | 30.41 |
| 53 | IC 205471 | 42.00 | 98.83 | 71.45 | 5.05 | 751.82 | 7.10 | 22.15 | 10.12 | 43.57 | 28.62 | 76.61 | 7.35 | 85.60 | 4.01 | 9.71 | 24.90 |
| 54 | IC 205476 | 41.00 | 99.67 | 69.75 | 4.65 | 541.24 | 6.52 | 26.52 | 13.92 | 42.85 | 29.48 | 87.84 | 6.14 | 82.30 | 3.98 | 10.07 | 19.76 |
| 55 | IC 205479 | 37.66 | 94.33 | 77.75 | 5.25 | 703.02 | 6.03 | 22.29 | 11.83 | 51.97 | 29.41 | 77.84 | 7.77 | 72.70 | 3.46 | 9.74 | 26.05 |
| 56 | IC 205724 | 38.83 | 98.50 | 69.75 | 4.44 | 690.39 | 8.25 | 18.49 | 9.89 | 43.71 | 30.90 | 80.33 | 6.34 | 62.53 | 2.67 | 6.68 | 25.53 |
| 57 | IC 205791 | 38.00 | 95.50 | 74.56 | 5.00 | 744.52 | 8.50 | 19.32 | 10.55 | 46.62 | 30.92 | 74.07 | 5.46 | 62.75 | 3.19 | 6.69 | 22.07 |
| 58 | IC 23279 | 40.50 | 96.33 | 83.40 | 4.90 | 673.53 | 6.90 | 23.01 | 10.93 | 51.90 | 28.95 | 68.26 | 7.35 | 73.15 | 3.68 | 8.81 | 24.14 |
| 59 | IC 23297 | 41.66 | 97.83 | 75.63 | 5.60 | 725.43 | 8.22 | 20.74 | 9.93 | 45.44 | 30.29 | 77.85 | 8.19 | 115.45 | 3.77 | 10.00 | 28.28 |
| 60 | IC 41920 | 45.50 | 111.33 | 89.40 | 5.40 | 712.32 | 7.84 | 20.60 | 10.31 | 37.60 | 30.01 | 72.98 | 7.65 | 126.30 | 3.80 | 9.52 | 27.11 |
| 61 | IC 42999 | 41.50 | 97.50 | 83.75 | 4.90 | 658.47 | 7.13 | 24.17 | 10.79 | 48.03 | 28.46 | 81.35 | 7.63 | 99.70 | 4.02 | 9.06 | 24.10 |
| 62 | IC 43033 | 43.83 | 102.33 | 79.62 | 6.10 | 800.52 | 8.48 | 24.47 | 11.07 | 45.00 | 30.33 | 79.68 | 8.15 | 104.60 | 3.58 | 9.53 | 25.04 |
| 63 | IC 43036 | 43.83 | 102.83 | 77.85 | 5.85 | 792.12 | 5.95 | 18.83 | 8.86 | 53.18 | 30.68 | 89.07 | 8.68 | 113.15 | 2.43 | 9.79 | 31.99 |
| 64 | IC 54035 | 41.83 | 97.00 | 77.52 | 4.95 | 664.99 | 6.16 | 22.71 | 10.41 | 47.44 | 29.12 | 80.07 | 7.28 | 91.23 | 3.72 | 8.37 | 25.31 |
| 65 | IC 73576 | 40.00 | 97.00 | 67.79 | 6.05 | 965.70 | 7.74 | 17.76 | 9.47 | 46.50 | 30.74 | 80.13 | 9.92 | 110.72 | 3.51 | 10.47 | 36.32 |
| 66 | IC 74188 | 38.79 | 97.67 | 72.75 | 4.69 | 719.04 | 7.05 | 15.83 | 9.67 | 46.21 | 30.59 | 82.60 | 6.95 | 83.36 | 3.23 | 7.92 | 30.48 |
| 67 | IC 81564 | 41.99 | 97.83 | 79.04 | 5.00 | 733.40 | 6.40 | 21.72 | 10.33 | 52.27 | 30.04 | 74.14 | 8.24 | 95.80 | 4.09 | 9.02 | 28.87 |
| 68 | IC 96116 | 37.83 | 90.83 | 75.37 | 5.40 | 653.56 | 6.02 | 22.20 | 10.25 | 48.87 | 30.03 | 80.39 | 7.67 | 93.05 | 3.62 | 11.01 | 25.71 |
| 69 | IC 96126 | 37.67 | 91.83 | 72.80 | 4.50 | 608.77 | 7.07 | 26.60 | 13.42 | 50.23 | 29.93 | 79.05 | 6.81 | 85.93 | 4.09 | 9.18 | 20.99 |
| 70 | IC 96127 | 37.67 | 91.67 | 80.08 | 4.75 | 580.62 | 6.05 | 18.60 | 9.42 | 47.72 | 29.47 | 69.42 | 7.14 | 81.85 | 3.29 | 9.14 | 27.80 |
| 71 | IC 96229 | 42.33 | 96.83 | 81.30 | 3.65 | 554.44 | 7.77 | 28.68 | 12.43 | 53.04 | 30.40 | 74.99 | 4.95 | 71.45 | 3.88 | 11.92 | 15.00 |
| 72 | IC 96231 | 36.16 | 90.66 | 78.14 | 4.15 | 590.98 | 7.23 | 20.14 | 10.34 | 49.82 | 29.47 | 76.91 | 6.62 | 86.20 | 4.01 | 8.70 | 24.78 |
| 73 | IC 96232 | 36.33 | 91.00 | 71.98 | 6.10 | 948.85 | 11.08 | 23.43 | 11.62 | 49.64 | 30.65 | 81.35 | 9.65 | 120.20 | 3.13 | 9.40 | 30.03 |
| 74 | IC 96233 | 40.78 | 93.79 | 70.54 | 5.26 | 722.20 | 7.34 | 16.03 | 9.92 | 46.96 | 30.79 | 82.51 | 5.41 | 75.63 | 3.65 | 8.09 | 25.24 |
| 75 | IC 96240 | 45.16 | 104.00 | 69.58 | 5.40 | 828.31 | 9.36 | 19.10 | 9.27 | 46.09 | 29.58 | 73.98 | 8.83 | 109.17 | 3.85 | 9.97 | 31.58 |
| 76 | TKG-22 | 43.33 | 97.50 | 84.05 | 5.60 | 761.58 | 7.60 | 20.36 | 9.15 | 39.40 | 28.71 | 78.96 | 6.31 | 105.70 | 3.57 | 10.91 | 23.81 |
|  | **Maximum** | 46.00 | 111.33 | 89.40 | 6.25 | 965.70 | 11.08 | 28.68 | 14.12 | 54.57 | 30.97 | 89.07 | 10.62 | 132.86 | 4.52 | 11.92 | 40.42 |
|  | **Minimum** | 35.99 | 90.66 | 61.69 | 3.55 | 453.75 | 4.23 | 15.64 | 7.41 | 37.60 | 28.20 | 64.75 | 4.95 | 62.53 | 2.43 | 6.68 | 15.00 |
|  | **Average** | 40.88 | 97.26 | 74.18 | 5.11 | 690.35 | 7.14 | 22.13 | 10.93 | 48.15 | 29.78 | 75.87 | 7.49 | 91.00 | 3.63 | 9.24 | 25.66 |

**Supplementary Table 3** Mean performance of accessions including two checks (GT-10 and TKG-22) under WS condition during the year 2018 and 2019

| **S. No.** | **Genotypes** | **NDF** | **NDM** | **PH**  **(cm)** | **NBP** | **LA (cm^2^)** | **LDW (g)** | **TDM (g)** | **PST**  **(g)** | **SPAD** | **CT**  **(^0^C)** | **RWC**  **(%)** | **SWP**  **(g)** | **NCP** | **TW**  **(g)** | **CWP**  **(g)** | **HI**  **(%)** |
| --- | --- | --- | --- | --- | --- | --- | --- | --- | --- | --- | --- | --- | --- | --- | --- | --- | --- |
| 1 | GT-10 | 42.83 | 97.50 | 75.73 | 5.30 | 552.90 | 4.22 | 12.34 | 6.34 | 57.32 | 32.42 | 75.68 | 4.00 | 72.75 | 2.57 | 5.70 | 24.56 |
| 2 | IC 110221 | 43.33 | 98.83 | 54.73 | 3.60 | 388.22 | 5.29 | 22.42 | 7.05 | 59.19 | 33.27 | 54.65 | 4.19 | 68.62 | 3.21 | 4.46 | 15.99 |
| 3 | IC 131497 | 41.50 | 92.66 | 61.93 | 4.05 | 363.23 | 3.51 | 19.14 | 8.87 | 53.51 | 34.61 | 71.38 | 4.06 | 66.08 | 2.76 | 5.23 | 17.47 |
| 4 | IC 131500 | 44.33 | 99.66 | 62.10 | 3.15 | 458.46 | 4.76 | 19.95 | 8.23 | 55.07 | 34.24 | 54.80 | 5.15 | 61.55 | 3.15 | 5.67 | 19.98 |
| 5 | IC 131546 | 41.16 | 92.83 | 55.64 | 4.35 | 577.78 | 5.37 | 17.78 | 10.87 | 54.27 | 33.82 | 73.45 | 5.25 | 81.35 | 3.21 | 7.97 | 23.65 |
| 6 | IC 131559 | 45.66 | 100.17 | 58.03 | 3.85 | 258.74 | 3.49 | 17.84 | 10.21 | 54.74 | 33.67 | 63.42 | 4.72 | 59.24 | 3.74 | 4.26 | 21.87 |
| 7 | IC 131878 | 43.83 | 98.16 | 72.33 | 4.42 | 463.51 | 5.11 | 14.85 | 10.32 | 57.23 | 33.87 | 68.33 | 4.53 | 60.58 | 3.06 | 6.50 | 23.46 |
| 8 | IC 131936 | 42.83 | 88.67 | 63.15 | 4.35 | 531.16 | 4.32 | 16.56 | 4.09 | 50.78 | 34.20 | 64.55 | 5.45 | 65.33 | 3.38 | 4.94 | 25.32 |
| 9 | IC 131943 | 42.50 | 94.50 | 64.72 | 4.20 | 355.61 | 5.15 | 15.43 | 4.56 | 54.72 | 33.27 | 64.50 | 4.91 | 63.13 | 3.36 | 8.12 | 24.17 |
| 10 | IC 131953 | 40.16 | 94.16 | 55.10 | 3.35 | 465.98 | 4.81 | 18.20 | 6.76 | 52.00 | 34.72 | 65.27 | 4.40 | 69.55 | 3.07 | 6.02 | 19.46 |
| 11 | IC 132167 | 42.33 | 91.50 | 56.20 | 3.65 | 444.34 | 4.64 | 17.64 | 7.63 | 54.40 | 33.08 | 61.07 | 4.67 | 62.80 | 3.63 | 5.77 | 20.92 |
| 12 | IC 132171 | 38.00 | 97.83 | 54.05 | 4.49 | 582.36 | 5.24 | 16.71 | 7.12 | 51.84 | 34.51 | 63.99 | 4.66 | 81.22 | 3.30 | 6.17 | 21.93 |
| 13 | IC 132176 | 41.67 | 94.83 | 60.15 | 3.90 | 404.64 | 3.95 | 17.12 | 6.81 | 60.51 | 33.13 | 66.89 | 3.55 | 71.80 | 3.24 | 4.49 | 17.10 |
| 14 | IC 132186 | 37.83 | 88.66 | 59.93 | 2.90 | 428.11 | 3.99 | 18.07 | 5.97 | 56.15 | 32.83 | 65.77 | 4.18 | 56.69 | 3.63 | 4.41 | 18.79 |
| 15 | IC 132207 | 43.17 | 96.50 | 56.35 | 3.85 | 374.74 | 3.84 | 19.29 | 10.74 | 49.33 | 34.28 | 44.76 | 2.51 | 51.32 | 2.72 | 6.74 | 13.32 |
| 16 | IC 132293 | 38.50 | 89.66 | 58.74 | 3.58 | 288.47 | 3.46 | 19.35 | 7.50 | 56.34 | 32.36 | 53.70 | 4.60 | 72.97 | 2.85 | 4.84 | 19.07 |
| 17 | IC 132300 | 41.83 | 95.33 | 57.57 | 3.40 | 415.04 | 3.98 | 17.43 | 5.82 | 50.99 | 33.80 | 58.80 | 4.12 | 62.42 | 3.43 | 5.73 | 19.19 |
| 18 | IC 132383 | 38.83 | 94.66 | 52.63 | 3.80 | 481.68 | 4.41 | 17.63 | 6.06 | 57.74 | 33.42 | 53.33 | 4.77 | 65.85 | 3.57 | 6.41 | 21.21 |
| 19 | IC 132386 | 42.33 | 101.00 | 68.88 | 4.05 | 477.41 | 5.51 | 20.47 | 6.16 | 52.79 | 34.24 | 57.22 | 4.39 | 67.70 | 3.15 | 5.23 | 17.79 |
| 20 | IC 132387 | 42.33 | 94.16 | 50.50 | 4.05 | 473.36 | 4.45 | 14.44 | 7.13 | 55.91 | 33.49 | 70.19 | 3.74 | 67.65 | 3.61 | 6.08 | 21.15 |
| 21 | IC 132389 | 39.33 | 95.83 | 62.72 | 3.40 | 282.36 | 5.32 | 18.18 | 7.20 | 56.81 | 32.48 | 60.44 | 4.50 | 62.33 | 3.38 | 5.15 | 20.55 |
| 22 | IC 132410 | 40.00 | 88.17 | 55.43 | 3.90 | 403.26 | 4.89 | 15.86 | 5.28 | 55.02 | 33.79 | 71.72 | 4.15 | 64.30 | 3.41 | 5.92 | 20.78 |
| 23 | IC 132558 | 40.33 | 94.00 | 69.99 | 4.00 | 436.97 | 3.81 | 17.21 | 8.26 | 50.88 | 33.37 | 62.44 | 5.19 | 51.93 | 2.79 | 6.07 | 25.56 |
| 24 | IC 16244 | 42.00 | 91.17 | 54.25 | 4.25 | 533.54 | 5.70 | 17.28 | 7.84 | 53.17 | 33.92 | 70.81 | 4.75 | 77.65 | 3.90 | 5.92 | 21.63 |
| 25 | IC 17476-1 | 41.83 | 94.66 | 66.95 | 4.05 | 383.99 | 4.56 | 15.14 | 7.24 | 56.42 | 33.43 | 74.52 | 4.58 | 68.20 | 2.93 | 5.47 | 23.46 |
| 26 | IC 203962 | 41.99 | 93.00 | 55.63 | 4.48 | 337.88 | 4.23 | 17.60 | 7.72 | 59.11 | 32.45 | 61.45 | 4.67 | 75.11 | 3.30 | 3.97 | 20.89 |
| 27 | IC 204045 | 40.33 | 93.16 | 64.15 | 5.45 | 679.89 | 5.68 | 16.14 | 7.46 | 48.13 | 32.85 | 74.09 | 6.63 | 73.85 | 3.59 | 5.44 | 30.99 |
| 28 | IC 204046 | 37.99 | 92.00 | 66.35 | 3.90 | 728.31 | 5.38 | 13.99 | 7.82 | 49.41 | 32.04 | 75.29 | 5.38 | 71.20 | 3.13 | 6.80 | 27.54 |
| 29 | IC 204280 | 41.00 | 94.00 | 62.15 | 4.55 | 505.40 | 3.94 | 14.42 | 8.82 | 56.06 | 33.69 | 73.28 | 4.78 | 76.61 | 3.52 | 4.67 | 24.77 |
| 30 | IC 204300 | 39.50 | 93.50 | 62.65 | 5.25 | 557.67 | 3.32 | 15.51 | 4.56 | 59.99 | 33.51 | 74.74 | 5.29 | 92.40 | 2.84 | 4.94 | 25.95 |
| 31 | IC 204406 | 37.00 | 88.30 | 49.25 | 3.70 | 409.18 | 6.46 | 15.63 | 6.87 | 49.61 | 32.48 | 66.95 | 5.29 | 62.73 | 2.87 | 7.46 | 25.29 |
| 32 | IC 204445 | 41.17 | 91.17 | 49.42 | 3.05 | 318.16 | 4.21 | 20.29 | 8.20 | 61.29 | 33.29 | 54.08 | 3.64 | 69.37 | 2.46 | 6.53 | 15.20 |
| 33 | IC 204545 | 41.67 | 92.83 | 54.35 | 4.80 | 491.73 | 4.45 | 12.90 | 4.62 | 52.49 | 35.04 | 66.58 | 4.52 | 79.05 | 2.90 | 4.34 | 25.83 |
| 34 | IC 204572 | 40.67 | 94.17 | 55.40 | 5.30 | 575.37 | 4.08 | 13.27 | 5.07 | 49.91 | 32.82 | 75.96 | 4.61 | 86.10 | 3.21 | 4.32 | 25.09 |
| 35 | IC 204611 | 43.17 | 97.17 | 61.58 | 3.85 | 352.51 | 3.52 | 17.23 | 11.37 | 55.48 | 33.24 | 63.57 | 3.99 | 70.69 | 3.83 | 4.94 | 19.71 |
| 36 | IC 204666 | 38.00 | 91.33 | 79.55 | 4.70 | 537.43 | 4.53 | 11.85 | 5.12 | 54.05 | 32.69 | 68.82 | 4.11 | 68.25 | 3.26 | 5.11 | 24.20 |
| 37 | IC 204677 | 40.50 | 91.50 | 74.03 | 3.90 | 330.59 | 3.72 | 21.55 | 8.00 | 51.16 | 33.63 | 60.90 | 4.95 | 67.35 | 3.16 | 4.13 | 18.43 |
| 38 | IC 204679 | 41.83 | 93.16 | 64.05 | 4.00 | 499.65 | 5.77 | 14.47 | 6.56 | 59.44 | 31.90 | 60.32 | 4.09 | 65.75 | 3.18 | 4.66 | 23.36 |
| 39 | IC 204706 | 41.00 | 91.33 | 67.45 | 4.00 | 661.59 | 5.53 | 15.83 | 4.44 | 56.85 | 34.31 | 80.11 | 4.50 | 78.83 | 3.23 | 4.30 | 22.44 |
| 40 | IC 204747 | 39.33 | 88.66 | 67.25 | 4.95 | 564.44 | 5.25 | 15.70 | 7.22 | 56.79 | 33.17 | 68.04 | 4.96 | 85.05 | 3.26 | 4.34 | 24.40 |
| 41 | IC 204753 | 42.50 | 95.67 | 56.70 | 4.45 | 465.17 | 3.88 | 13.84 | 8.44 | 55.08 | 32.81 | 64.76 | 4.81 | 69.78 | 3.72 | 5.60 | 25.77 |
| 42 | IC 204789 | 44.00 | 93.83 | 64.35 | 5.45 | 559.63 | 4.68 | 15.62 | 4.54 | 53.33 | 33.87 | 76.25 | 7.39 | 81.02 | 2.88 | 3.41 | 33.86 |
| 43 | IC 204842 | 40.83 | 96.00 | 68.90 | 5.35 | 447.20 | 4.14 | 14.64 | 3.80 | 49.65 | 31.55 | 74.89 | 6.57 | 68.58 | 3.72 | 4.63 | 31.01 |
| 44 | IC 204849 | 40.50 | 92.33 | 62.30 | 3.45 | 311.85 | 4.05 | 21.66 | 11.39 | 56.31 | 32.55 | 62.06 | 3.47 | 61.30 | 3.31 | 3.83 | 13.95 |
| 45 | IC 204861 | 41.17 | 97.16 | 63.35 | 5.25 | 488.78 | 4.29 | 13.27 | 6.98 | 55.62 | 30.94 | 64.99 | 5.60 | 70.35 | 3.53 | 5.18 | 29.91 |
| 46 | IC 205206 | 41.83 | 93.83 | 59.60 | 6.05 | 549.62 | 5.31 | 11.74 | 2.97 | 54.94 | 31.93 | 77.08 | 7.02 | 83.28 | 3.15 | 5.64 | 37.75 |
| 47 | IC 205209 | 42.67 | 97.50 | 68.85 | 3.95 | 518.45 | 5.43 | 14.06 | 8.03 | 54.60 | 32.05 | 74.13 | 5.03 | 67.75 | 2.41 | 5.98 | 26.55 |
| 48 | IC 205285 | 40.17 | 93.83 | 64.30 | 4.80 | 474.02 | 4.79 | 16.34 | 3.81 | 54.49 | 32.00 | 73.71 | 4.68 | 70.10 | 3.43 | 3.41 | 22.34 |
| 49 | IC 205304 | 40.83 | 95.83 | 60.25 | 3.20 | 560.28 | 5.77 | 12.41 | 7.42 | 51.67 | 33.73 | 76.14 | 4.95 | 73.60 | 3.34 | 4.99 | 28.01 |
| 50 | IC 205311 | 37.33 | 89.00 | 66.60 | 6.10 | 686.63 | 5.23 | 14.11 | 7.13 | 54.39 | 32.19 | 80.64 | 7.42 | 88.06 | 2.97 | 4.09 | 36.61 |
| 51 | IC 205353 | 41.33 | 91.16 | 63.95 | 5.55 | 616.93 | 4.62 | 11.82 | 6.90 | 56.63 | 32.33 | 66.45 | 5.28 | 77.13 | 3.41 | 6.39 | 30.45 |
| 52 | IC 205363 | 40.83 | 91.16 | 57.45 | 5.10 | 504.69 | 4.30 | 15.30 | 5.15 | 59.46 | 32.73 | 66.72 | 5.15 | 88.68 | 3.21 | 4.90 | 25.57 |
| 53 | IC 205471 | 41.83 | 95.00 | 65.35 | 4.30 | 562.87 | 4.32 | 21.74 | 6.73 | 49.33 | 32.91 | 57.76 | 5.36 | 67.50 | 3.10 | 6.08 | 20.15 |
| 54 | IC 205476 | 41.00 | 93.67 | 58.65 | 4.45 | 655.72 | 4.46 | 16.14 | 4.05 | 48.21 | 33.13 | 76.02 | 6.35 | 71.09 | 2.82 | 4.09 | 28.72 |
| 55 | IC 205479 | 40.66 | 95.17 | 67.40 | 4.35 | 472.55 | 4.24 | 21.19 | 4.63 | 56.46 | 33.69 | 66.88 | 3.42 | 73.60 | 2.82 | 4.93 | 14.35 |
| 56 | IC 205724 | 38.67 | 94.50 | 62.36 | 3.90 | 425.65 | 4.99 | 14.29 | 7.66 | 50.92 | 32.62 | 65.03 | 5.00 | 55.30 | 2.37 | 6.02 | 25.74 |
| 57 | IC 205791 | 39.50 | 91.50 | 66.73 | 4.45 | 407.45 | 5.37 | 15.27 | 9.82 | 48.73 | 31.78 | 65.28 | 4.55 | 51.37 | 2.75 | 5.61 | 22.99 |
| 58 | IC 23279 | 40.16 | 92.33 | 74.25 | 4.85 | 480.83 | 5.47 | 14.35 | 5.49 | 61.33 | 31.48 | 69.92 | 6.34 | 82.28 | 3.55 | 6.03 | 30.97 |
| 59 | IC 23297 | 41.83 | 93.33 | 56.15 | 3.35 | 355.23 | 2.97 | 16.17 | 9.96 | 52.99 | 33.45 | 48.20 | 4.56 | 56.55 | 2.85 | 5.48 | 24.62 |
| 60 | IC 41920 | 43.50 | 95.00 | 66.20 | 4.18 | 489.44 | 4.93 | 16.66 | 6.47 | 56.07 | 33.15 | 56.11 | 4.20 | 73.20 | 3.10 | 8.32 | 20.17 |
| 61 | IC 42999 | 42.33 | 94.83 | 61.40 | 3.20 | 481.16 | 4.82 | 20.96 | 6.63 | 54.83 | 32.72 | 65.36 | 5.01 | 65.55 | 3.35 | 6.54 | 18.96 |
| 62 | IC 43033 | 41.00 | 97.67 | 60.48 | 3.50 | 459.08 | 4.15 | 19.79 | 9.01 | 55.84 | 33.05 | 53.94 | 3.21 | 59.03 | 3.63 | 5.10 | 14.00 |
| 63 | IC 43036 | 44.33 | 100.66 | 59.40 | 5.13 | 404.81 | 5.75 | 17.20 | 7.18 | 56.41 | 33.89 | 61.72 | 4.77 | 71.15 | 3.49 | 3.76 | 21.79 |
| 64 | IC 54035 | 39.67 | 95.33 | 61.59 | 3.75 | 420.89 | 5.79 | 15.00 | 7.25 | 49.33 | 33.76 | 56.49 | 4.17 | 59.00 | 3.25 | 3.71 | 22.12 |
| 65 | IC 73576 | 41.00 | 94.83 | 60.77 | 4.45 | 465.77 | 6.05 | 16.29 | 4.84 | 57.33 | 33.99 | 60.82 | 5.15 | 81.28 | 2.70 | 5.73 | 24.19 |
| 66 | IC 74188 | 37.17 | 92.17 | 60.26 | 3.90 | 407.71 | 5.29 | 12.15 | 7.41 | 54.24 | 32.75 | 69.90 | 5.20 | 66.85 | 2.79 | 5.19 | 30.01 |
| 67 | IC 81564 | 38.67 | 92.16 | 52.91 | 5.25 | 354.28 | 5.38 | 17.93 | 7.17 | 59.83 | 34.34 | 67.97 | 5.57 | 62.81 | 3.62 | 4.92 | 23.86 |
| 68 | IC 96116 | 39.33 | 89.83 | 68.63 | 5.55 | 649.91 | 5.74 | 15.69 | 5.27 | 57.74 | 31.93 | 73.76 | 6.05 | 69.06 | 3.27 | 4.58 | 27.95 |
| 69 | IC 96126 | 36.16 | 88.50 | 63.90 | 4.75 | 646.18 | 4.84 | 12.05 | 10.07 | 48.88 | 32.25 | 77.50 | 5.53 | 73.83 | 2.86 | 3.63 | 31.28 |
| 70 | IC 96127 | 36.83 | 88.67 | 57.45 | 4.00 | 456.76 | 5.60 | 18.01 | 7.50 | 54.60 | 33.20 | 65.67 | 6.00 | 74.87 | 2.83 | 5.50 | 24.39 |
| 71 | IC 96229 | 40.33 | 90.83 | 64.52 | 4.30 | 422.78 | 5.57 | 18.89 | 7.65 | 53.37 | 33.92 | 56.39 | 4.39 | 64.67 | 2.83 | 4.95 | 18.93 |
| 72 | IC 96231 | 41.33 | 94.33 | 72.60 | 4.70 | 426.01 | 5.55 | 14.87 | 8.38 | 52.88 | 34.09 | 54.88 | 3.59 | 58.30 | 3.72 | 6.31 | 19.83 |
| 73 | IC 96232 | 39.16 | 91.67 | 56.05 | 4.25 | 455.85 | 4.55 | 17.43 | 9.39 | 57.29 | 33.52 | 63.95 | 4.81 | 84.20 | 2.79 | 4.47 | 21.82 |
| 74 | IC 96233 | 40.67 | 89.66 | 57.76 | 4.13 | 432.62 | 4.39 | 12.37 | 7.11 | 52.80 | 32.69 | 69.08 | 3.78 | 63.49 | 2.77 | 5.20 | 23.49 |
| 75 | IC 96240 | 43.50 | 97.16 | 55.29 | 4.55 | 383.37 | 5.25 | 20.14 | 8.92 | 51.70 | 33.27 | 61.90 | 5.41 | 71.60 | 2.90 | 6.49 | 20.74 |
| 76 | TKG-22 | 42.83 | 91.83 | 66.85 | 3.45 | 462.43 | 4.46 | 14.42 | 6.12 | 46.69 | 33.89 | 70.22 | 3.43 | 72.75 | 2.74 | 3.35 | 19.84 |
|  | **Maximum** | 45.66 | 101.00 | 79.55 | 6.10 | 728.31 | 6.46 | 22.42 | 11.39 | 61.33 | 35.04 | 80.64 | 7.42 | 92.40 | 3.90 | 8.32 | 37.75 |
|  | **Minimum** | 36.16 | 88.17 | 49.25 | 2.90 | 258.74 | 2.97 | 11.74 | 2.97 | 46.69 | 30.94 | 44.76 | 2.51 | 51.32 | 2.37 | 3.35 | 13.32 |
|  | **Average** | 40.91 | 93.66 | 61.77 | 4.28 | 470.24 | 4.74 | 16.40 | 7.09 | 54.35 | 33.17 | 65.90 | 4.81 | 69.79 | 3.17 | 5.30 | 23.22 |

**Supplementary Table 4** Correlation analysis of 16 phenotypic traits during the year 2018 and 2019 under WW condition

|  | **Year -2018** | | | | | | | | | | | | | | | |
| --- | --- | --- | --- | --- | --- | --- | --- | --- | --- | --- | --- | --- | --- | --- | --- | --- |
|  | **NDF** | **NDM** | **PH** | **NBP** | **LA** | **LDW** | **TDM** | **PST** | **SPAD** | **CT** | **RWC** | **SWP** | **NCP** | **TW** | **CWP** | **HI** |
| **NDF** | 1.000 | 0.414** | 0.283* | 0.077 | -0.029 | -0.044 | 0.073 | -0.033 | -0.177 | -0.181 | 0.040 | 0.002 | 0.308** | 0.054 | 0.004 | -0.026 |
| **NDM** |  | 1.000 | 0.074 | -0.061 | -0.139 | -0.004 | 0.169 | 0.096 | -0.049 | -0.113 | 0.010 | -0.177 | 0.098 | 0.130 | -0.042 | -0.192 |
| **PH** |  |  | 1.000 | 0.248* | 0.219 | 0.198 | -0.122 | -0.229* | -0.074 | 0.096 | 0.168 | 0.215 | 0.556** | 0.011 | 0.056 | 0.195 |
| **NBP** |  |  |  | 1.000 | 0.712** | 0.346** | -0.615** | -0.584** | -0.024 | 0.058 | 0.745** | 0.834** | 0.405** | -0.059 | -0.277* | 0.806** |
| **LA** |  |  |  |  | 1.000 | 0.497** | -0.650** | -0.503** | -0.045 | 0.133 | 0.679** | 0.831** | 0.306** | -0.144 | -0.178 | 0.822** |
| **LDW** |  |  |  |  |  | 1.000 | -0.073 | -0.086 | -0.060 | 0.052 | 0.422** | 0.339** | 0.143 | -0.077 | 0.053 | 0.233* |
| **TDM** |  |  |  |  |  |  | 1.000 | 0.788** | 0.002 | -0.143 | -0.646** | -0.672** | -0.203 | 0.114 | 0.351** | -0.867** |
| **PST** |  |  |  |  |  |  |  | 1.000 | 0.017 | 0.045 | -0.606** | -0.581** | -0.223 | 0.020 | 0.156 | -0.710** |
| **SPAD** |  |  |  |  |  |  |  |  | 1.000 | 0.286* | 0.005 | 0.002 | -0.127 | 0.055 | 0.077 | 0.001 |
| **CT** |  |  |  |  |  |  |  |  |  | 1.000 | 0.103 | 0.160 | 0.146 | 0.055 | 0.058 | 0.16 |
| **RWC** |  |  |  |  |  |  |  |  |  |  | 1.000 | 0.756** | 0.363** | 0.012 | -0.246* | 0.762** |
| **SWP** |  |  |  |  |  |  |  |  |  |  |  | 1.000 | 0.394** | -0.031 | -0.197 | 0.948** |
| **NCP** |  |  |  |  |  |  |  |  |  |  |  |  | 1.000 | 0.058 | -0.066 | 0.352** |
| **TW** |  |  |  |  |  |  |  |  |  |  |  |  |  | 1.000 | -0.181 | -0.081 |
| **CWP** |  |  |  |  |  |  |  |  |  |  |  |  |  |  | 1.000 | -0.277* |
| **HI** |  |  |  |  |  |  |  |  |  |  |  |  |  |  |  | 1.000 |
| **Year -2019** | | | | | | | | | | | | | | | | |
|  | **NDF** | **NDM** | **PH** | **NBP** | **LA** | **LDW** | **TDM** | **PST** | **SPAD** | **CT** | **RWC** | **SWP** | **NCP** | **TW** | **CWP** | **HI** |
| **NDF** | 1.000 | 0.946** | 0.085 | 0.322** | 0.304** | 0.294* | -0.095 | -0.233* | 0.031 | 0.046 | -0.019 | 0.205 | 0.341** | 0.103 | 0.423** | 0.188 |
| **NDM** |  | 1.000 | 0.127 | 0.339** | 0.333** | 0.328** | -0.146 | -0.261* | -0.076 | 0.029 | 0.031 | 0.246* | 0.379** | -0.007 | 0.368** | 0.241* |
| **PH** |  |  | 1.000 | 0.220 | 0.170 | 0.109 | -0.041 | -0.089 | -0.230* | -0.054 | -0.066 | 0.010 | 0.143 | 0.031 | 0.096 | 0.029 |
| **NBP** |  |  |  | 1.000 | 0.796** | 0.718** | -0.715** | -0.632** | -0.034 | -0.142 | 0.053 | 0.661** | 0.767** | -0.317** | 0.075 | 0.760** |
| **LA** |  |  |  |  | 1.000 | 0.916** | -0.729** | -0.650** | 0.024 | 0.109 | 0.107 | 0.728** | 0.760** | -0.343** | 0.114 | 0.842** |
| **LDW** |  |  |  |  |  | 1.000 | -0.706** | -0.602** | 0.000 | 0.129 | 0.076 | 0.674** | 0.656** | -0.384** | 0.103 | 0.803** |
| **TDM** |  |  |  |  |  |  | 1.000 | 0.826** | 0.074 | -0.045 | -0.200 | -0.534** | -0.579** | 0.444** | 0.170 | -0.846** |
| **PST** |  |  |  |  |  |  |  | 1.000 | 0.045 | -0.167 | -0.213 | -0.448** | -0.469** | 0.544** | 0.119 | -0.699** |
| **SPAD** |  |  |  |  |  |  |  |  | 1.000 | 0.077 | -0.046 | 0.035 | -0.024 | -0.004 | 0.222 | 0.003 |
| **CT** |  |  |  |  |  |  |  |  |  | 1.000 | 0.091 | -0.164 | -0.119 | -0.085 | -0.015 | -0.056 |
| **RWC** |  |  |  |  |  |  |  |  |  |  | 1.000 | 0.040 | 0.157 | -0.256* | -0.163 | 0.160 |
| **SWP** |  |  |  |  |  |  |  |  |  |  |  | 1.000 | 0.707** | -0.329** | 0.066 | 0.895** |
| **NCP** |  |  |  |  |  |  |  |  |  |  |  |  | 1.000 | -0.198 | 0.140 | 0.744** |
| **TW** |  |  |  |  |  |  |  |  |  |  |  |  |  | 1.000 | 0.114 | -0.435** |
| **CWP** |  |  |  |  |  |  |  |  |  |  |  |  |  |  | 1.000 | -0.016 |
| **HI** |  |  |  |  |  |  |  |  |  |  |  |  |  |  |  | 1.000 |

**Significance level**: **highly significant (p < 0.01), *significant (p > 0.01 or p < 0.05). NDF: Days to 50% flowering; NDM: Days to maturity; PH: Plant height; NBP: Number of branches plant^-1^; LA: Leaf area plant^-1^; LDW: Leaves dry weight plant^-1^; TDM: Total dry matter plant^-1^; PST: stem dry weight plant^-1^; SPAD: Chlorophyll content; CT: Canopy temperature; RWC: Relative water content; SWP: Seed weight/plant; NCP: Number of capsules; TW: Test weight; CWP: Capsules weightplant^-1^; HI: Harvest index plant^-1^

**Supplementary Table 5** Correlation analysis of 16 phenotypic traits during the year 2018 and 2019 under WS condition

| **Year -2018** | | | | | | | | | | | | | | | | |
| --- | --- | --- | --- | --- | --- | --- | --- | --- | --- | --- | --- | --- | --- | --- | --- | --- |
|  | **NDF** | **NDM** | **PH** | **NBP** | **LA** | **LDW** | **TDM** | **PST** | **SPAD** | **CT** | **RWC** | **SWP** | **NCP** | **TW** | **CWP** | **HI** |
| **NDF** | 1.000 | 0.624** | -0.050 | -0.042 | -0.119 | -0.216 | 0.130 | 0.039 | -0.039 | -0.030 | -0.226* | -0.179 | -0.064 | 0.106 | 0.039 | -0.165 |
| **NDM** |  | 1.000 | -0.053 | -0.086 | -0.111 | -0.150 | 0.180 | 0.070 | 0.039 | 0.051 | -0.248* | -0.189 | -0.115 | 0.131 | 0.111 | -0.188 |
| **PH** |  |  | 1.000 | 0.364** | 0.382** | 0.013 | -0.299** | -0.242* | -0.025 | -0.384** | 0.312** | 0.293* | 0.351** | -0.016 | -0.375** | 0.308** |
| **NBP** |  |  |  | 1.000 | 0.832** | 0.403** | -0.803** | -0.751** | 0.099 | -0.374** | 0.835** | 0.825** | 0.863** | 0.079 | -0.364** | 0.835** |
| **LA** |  |  |  |  | 1.000 | 0.476** | -0.872** | -0.760** | 0.043 | -0.371** | 0.890** | 0.926** | 0.939** | 0.112 | -0.288* | 0.934** |
| **LDW** |  |  |  |  |  | 1.000 | -0.473** | -0.415** | 0.100 | -0.110 | 0.424** | 0.451** | 0.455** | 0.132 | 0.016 | 0.465** |
| **TDM** |  |  |  |  |  |  | 1.000 | 0.807** | -0.089 | 0.305** | -0.906** | -0.900** | -0.897** | -0.131 | 0.312** | -0.938** |
| **PST** |  |  |  |  |  |  |  | 1.000 | -0.156 | 0.164 | -0.822** | -0.766** | -0.798** | -0.129 | 0.276* | -0.779** |
| **SPAD** |  |  |  |  |  |  |  |  | 1.000 | 0.124 | 0.061 | 0.060 | 0.110 | 0.031 | -0.121 | 0.068 |
| **CT** |  |  |  |  |  |  |  |  |  | 1.000 | -0.280* | -0.373** | -0.349** | 0.009 | 0.266* | -0.386** |
| **RWC** |  |  |  |  |  |  |  |  |  |  | 1.000 | 0.897** | 0.904** | 0.125 | -0.315** | 0.909** |
| **SWP** |  |  |  |  |  |  |  |  |  |  |  | 1.000 | 0.927** | 0.174 | -0.283* | 0.988** |
| **NCP** |  |  |  |  |  |  |  |  |  |  |  |  | 1.000 | 0.147 | -0.374** | 0.941** |
| **TW** |  |  |  |  |  |  |  |  |  |  |  |  |  | 1.000 | 0.095 | 0.147 |
| **CWP** |  |  |  |  |  |  |  |  |  |  |  |  |  |  | 1.000 | -0.301** |
| **HI** |  |  |  |  |  |  |  |  |  |  |  |  |  |  |  | 1.000 |
| **Year -2019** | | | | | | | | | | | | | | | | |

|  | **NDF** | **NDM** | **PH** | **NBP** | **LA** | **LDW** | **TDM** | **PST** | **SPAD** | **CT** | **RWC** | **SWP** | **NCP** | **TW** | **CWP** | **HI** |
| --- | --- | --- | --- | --- | --- | --- | --- | --- | --- | --- | --- | --- | --- | --- | --- | --- |
| **NDF** | 1.000 | 0.644** | -0.122 | -0.116 | -0.068 | -0.008 | 0.057 | 0.038 | -0.004 | 0.257* | -0.083 | -0.184 | 0.089 | 0.105 | 0.007 | -0.191 |
| **NDM** |  | 1.000 | -0.065 | -0.112 | 0.022 | 0.131 | -0.016 | 0.131 | -0.063 | 0.131 | -0.071 | -0.131 | 0.150 | 0.129 | -0.021 | -0.069 |
| **PH** |  |  | 1.000 | 0.074 | -0.111 | -0.010 | -0.060 | -0.041 | 0.004 | 0.105 | -0.046 | 0.027 | -0.262* | -0.095 | 0.005 | 0.083 |
| **NBP** |  |  |  | 1.000 | -0.069 | -0.197 | -0.070 | 0.074 | 0.106 | -0.013 | 0.065 | 0.238* | -0.001 | 0.041 | -0.053 | 0.233* |
| **LA** |  |  |  |  | 1.000 | 0.067 | 0.020 | 0.076 | -0.187 | 0.163 | 0.130 | -0.062 | -0.010 | 0.110 | 0.253* | -0.066 |
| **LDW** |  |  |  |  |  | 1.000 | 0.148 | -0.124 | -0.150 | -0.120 | -0.034 | 0.010 | -0.134 | -0.031 | 0.095 | -0.119 |
| **TDM** |  |  |  |  |  |  | 1.000 | -0.070 | 0.091 | 0.129 | -0.173 | -0.310** | 0.240* | 0.007 | -0.037 | -0.538** |
| **PST** |  |  |  |  |  |  |  | 1.000 | -0.149 | 0.086 | -0.037 | -0.115 | -0.052 | -0.079 | 0.238* | -0.010 |
| **SPAD** |  |  |  |  |  |  |  |  | 1.000 | -0.319** | 0.034 | 0.090 | 0.281* | 0.146 | -0.023 | -0.001 |
| **CT** |  |  |  |  |  |  |  |  |  | 1.000 | 0.052 | -0.118 | 0.105 | 0.031 | -0.037 | -0.211 |
| **RWC** |  |  |  |  |  |  |  |  |  |  | 1.000 | 0.120 | 0.058 | 0.009 | -0.041 | 0.219 |
| **SWP** |  |  |  |  |  |  |  |  |  |  |  | 1.000 | -0.078 | 0.076 | 0.052 | 0.615** |
| **NCP** |  |  |  |  |  |  |  |  |  |  |  |  | 1.000 | -0.000 | -0.165 | -0.278* |
| **TW** |  |  |  |  |  |  |  |  |  |  |  |  |  | 1.000 | -0.170 | 0.041 |
| **CWP** |  |  |  |  |  |  |  |  |  |  |  |  |  |  | 1.000 | 0.082 |
| **HI** |  |  |  |  |  |  |  |  |  |  |  |  |  |  |  | 1.000 |

**Significance level**: **highly significant (p < 0.01), *significant (p > 0.01 or p< 0.05). NDF: Days to 50% flowering; NDM: Days to maturity; PH: Plant height; NBP: Number of branches plant^-1^; LA: Leaf area plant^-1^; LDW: Leaves dry weight plant^-1^; TDM: Total dry matter plant^-1^; PST: stem dry weight plant^-1^; SPAD: Chlorophyll content; CT: Canopy temperature; RWC: Relative water content; SWP: Seed weight/plant; NCP: Number of capsules; TW: Test weight; CWP: Capsules weight plant^-1^; HI: Harvest index plant^-1^

**Supplementary Table 6** Correlation co-efficient analysis between seed yield and different morpho-physiological parameters among 76 sesame genotypes at genotypic and phenotypic levels under irrigated (WW) condition in year 2018

|  |  | **NDF** | **NDM** | **PH** | **NBP** | **LA** | **LDW** | **TDM** | **PST** | **SPAD** | **CT** | **RWC** | **NCP** | **TW** | **CWP** | **HI** | **SWP** |
| --- | --- | --- | --- | --- | --- | --- | --- | --- | --- | --- | --- | --- | --- | --- | --- | --- | --- |
| **NDF** | G | 1 | 0.246* | 0.268 | -0.010 | -0.045 | -0.112 | 0.062 | -0.074 | -0.231* | -0.486** | 0.007 | 0.326** | -0.061 | -0.102 | -0.010 | 0.008 |
|  | P | 1 | 0.272** | 0.286** | 0.121 | -0.027 | 0.043 | 0.113 | 0.0048 | -0.181* | -0.029 | 0.054 | 0.328** | 0.115 | 0.058 | -0.008 | 0.065 |
| **NDM** | G |  | 1 | 0.118 | -0.100 | -0.080 | 0.023 | 0.181 | 0.009 | -0.152 | -0.597** | 0.039 | 0.091 | -0.066 | -0.088 | -0.175 | -0.159 |
|  | P |  | 1 | 0.137 | 0.073 | -0.053 | 0.147 | 0.210** | 0.068 | -0.086 | -0.045 | 0.077 | 0.093 | 0.109 | 0.085 | -0.151 | -0.060 |
| **PH** | G |  |  | 1 | 0.230* | 0.216 | 0.208 | -0.125 | -0.275* | -0.095 | 0.109 | 0.156 | 0.562** | -0.057 | 0.006 | 0.223 | 0.245 |
|  | P |  |  | 1 | 0.243** | 0.215** | 0.223** | -0.094 | -0.219** | -0.077 | 0.138 | 0.174* | 0.559** | 0.025 | 0.074 | 0.215** | 0.256** |
| **NBP** | G |  |  |  | 1 | 0.787** | 0.227* | -0.805 | -0.778 | -0.097 | -0.376** | 0.800** | 0.414** | -0.406** | -0.658** | 0.924** | 0.855** |
|  | P |  |  |  | 1 | 0.673** | 0.439** | -0.492** | -0.444** | 0.012 | 0.282** | 0.719** | 0.372** | 0.085 | -0.110 | 0.712** | 0.811** |
| **LA** | G |  |  |  |  | 1 | 0.521** | -0.695** | -0.532** | -0.046 | 0.155 | 0.687** | 0.290* | -0.206 | -0.231* | 0.851** | 0.848** |
|  | P |  |  |  |  | 1 | 0.452** | -0.636** | -0.461** | -0.031 | 0.139 | 0.683** | 0.295** | -0.106 | -0.155 | 0.819** | 0.831** |
| **LDW** | G |  |  |  |  |  | 1 | -0.163 | -0.217 | -0.097 | -0.474** | 0.427** | 0.165 | -0.332** | -0.135 | 0.307 | 0.344** |
|  | P |  |  |  |  |  | 1 | -0.007 | -0.025 | 0.002 | 0.244** | 0.422** | 0.160* | 0.101 | 0.217** | 0.232** | 0.400** |
| **TDM** | G |  |  |  |  |  |  | 1 | 0.785** | -0.032 | -0.470** | -0.683** | -0.180 | 0.060 | 0.337** | -0887** | -0.745** |
|  | P |  |  |  |  |  |  | 1 | 0.781** | 0.026 | -0.004 | -0.595** | -0.154 | 0.177* | 0.379** | -0.829** | -0.610** |
| **PST** | G |  |  |  |  |  |  |  | 1 | -0.015 | -0.204 | -0.660** | -0.230* | -0.106 | 0.100 | -0.761** | -0.680** |
|  | P |  |  |  |  |  |  |  | 1 | 0.016 | 0.157 | -0.544** | -0.192* | 0.127 | 0.216** | -0.690** | -0.526** |
| **SPAD** | G |  |  |  |  |  |  |  |  | 1 | 0.391** | -0.005 | -0.153 | -0.061 | -0.011 | 0.048 | -0.093 |
|  | P |  |  |  |  |  |  |  |  | 1 | 0.317** | 0.022 | -0.139 | 0.044 | 0.074 | -0.041 | -0.054 |
| **CT** | G |  |  |  |  |  |  |  |  |  | 1 | 0.015 | 0.203 | -0.516** | -0.434** | 0.215 | -0.027 |
|  | P |  |  |  |  |  |  |  |  |  | 1 | 0.151 | 0.150 | 0.286** | 0.239** | 0.093 | 0.186* |
| **RWC** | G |  |  |  |  |  |  |  |  |  |  | 1 | 0.353** | -0.058 | -0.342** | 0.789** | 0.782** |
|  | P |  |  |  |  |  |  |  |  |  |  | 1 | 0.356** | 0.067 | -0.179* | 0.755** | 0.775** |
| **NCP** | G |  |  |  |  |  |  |  |  |  |  |  | 1 | 0.043 | -0.104 | 0.353** | 0.398** |
|  | P |  |  |  |  |  |  |  |  |  |  |  | 1 | 0.073 | -0.053 | 0.341** | 0.400** |
| **TW** | G |  |  |  |  |  |  |  |  |  |  |  |  | 1 | -0.543** | -0.122 | -0.176 |
|  | P |  |  |  |  |  |  |  |  |  |  |  |  | 1 | 0.012 | -0.080 | 0.017 |
| **CWP** | G |  |  |  |  |  |  |  |  |  |  |  |  |  | 1 | -0.304** | -0.287* |
|  | P |  |  |  |  |  |  |  |  |  |  |  |  |  | 1 | -0.229** | -0.089 |
| **HI** | G |  |  |  |  |  |  |  |  |  |  |  |  |  |  | 1 | 0.994** |
|  | P |  |  |  |  |  |  |  |  |  |  |  |  |  |  | 1 | 0.911** |
| **SWP** | G |  |  |  |  |  |  |  |  |  |  |  |  |  |  |  | 1 |
|  | P |  |  |  |  |  |  |  |  |  |  |  |  |  |  |  | 1 |

**Supplementary Table 7** Direct and indirect effects between seed yield and different morpho-physiological traits among 76 sesame genotypes at genotypic and phenotypic levels under irrigated (WW) conditions in year 2018.

|  |  | **NDF** | **NDM** | **PH** | **NBP** | **LA** | **LDW** | **TDM** | **PST** | **SPAD** | **CT** | **RWC** | **NCP** | **TW** | **CWP** | **HI** |
| --- | --- | --- | --- | --- | --- | --- | --- | --- | --- | --- | --- | --- | --- | --- | --- | --- |
| **NDF** | G | **0.02961** | 0.01925 | 0.00052 | 0.00069 | 0.01268 | -0.00898 | 0.04663 | -0.00798 | 0.00183 | -0.02523 | -0.00030 | -0.03191 | -0.00193 | -0.00149 | -0.02119 |
|  | P | **0.00628** | -0.0060 | -0.0009 | 0.02415 | -0.0040 | 0.00009 | 0.03816 | 0.00001 | 0.00460 | -0.00022 | 0.00412 | 0.00206 | 0.00251 | 0.00262 | -0.00744 |
| **NDM** | G | 0.00731 | **0.07801** | 0.00023 | 0.00676 | 0.02240 | 0.00184 | 0.13554 | 0.00104 | -0.0012 | -0.03099 | -0.00157 | -0.00896 | -0.00209 | -0.00129 | -0.36614 |
|  | P | 0.00141 | **-0.0221** | -0.0004 | 0.01460 | -0.0079 | 0.00030 | 0.07062 | 0.00013 | 0.00219 | -0.00034 | 0.00585 | 0.000059 | 0.00239 | 0.00381 | -0.13225 |
| **PH** | G | 0.00795 | 0.00922 | **0.00194** | -0.0156 | -0.0602 | 0.01627 | -0.0939 | -0.0294 | -0.0007 | 0.00567 | -0.00612 | -0.05503 | -0.00180 | 0.00009 | 0.46702 |
|  | P | 0.00180 | -0.0030 | **-0.0033** | 0.04854 | 0.03173 | 0.00046 | -0.0317 | -0.00042 | 0.00196 | 0.00103 | 0.01308 | 0.00350 | 0.00057 | 0.00332 | 0.18892 |
| **NBP** | G | -0.0003 | -0.0078 | 0.00045 | **-0.0676** | -0.2194 | 0.01820 | -0.6029 | -0.08319 | -0.0007 | -0.01954 | -0.03142 | -0.04055 | -0.01273 | -0.00956 | 1.93260 |
|  | P | 0.00076 | -0.0016 | -0.0008 | **0.19950** | 0.09912 | 0.00090 | -0.1650 | -0.00085 | -0.0003 | 0.00210 | 0.05406 | 0.00233 | 0.00186 | -0.00491 | 0.62430 |
| **LA** | G | -0.0013 | -0.0062 | 0.00042 | -0.0532 | **-0.2786** | 0.04175 | -0.5209 | -0.05694 | -0.0037 | 0.0084 | -0.02698 | -0.02840 | -0.00646 | -0.00335 | 1.78125 |
|  | P | -0.0001 | 0.00119 | -0.0007 | 0.13437 | **0.14710** | 0.00093 | -0.2130 | -0.00089 | 0.00081 | 0.00104 | 0.05136 | 0.00185 | -0.00233 | -0.00692 | 0.71732 |
| **LDW** | G | -0.0033 | 0.00180 | 0.00041 | -0.0153 | -0.1453 | **0.08005** | -0.1221 | -0.02327 | -0.0007 | -0.02462 | -0.01677 | -0.01321 | -0.01042 | -0.00196 | 0.64269 |
|  | P | 0.00027 | -0.0032 | -0.0007 | 0.08764 | 0.06662 | **0.00206** | -0.0025 | -0.00005 | -0.0000 | 0.00182 | 0.03176 | 0.00101 | 0.00223 | 0.00968 | 0.20363 |
| **TDM** | G | 0.00184 | 0.01412 | -0.0004 | 0.05444 | 0.19385 | -0.01306 | **0.74882** | 0.08393 | -0.0002 | -0.02438 | 0.02662 | 0.01765 | 0.00189 | 0.00489 | -1.85603 |
|  | P | 0.00072 | -0.0046 | 0.00031 | -0.0982 | -0.0935 | -0.00002 | **0.33499** | 0.00150 | -0.0000 | -0.00003 | -0.04478 | -0.00096 | 0.00386 | 0.01690 | -0.72643 |
| **PST** | G | -0.0022 | 0.00076 | -0.0005 | 0.05263 | 0.14841 | -0.01743 | 0.58804 | **0.10688** | -0.0001 | -0.01059 | 0.02593 | 0.2250 | -0.00333 | 0.00146 | -1.59288 |
|  | P | 0.00003 | -0.0015 | 0.00073 | -0.0886 | -0.0678 | -0.00005 | 0.26186 | **0.00192** | -0.0004 | 0.00118 | -0.04093 | -0.00120 | 0.00277 | 0.00965 | -0.06043 |
| **SPAD** | G | -0.0068 | -0.0118 | -0.0001 | 0.00657 | 0.01302 | -0.00779 | -0.0242 | -0.00165 | **0.00792** | 0.02028 | 0.00021 | 0.01499 | -0.00192 | -0.00017 | -0.10113 |
|  | P | -0.0011 | 0.00191 | 0.00025 | 0.00259 | -0.0046 | 0.00001 | 0.00013 | 0.00003 | **-0.0253** | 0.00236 | 0.00171 | -000087 | 0.00098 | 0.00332 | -0.03600 |
| **CT** | G | -0.0144 | -0.0466 | 0.00021 | 0.02548 | -0.0432 | -0.03800 | -0.0352 | -0.02183 | 0.00310 | **0.02186** | -0.00061 | -0.01985 | -0.01615 | -0.00631 | 0.45110 |
|  | P | -0.0001 | 0.00101 | -0.0004 | 0.05636 | 0.02055 | 0.00050 | -0.0015 | 0.00030 | -0.0080 | **0.00744** | 0.01141 | 0.00094 | 0.00625 | 0.01067 | 0.08172 |
| **RWC** | G | 0.00023 | 0.00311 | 0.00030 | -0.0541 | -0.1915 | 0.03419 | -0.5116 | -0.07062 | -0.0000 | 0.00081 | **-0.03925** | -0.03456 | -0.00184 | -0.00497 | 1.65218 |
|  | P | 0.00034 | -0.0017 | -0.0005 | 0.14346 | 0.10055 | 0.00087 | -0.1996 | -0.00105 | -0.0005 | 0.00113 | **0.07515** | 0.00223 | 0.00148 | -0.00800 | 0.66144 |
| **NCP** | G | 0.00966 | 0.00715 | 0.00109 | -0.0284 | -0.0809 | 0.01327 | -0.1351 | -0.02459 | -0.0012 | 0.01053 | -0.01387 | **-0.09777** | 0.00135 | -0.00152 | 0.73872 |
|  | P | 0.00207 | -0.0020 | -0.0018 | 0.07422 | 0.04351 | 0.00033 | -0.0516 | -0.00037 | 0.00355 | 0.00112 | 0.02677 | **0.00626** | 0.00159 | -0.00240 | 0.29893 |
| **TW** | G | -0.0018 | -0.0052 | -0.0001 | 0.02751 | 0.05757 | -0.02665 | 0.04526 | -0.01137 | -0.0004 | -0.02677 | 0.00230 | -0.00422 | **0.03129** | -0.00788 | -0.25612 |
|  | P | 0.00072 | -0.0024 | -0.0000 | 0.01699 | -0.0157 | 0.00021 | 0.05936 | 0.00024 | -0.0011 | 0.00213 | 0.00509 | 0.00046 | **0.02183** | 0.00057 | -0.07024 |
| **CWP** | G | -0.0030 | -0.0069 | 0.00001 | 0.04456 | 0.06445 | -0.01082 | 0.02524 | 0.01076 | -0.0000 | -0.02255 | 0.01346 | 0.01022 | -0.01699 | **0.01451** | -0.63753 |
|  | P | 0.00037 | -0.0018 | -0.0002 | -0.0219 | -0.0228 | 0.00045 | 0.12713 | 0.00042 | -0.0018 | 0.00178 | -0.01350 | 0.00034 | 0.00028 | **0.04454** | -0.20118 |
| **HI** | G | -0.0003 | -0.0136 | 0.00043 | -0.0624 | -0.2373 | 0.02460 | -0.6644 | -0.08140 | -0.0003 | 0.01118 | -0.03100 | -0.03453 | -0.00383 | -0.00442 | **2.09163** |
|  | P | -0.0000 | 0.00334 | -0.0007 | 0.14215 | 0.12048 | 0.00048 | -0.2778 | -0.00133 | 0.00104 | 0.00069 | 0.05675 | 0.00214 | -0.00175 | -0.01023 | **0.87585** |

***Residual**: 0.0617

**Supplementary Table 8** Correlation co-efficient analysis between seed yield and different morpho-physiological parameters among 76 sesame genotypes at genotypic and phenotypic levels under stress (WS) conditions in year 2018.

|  |  | **NDF** | **NDM** | **PH** | **NBP** | **LA** | **LDW** | **TDM** | **PST** | **SPAD** | **CT** | **RWC** | **NCP** | **TW** | **CWP** | **HI** | **SWP** |
| --- | --- | --- | --- | --- | --- | --- | --- | --- | --- | --- | --- | --- | --- | --- | --- | --- | --- |
| **NDF** | G | 1 | 0.595** | -0.110 | -0.188 | -0.132 | -0.318** | 0.046 | -0.01 | -0.070 | -0.262* | -0.213 | -0.068 | 0.024 | -0.004 | -0.116 | -0.127 |
|  | P | 1 | 0.613** | -0.084 | 0.026 | -0.101 | -0.122 | 0.090 | 0.057 | -0.009 | 0.093 | -0.161* | -0.047 | 0.180* | 0.080 | -0.086 | -0.097 |
| **NDM** | G |  | 1 | -0.026 | -0.201 | -0.139 | -0.277* | 0.160 | 0.047 | -0.029 | -0.126 | -0.272* | -0.154 | 0.004 | 0.036 | -0.171 | -0.164 |
|  | P |  | 1 | 0.003 | -0.012 | -0.093 | -0.068 | 0.192* | 0.094 | 0.037 | 0.122 | -0.204* | -0.114 | 0.154 | 0.115 | -0.127 | -0.120 |
| **PH** | G |  |  | 1 | 0.427** | 0.354** | -0.012 | -0.285* | -0.238* | -0.061 | -0.749** | 0.258* | 0.311** | -0.075 | -0.406** | 0.331** | 0.344** |
|  | P |  |  | 1 | 0.313** | 0.352** | 0.0266 | -0.253** | -0.202* | -0.038 | -0.264** | 0.267** | 0.316** | -0.026 | -0.352** | 0.328** | 0.317** |
| **NBP** | G |  |  |  | 1 | 1.064** | 0.352** | -1.145** | -1.106** | 0.061 | -1.498** | 1.017** | 1.081** | -0.164 | -0.597** | 1.104** | 1.181** |
|  | P |  |  |  | 1 | 0.710** | 0.449** | -0.622** | -0.552** | 0.099 | -0.042 | 0.714** | 0.728** | 0.194* | -0.206* | 0.735** | 0.683** |
| **LA** | G |  |  |  |  | 1 | 0.499** | -0.916** | -0.798** | 0.036 | -0.651** | 0.892** | 0.943** | 0.116 | -0.310** | 0.959** | 0.995** |
|  | P |  |  |  |  | 1 | 0.453** | -0.868** | -0.746** | 0.038 | -0.262** | 0.879** | 0.938** | 0.122 | -0.268** | 0.957** | 0.925** |
| **LDW** | G |  |  |  |  |  | 1 | -0.603** | -0.531** | 0.066 | -0.701** | 0.449** | 0.484** | -0.022 | -0.094 | 0.500** | 0.532** |
|  | P |  |  |  |  |  | 1 | -0.419** | -0.317** | 0.155 | 0.112 | 0.440** | 0.447** | 0.250** | 0.079 | 0.459** | 0.414** |
| **TDM** | G |  |  |  |  |  |  | 1 | 0.866** | -0.129 | 0.347** | -0.955** | -0.943** | -0.259* | 0.271* | -0.968** | -0.976** |
|  | P |  |  |  |  |  |  | 1 | 0.832** | -0.091 | 0.295** | -0.893** | -0.899** | -0.116 | 0.318** | -0.919** | -0.865** |
| **PST** | G |  |  |  |  |  |  |  | 1 | -0.174 | 0.064 | -0.867** | -0.838** | -0.288** | 0.243* | -0.826** | -0.848** |
|  | P |  |  |  |  |  |  |  | 1 | -0.103 | 0.245** | -0.777** | -0.773** | -0.066 | 0.302** | -0.772** | -0.738** |
| **SPAD** | G |  |  |  |  |  |  |  |  | 1 | 0.020 | 0.048 | 0.105 | 0.014 | -0.129 | 0.073 | 0.086 |
|  | P |  |  |  |  |  |  |  |  | 1 | 0.189* | 0.081 | 0.114 | 0.111 | -0.064 | 0.079 | 0.066 |
| **CT** | G |  |  |  |  |  |  |  |  |  | 1 | -0.559** | -0.608** | -0.408** | 0.183 | -0.726** | -0.675** |
|  | P |  |  |  |  |  |  |  |  |  | 1 | -0.133 | -0.220** | 0.277** | 0.320** | -0.278** | -0.276** |
| **RWC** | G |  |  |  |  |  |  |  |  |  |  | 1 | 0.910** | 0.161 | -0.313** | 0.925** | 0.959** |
|  | P |  |  |  |  |  |  |  |  |  |  | 1 | 0.905** | 0.184* | -0.260** | 0.914** | 0.877** |
| **NCP** | G |  |  |  |  |  |  |  |  |  |  |  | 1 | 0.173 | -0.382** | 0.962** | 1.001** |
|  | P |  |  |  |  |  |  |  |  |  |  |  | 1 | 0.173** | -0.336** | 0.957** | 0.927** |
| **TW** | G |  |  |  |  |  |  |  |  |  |  |  |  | 1 | -0.041 | 0.128 | 0.160 |
|  | P |  |  |  |  |  |  |  |  |  |  |  |  | 1 | 0.154 | 0.136 | 0.109 |
| **CWP** | G |  |  |  |  |  |  |  |  |  |  |  |  |  | 1 | -0.329* | -0.332** |
|  | P |  |  |  |  |  |  |  |  |  |  |  |  |  | 1 | -0.285** | -0.271** |
| **HI** | G |  |  |  |  |  |  |  |  |  |  |  |  |  |  | 1 | 1.026** |
|  | P |  |  |  |  |  |  |  |  |  |  |  |  |  |  | 1 | 0.955** |
| **SWP** | G |  |  |  |  |  |  |  |  |  |  |  |  |  |  |  | 1 |
|  | P |  |  |  |  |  |  |  |  |  |  |  |  |  |  |  | 1 |

**Supplementary Table 9** Direct and indirect effects between seed yield and different morpho-physiological traits among 76 sesame genotypes at genotypic and phenotypic levels under stress (WS) conditions in year 2018.

|  |  | **NDF** | **NDM** | **PH** | **NBP** | **LA** | **LDW** | **TDM** | **PST** | **SPAD** | **CT** | **RWC** | **NCP** | **TW** | **CWP** | **HI** |
| --- | --- | --- | --- | --- | --- | --- | --- | --- | --- | --- | --- | --- | --- | --- | --- | --- |
| **NDF** | G | **-0.0421** | 0.0116 | 0.0018 | 0.0086 | -0.0093 | 0.0043 | 0.0052 | 0.0007 | -0.0002 | 0.0083 | -0.0075 | -0.0138 | -0.0002 | -0.00002 | -0.0948 |
|  | P | **-0.0176** | 0.0067 | 0.0003 | -0.0015 | -0.0046 | 0.0029 | 0.01394 | -0.0008 | 0.00003 | -0.0017 | -0.0104 | -0.0103 | -0.0038 | 0.00177 | -0.0720 |
| **NDM** | G | -0.0251 | **0.0195** | 0.0004 | 0.0092 | -0.0098 | 0.0037 | 0.0178 | -0.0037 | -0.00009 | 0.0040 | -0.0096 | -0.0313 | -0.00004 | 0.00020 | -0.1394 |
|  | P | -0.0108 | **0.0109** | -0.0001 | 0.0007 | -0.0042 | 0.0016 | 0.02956 | -0.0013 | -0.00011 | -0.0022 | -0.0132 | -0.02483 | -0.00326 | 0.00254 | -0.1059 |
| **PH** | G | 0.0046 | -0.0005 | **-0.0162** | -0.0196 | 0.0250 | 0.0001 | -0.0317 | 0.0188 | -0.00020 | 0.0237 | 0.00917 | 0.0633 | 0.00065 | -0.00224 | 0.2693 |
|  | P | 0.0014 | 0.00004 | **-0.0035** | -0.0182 | 0.0161 | -0.0006 | -0.0389 | 0.00283 | 0.00012 | 0.00489 | 0.01732 | 0.06881 | 0.00055 | -0.00773 | 0.2742 |
| **NBP** | G | 0.0079 | -0.0039 | -0.0069 | **-0.0458** | 0.0751 | -0.0047 | -0.1273 | 0.0875 | 0.00020 | 0.0473 | 0.03608 | 0.2197 | 0.00141 | -0.00330 | 0.8981 |
|  | P | -0.0004 | -0.0001 | -0.0011 | **-0.0580** | 0.0324 | -0.0106 | -0.0956 | 0.0077 | -0.00030 | 0.00079 | 0.04628 | 0.15828 | -0.00411 | -0.00455 | 0.6130 |
| **LA** | G | 0.0055 | -0.0027 | -0.0057 | -0.0488 | **0.07053** | -0.0067 | -0.1019 | 0.0632 | 0.00012 | 0.0205 | 0.03165 | 0.1916 | -0.0010 | -0.00171 | 0.7808 |
|  | P | 0.0018 | -0.0010 | -0.0012 | -0.0411 | **0.0456** | -0.0107 | -0.1335 | 0.01043 | -0.00011 | 0.00485 | 0.05699 | 0.20381 | -0.00260 | -0.00589 | 0.7986 |
| **LDW** | G | 0.01342 | -0.0054 | 0.00020 | -0.0161 | 0.03522 | **-0.0135** | -0.0670 | 0.0420 | 0.00022 | 0.02218 | 0.01595 | 0.09841 | 0.00019 | -0.00052 | 0.40715 |
|  | P | 0.0021 | -0.0007 | -0.0009 | -0.0261 | 0.0207 | **-0.0235** | -0.0644 | 0.0044 | -0.00047 | -0.0020 | 0.02851 | 0.09712 | -0.00530 | 0.00174 | 0.3826 |
| **TDM** | G | -0.00197 | 0.00314 | 0.00464 | 0.0525 | -0.0646 | 0.00817 | **0.1111** | -0.0686 | -0.00042 | -0.0109 | -0.03388 | -0.1917 | 0.00221 | 0.00150 | -0.78744 |
|  | P | -0.0016 | 0.0021 | 0.0009 | 0.0361 | -0.0396 | 0.0098 | **0.15374** | -0.0116 | 0.00028 | -0.0054 | -0.05789 | -0.1954 | 0.00247 | 0.00698 | -0.76666 |
| **PST** | G | 0.00042 | 0.00093 | 0.00388 | 0.05071 | -0.0563 | 0.0072 | 0.0963 | **-0.0791** | -0.00056 | -0.0020 | -0.0307 | -0.1704 | 0.00246 | 0.00134 | -0.6721 |
|  | P | -0.0010 | 0.0010 | 0.0007 | 0.0320 | -0.0341 | 0.0074 | 0.12807 | **-0.0139** | 0.00031 | -0.0045 | -0.05038 | -0.1679 | 0.00142 | 0.00663 | -0.64401 |
| **SPAD** | G | 0.00296 | -0.0005 | 0.00100 | -0.0028 | 0.00254 | -0.0009 | -0.0143 | 0.01382 | **0.00323** | -0.0006 | 0.00173 | 0.02148 | -0.00013 | -0.00072 | 0.05996 |
|  | P | 0.0001 | 0.0004 | 0.0001 | -0.0057 | 0.0017 | -0.0036 | -0.0140 | 0.00144 | **-0.00302** | -0.0035 | 0.00527 | 0.02491 | -0.00235 | -0.00140 | 0.06603 |
| **CT** | G | 0.01106 | -0.0024 | 0.0122 | 0.0686 | -0.0459 | 0.00950 | 0.03860 | -0.0050 | 0.00007 | **-0.0316** | -0.0198 | -0.1237 | 0.00348 | 0.00101 | -0.59116 |
|  | P | -0.0016 | 0.0013 | 0.0009 | 0.00247 | -0.0119 | -0.0026 | 0.04543 | -0.0034 | -0.00057 | **-0.0184** | -0.00862 | -0.04798 | -0.00589 | 0.00703 | -0.23254 |
| **RWC** | G | 0.0089 | -0.0053 | -0.0042 | -0.0466 | 0.0629 | -0.0060 | -0.1062 | 0.0686 | 0.00016 | 0.01768 | **0.03545** | 0.1850 | -0.00138 | -0.00173 | 0.75253 |
|  | P | 0.0028 | -0.0022 | -0.0009 | -0.0414 | 0.0401 | -0.0103 | -0.1373 | 0.01086 | -0.00025 | 0.00246 | **0.06478** | 0.19658 | -0.00392 | -0.00571 | 0.76224 |
| **NCP** | G | 0.00286 | -0.0030 | -0.0050 | -0.0495 | 0.0665 | -0.0065 | -0.1049 | 0.0663 | 0.00034 | 0.0192 | 0.0322 | **0.20319** | -0.00149 | -0.00211 | 0.78310 |
|  | P | 0.0008 | -0.0012 | -0.0011 | -0.0422 | 0.0428 | -0.0105 | -0.1383 | 0.01080 | -0.00035 | 0.00409 | 0.05863 | **0.21722** | -0.00369 | -0.00739 | 0.79834 |
| **TW** | G | -0.00104 | 0.00009 | 0.00123 | 0.00756 | 0.00825 | 0.00030 | -0.0288 | 0.0228 | 0.00005 | 0.01290 | 0.0057 | 0.03534 | **-0.00854** | -0.00023 | 0.10482 |
|  | P | -0.0032 | 0.0016 | 0.00009 | -0.0112 | 0.0056 | -0.0058 | -0.0179 | 0.00093 | -0.00034 | -0.0051 | 0.01198 | 0.03777 | **-0.02123** | 0.00340 | 0.11348 |
| **CWP** | G | 0.00018 | 0.00072 | 0.00661 | 0.02740 | -0.02191 | 0.00128 | 0.03016 | -0.0192 | -0.00042 | -0.0057 | -0.01112 | -0.0776 | 0.00035 | **0.00552** | -0.26810 |
|  | P | -0.0014 | 0.0012 | 0.0012 | 0.0120 | -0.0122 | -0.0018 | 0.04892 | -0.0042 | 0.00019 | -0.0059 | -0.01684 | -0.07318 | -0.00329 | **0.02195** | -0.23838 |
| **HI** | G | 0.00491 | -0.0033 | -0.0053 | -0.0506 | 0.0677 | -0.0067 | -0.1076 | 0.0654 | 0.00024 | 0.02298 | 0.03279 | 0.1955 | -0.0011 | -0.00182 | **0.81351** |
|  | P | 0.0015 | -0.0013 | -0.0011 | -0.0426 | 0.0437 | -0.0108 | -0.1413 | 0.01079 | -0.00024 | 0.00516 | 0.05922 | 0.20798 | -0.00289 | -0.00628 | **0.83377** |

***Residual:** 0.0791

**Supplementary Table 10** Correlation co-efficient analysis between seed yield and different morpho-physiological parameters among 76 sesame genotypes at genotypic and phenotypic levels under irrigated (WW) conditions in year 2019.

|  |  | **NDF** | **NDM** | **PH** | **NBP** | **LA** | **LDW** | **TDM** | **PST** | **SPAD** | **CT** | **RWC** | **NCP** | **TW** | **CWP** | **HI** | **SWP** |
| --- | --- | --- | --- | --- | --- | --- | --- | --- | --- | --- | --- | --- | --- | --- | --- | --- | --- |
| **NDF** | G | 1 | 0.963 | 0.013 | 0.244* | 0.320** | 0.279** | -0.174 | 0.362** | -0.038 | NaN | -0.058 | 0.365** | -0.137 | 0.328** | 0.160 | 0.213 |
|  | P | 1 | 0.9213** | 0.0701 | 0.3599** | 0.3381** | 0.3387** | 0.0237 | -0.137 | 0.0267 | 0.2997** | 0.0246 | 0.3314** | 0.1919* | 0.497** | 0.2306** | 0.3006** |
| **NDM** | G |  | 1 | 0.0597 | 0.308** | 0.342** | 0.337** | -0.182 | -0.321** | -0.131 | NaN | -0.003 | 0.386** | -0.153 | 0.296** | 0.232* | 0.269* |
|  | P |  | 1 | 0.0905 | 0.3749** | 0.3592** | 0.368** | 0.0579 | -0.1863* | -0.0831 | 0.2218** | 0.0417 | 0.3785** | 0.0724 | 0.3815** | 0.2657** | 0.3106** |
| **PH** | G |  |  | 1 | 0.179 | 0.158 | 0.107 | -0.083 | -0.161 | -0.237* | NaN | -0.050 | 0.112 | -0.020 | 0.0223 | 0.0238 | -0.016 |
|  | P |  |  | 1 | 0.2105** | 0.1588 | 0.1142 | -0.0423 | -0.0979 | -0.2058* | 0.0119 | -0.0334 | 0.1213 | 0.0634 | 0.1028 | 0.0585 | 0.0288 |
| **NBP** | G |  |  |  | 1 | 0.829** | 0.751** | -0.849** | -0.789** | -0.0891 | NaN | 0.0409 | 0.788** | -0.648** | -0.068 | 0.789** | 0.691** |
|  | P |  |  |  | 1 | 0.7916** | 0.738** | -0.616** | -0.5591** | -0.0314 | 0.1231 | 0.093 | 0.745** | -0.1906* | 0.1321 | 0.7778** | 0.6871** |
| **LA** | G |  |  |  |  | 1 | 0.916 | -0.749 | -0.680 | 0.017 | NaN | 0.077 | 0.786** | -0.463** | 0.117 | 0.848** | 0.775** |
|  | P |  |  |  |  | 1 | 0.9086** | -0.6252** | -0.5865** | 0.0202 | 0.1394 | 0.0925 | 0.7753** | -0.265** | 0.1676* | 0.8324** | 0.76** |
| **LDW** | G |  |  |  |  |  | 1 | -0.766** | -0.650** | -0.003 | NaN | 0.043 | 0.688** | -0.558** | 0.073 | 0.823** | 0.714** |
|  | P |  |  |  |  |  | 1 | -0.6127* | -0.5268** | 0.0205 | 0.1832* | 0.077 | 0.6718** | -0.2609** | 0.1622** | 0.8166** | 0.7085** |
| **TDM** | G |  |  |  |  |  |  | 1 | 0.823** | 0.054 | NaN | -0.201 | -0.617** | 0.425** | 0.072 | -0.930** | -0.646** |
|  | P |  |  |  |  |  |  | 1 | 0.8266** | 0.0742 | 0.1875* | 0.1353 | -0.5537** | 0.4611** | 0.2377** | -0.7796** | -0.46** |
| **PST** | G |  |  |  |  |  |  |  | 1 | -0.003 | NaN | -0.217 | -0.526** | 0.528** | -0.0172 | -0.782** | -0.554** |
|  | P |  |  |  |  |  |  |  | 1 | 0.0351 | 0.0961 | -0.1529 | -0.467** | 0.5354** | 0.1595* | -0.6478** | -0.3916** |
| **SPAD** | G |  |  |  |  |  |  |  |  | 1 | NaN | -0.043 | -0.057 | -0.119 | 0.189 | -0.023 | 0.003 |
|  | P |  |  |  |  |  |  |  |  | 1 | 0.1084 | -0.0215 | -0.0477 | 0.0194 | 0.2055* | 0.0116 | 0.0189 |
| **CT** | G |  |  |  |  |  |  |  |  |  | 1 | NaN | NaN | NaN | NaN | NaN | NaN |
|  | P |  |  |  |  |  |  |  |  |  | 1 | 0.0848 | -0.0154 | 0.2946** | 0.2956** | 0.0544 | 0.0687 |
| **RWC** | G |  |  |  |  |  |  |  |  |  |  | 1 | 0.180 | -0.375** | -0.189 | 0.138 | 0.042 |
|  | P |  |  |  |  |  |  |  |  |  |  | 1 | 0.1843* | -0.1685* | -0.0802 | 0.1601* | 0.0706 |
| **NCP** | G |  |  |  |  |  |  |  |  |  |  |  | 1 | -0.328** | 0.126 | 0.778** | 0.769** |
|  | P |  |  |  |  |  |  |  |  |  |  |  | 1 | -0.1989* | 0.1281 | 0.7659** | 0.7377** |
| **TW** | G |  |  |  |  |  |  |  |  |  |  |  |  | 1 | -0.164 | -0.631** | -0.521** |
|  | P |  |  |  |  |  |  |  |  |  |  |  |  | 1 | 0.21** | -0.3169** | 0.225** |
| **CWP** | G |  |  |  |  |  |  |  |  |  |  |  |  |  | 1 | -0.098 | -0.041 |
|  | P |  |  |  |  |  |  |  |  |  |  |  |  |  | 1 | 0.0351 | 0.1369 |
| **HI** | G |  |  |  |  |  |  |  |  |  |  |  |  |  |  | 1 | 0.904** |
|  | P |  |  |  |  |  |  |  |  |  |  |  |  |  |  | 1 | 0.8878** |
| **SWP** | G |  |  |  |  |  |  |  |  |  |  |  |  |  |  |  | 1 |
|  | P |  |  |  |  |  |  |  |  |  |  |  |  |  |  |  | 1 |

*NaN indicates negative genotypic variance for one or more traits

**Supplementary Table 11** Direct and indirect effects between seed yield and different morpho-physiological traits among 76 sesame genotypes at genotypic and phenotypic levels under irrigated (WW) conditions in year 2019

|  |  | **NDF** | **NDM** | **PH** | **NBP** | **LA** | **LDW** | **TDM** | **PST** | **SPAD** | **CT** | **RWC** | **NCP** | **TW** | **CWP** | **HI** |
| --- | --- | --- | --- | --- | --- | --- | --- | --- | --- | --- | --- | --- | --- | --- | --- | --- |
| **NDF** | G | **0.0580** | -0.0184 | -0.0004 | -0.0143 | 0.0673 | -0.0447 | -0.1239 | 0.0103 | 0.0005 | 0.0435 | 0.0051 | 0.0150 | 0.0093 | -0.0248 | 0.2308 |
|  | P | **0.0539** | -0.0289 | -0.0025 | 0.0066 | 0.0689 | -0.0699 | 0.0164 | -0.0034 | -0.0010 | -0.0266 | -0.0019 | 0.0098 | -0.0166 | -0.0264 | 0.3223 |
| **NDM** | G | 0.0559 | **-0.0191** | -0.0022 | -0.0181 | 0.0718 | -0.0540 | -0.1298 | 0.0091 | 0.0019 | 0.0154 | 0.0003 | 0.0158 | 0.0105 | -0.0225 | 0.3346 |
|  | P | 0.0497 | **-0.0314** | -0.0033 | 0.0069 | 0.0732 | -0.0760 | -0.0402 | -0.0046 | 0.0032 | -0.0197 | -0.0032 | 0.0112 | -0.0062 | -0.0203 | 0.3714 |
| **PH** | G | 0.0007 | -0.0011 | **-0.0376** | -0.0105 | 0.0332 | -0.0171 | -0.0589 | 0.0045 | 0.0035 | 0.0240 | 0.0044 | 0.0046 | 0.0014 | -0.0017 | 0.0342 |
|  | P | 0.0037 | -0.0028 | **-0.0369** | 0.0038 | 0.0323 | -0.0235 | -0.0294 | -0.0024 | 0.0079 | -0.0010 | 0.0026 | 0.0036 | -0.0054 | -0.0054 | 0.0817 |
| **NBP** | G | 0.0142 | -0.0059 | -0.0067 | **-0.0587** | 0.1473 | -0.1202 | -0.6032 | 0.0224 | 0.0013 | 0.0606 | -0.0035 | 0.0324 | 0.0444 | 0.0051 | 1.1348 |
|  | P | 0.0194 | -0.0117 | -0.0077 | **0.0184** | 0.1614 | -0.1525 | -0.4282 | -0.0138 | 0.0012 | -0.0109 | -0.0073 | 0.0222 | 0.0164 | -0.00703 | 1.0873 |
| **LA** | G | 0.0185 | -0.0065 | -0.0059 | -0.0487 | **0.2101** | -0.1467 | -0.5322 | 0.0193 | -0.0002 | -0.0008 | -0.0067 | 0.0323 | 0.0317 | -0.0089 | 1.2204 |
|  | P | 0.0182 | -0.0112 | -0.0058 | 0.0146 | **0.2039** | -0.1878 | -0.4345 | -0.0145 | -0.0007 | -0.0123 | -0.0073 | 0.0231 | 0.0229 | -0.0089 | 1.1636 |
| **LDW** | G | 0.0162 | -0.0064 | -0.0040 | -0.0441 | 0.1926 | **-0.1600** | -0.5443 | 0.0185 | 0.00005 | 0.0053 | -0.0038 | 0.0282 | 0.0381 | -0.0055 | 1.1836 |
|  | P | 0.0182 | -0.0115 | -0.0042 | 0.0136 | 0.1853 | **-0.2066** | -0.4258 | -0.1310 | -0.0007 | -0.0162 | -0.0060 | 0.0200 | 0.0225 | -0.0086 | 1.1417 |
| **TDM** | G | -0.0101 | 0.0035 | 0.0031 | 0.0498 | -0.1575 | 0.1227 | **0.7100** | -0.0234 | -0.0008 | 0.0370 | 0.0176 | -0.0253 | -0.0291 | -0.0054 | -1.3385 |
|  | P | 0.0012 | 0.0018 | 0.0015 | -0.0113 | -0.1275 | 0.1266 | **0.6950** | 0.0205 | -0.0028 | -0.0166 | 0.0106 | -0.0165 | -0.0399 | -0.0126 | -1.0898 |
| **PST** | G | -0.0210 | 0.0061 | 0.0060 | 0.0463 | -0.1429 | 0.1041 | 0.5845 | **-0.0284** | 0.00006 | 0.0531 | 0.0190 | -0.0216 | -0.0361 | 0.0013 | -1.1254 |
|  | P | -0.0073 | 0.0058 | 0.0036 | -0.0103 | -0.1196 | 0.1089 | 0.5745 | **0.0248** | -0.0013 | -0.0085 | 0.0120 | -0.0139 | -0.0464 | -0.0085 | -0.9054 |
| **SPAD** | G | -0.0022 | 0.0025 | 0.0089 | 0.0052 | 0.0036 | 0.0006 | 0.0386 | 0.0001 | **-0.0147** | -0.0004 | 0.0038 | -0.0023 | 0.0081 | -0.0143 | -0.0338 |
|  | P | 0.0014 | 0.0026 | 0.0076 | -0.0005 | 0.0041 | -0.0042 | 0.0515 | 0.0008 | **-0.0387** | -0.0096 | 0.0017 | -0.0014 | -0.0016 | -0.0109 | 0.0162 |
| **CT** | G | -0.0401 | 0.0047 | 0.0143 | 0.0566 | 0.0029 | 0.0137 | -0.4190 | 0.0241 | -0.0001 | **0.0628** | 0.0212 | -0.0101 | 0.1187 | 0.0785 | -0.6864 |
|  | P | 0.0151 | -0.0065 | -0.0004 | 0.0021 | 0.0265 | -0.0353 | 0.1217 | 0.0022 | -0.0039 | **-0.0830** | -0.0062 | -0.0004 | -0.0238 | -0.0147 | 0.0710 |
| **RWC** | G | -0.0034 | 0.00007 | 0.0019 | -0.0024 | 0.0162 | -0.0070 | -0.1432 | 0.0062 | 0.0006 | 0.0152 | **-0.087** | 0.0074 | 0.0256 | 0.0143 | 0.1984 |
|  | P | 0.0013 | -0.0013 | 0.0012 | 0.0017 | 0.0188 | -0.0159 | -0.0940 | -0.0038 | 0.0008 | -0.0075 | **-0.0789** | 0.0054 | 0.0146 | 0.0042 | 0.2238 |
| **NCP** | G | 0.0211 | -0.0073 | -0.0042 | -0.0462 | 0.1653 | -0.1101 | -0.4383 | 0.0149 | 0.0008 | 0.0155 | -0.0158 | **0.0411** | 0.0225 | -0.0096 | 1.1194 |
|  | P | 0.0178 | -0.0119 | -0.0044 | 0.0137 | 0.1581 | -0.1388 | -0.3848 | -0.0116 | 0.00185 | 0.0013 | -0.0145 | **0.0298** | 0.0172 | -0.0068 | 1.0707 |
| **TW** | G | -0.0079 | 0.0029 | 0.0007 | 0.0381 | -0.0974 | 0.0893 | 0.3021 | -0.0150 | 0.0017 | 0.1090 | 0.0329 | -0.0135 | **-0.0684** | 0.0125 | -0.9083 |
|  | P | 0.0103 | -0.0022 | -0.0023 | -0.0035 | -0.0540 | 0.0538 | 0.3204 | 0.0133 | -0.0007 | -0.0262 | 0.0133 | -0.0059 | **-0.0868** | -0.0112 | -0.4431 |
| **CWP** | G | 0.0190 | -0.0056 | -0.0008 | 0.0040 | 0.0246 | -0.0117 | 0.0512 | 0.0004 | -0.0027 | 0.0650 | 0.0165 | 0.0052 | 0.0112 | **-0.0758** | -0.1417 |
|  | P | 0.0268 | -0.0120 | -0.0038 | 0.0024 | 0.0341 | -0.0334 | 0.1652 | 0.0039 | -0.0079 | -0.0263 | 0.0063 | 0.0038 | -0.0182 | **-0.0532** | 0.0490 |
| **HI** | G | 0.0093 | -0.0044 | -0.0009 | -0.0463 | 0.1783 | -0.1317 | -0.6608 | 0.0222 | 0.0003 | 0.0299 | -0.0120 | 0.0319 | 0.0432 | 0.0074 | **1.4381** |
|  | P | 0.0124 | -0.0083 | -0.0021 | 0.0143 | 0.1698 | -0.1688 | -0.5418 | -0.0161 | -0.0004 | -0.0048 | -0.0126 | 0.0228 | 0.0274 | -0.0018 | **1.3979** |

***Residual**: 0.0392

**Supplementary Table 12** Correlation co-efficient analysis between seed yield and different morpho-physiological parameters among 76 sesame genotypes at genotypic and phenotypic levels under stress (WS) conditions in year 2019.

|  |  | **NDF** | **NDM** | **PH** | **NBP** | **LA** | **LDW** | **TDM** | **PST** | **SPAD** | **CT** | **RWC** | **NCP** | **TW** | **CWP** | **HI** | **SWP** |
| --- | --- | --- | --- | --- | --- | --- | --- | --- | --- | --- | --- | --- | --- | --- | --- | --- | --- |
| **NDF** | G | 1 | 0.6292** | -0.174 | -0.203 | -0.138 | -0.080 | -0.038 | 0.0174 | -0.045 | 0.015 | -0.093 | 0.047 | -0.112 | -0.052 | -0.202 | -0.2858 |
|  | P | 1 | 0.659** | -0.078 | -0.041 | -0.018 | 0.139 | 0.091 | 0.060 | 0.018 | 0.369** | -0.040 | 0.097 | 0.183* | 0.066 | -0.122 | -0.117 |
| **NDM** | G |  | 1 | -0.084 | -0.182 | 0.0014 | 0.0337 | -0.1119 | 0.1076 | -0.0729 | -0.045 | -0.080 | 0.124 | 0.045 | -0.084 | -0.094 | -0.2417* |
|  | P |  | 1 | -0.004 | -0.030 | 0.074 | 0.195* | 0.031 | 0.140 | -0.116 | 0.265** | -0.037 | 0.178 | 0.172* | 0.017 | -0.055 | -0.087 |
| **PH** | G |  |  | 1 | 0.060 | -0.118 | -0.076 | -0.094 | -0.052 | 0.007 | 0.084 | -0.058 | -0.280 | -0.153 | -0.013 | 0.0911 | 0.007 |
|  | P |  |  | 1 | 0.104 | -0.081 | 0.005 | -0.018 | -0.032 | 0.030 | 0.171* | -0.041 | -0.228** | -0.065 | 0.024 | 0.092 | 0.070 |
| **NBP** | G |  |  |  | 1 | -0.071 | -0.371** | -0.158 | 0.039 | 0.109 | -0.254* | 0.068 | 0.009 | 0.013 | -0.108 | 0.242* | 0.186 |
|  | P |  |  |  | 1 | 0.001 | -0.123 | -0.014 | 0.084 | 0.149 | 0.148 | 0.098 | 0.064 | 0.118 | 0.005 | 0.258** | 0.265** |
| **LA** | G |  |  |  |  | 1 | 0.0182 | 0.0151 | 0.049 | -0.195 | 0.124 | 0.146 | 0.003 | 0.019 | 0.237* | -0.083 | -0.090 |
|  | P |  |  |  |  | 1 | 0.111 | 0.061 | 0.067 | -0.151 | 0.233** | 0.165* | 0.039 | 0.137 | 0.271** | -0.052 | -0.030 |
| **LDW** | G |  |  |  |  |  | 1 | 0.097 | -0.180 | -0.199 | -0.645** | -0.092 | -0.184 | -0.108 | 0.028 | -0.212 | -0.111 |
|  | P |  |  |  |  |  | 1 | 0.203* | -0.095 | -0.105 | 0.049 | -0.030 | -0.089 | 0.152 | 0.141 | -0.133 | 0.026 |
| **TDM** | G |  |  |  |  |  |  | 1 | -0.1131 | 0.079 | -0.049 | -0.171 | 0.195 | -0.045 | -0.112 | -0.589** | 0.196 |
|  | P |  |  |  |  |  |  | 1 | -0.058 | 0.115 | 0.215** | -0.132 | 0.253** | 0.034 | 0.0024 | -0.508** | -0.323** |
| **PST** | G |  |  |  |  |  |  |  | 1 | -0.1579 | 0.036 | -0.064 | 0.078 | -0.108 | 0.225 | -0.013 | -0.153 |
|  | P |  |  |  |  |  |  |  | 1 | -0.135 | 0.112 | -0.051 | -0.060 | -0.034 | 0.244** | 0.001 | -0.101 |
| **SPAD** | G |  |  |  |  |  |  |  |  | 1 | -0.509** | 0.039 | 0.281* | 0.097 | -0.048 | -0.017 | 0.066 |
|  | P |  |  |  |  |  |  |  |  |  | -0.156 | 0.054 | 0.287** | 0.142 | -0.003 | 0.008 | 0.109 |
| **CT** | G |  |  |  |  |  |  |  |  |  | 1 | 0.0101 | 0.1008 | -0.3833** | -0.281* | -0.3824** | -0.414** |
|  | P |  |  |  |  |  |  |  |  |  |  | 0.090 | 0.168* | 0.221** | 0.073 | -0.108 | 0.0051 |
| **RWC** | G |  |  |  |  |  |  |  |  |  |  | 1 | 0.0916 | 0.088 | -0.049 | 0.211 | 0.135 |
|  | P |  |  |  |  |  |  |  |  |  |  |  | 0.102 | 0.052 | -0.020 | 0.218** | 0.143 |
| **NCP** | G |  |  |  |  |  |  |  |  |  |  |  | 1 | -0.054 | -0.1991 | -0.291* | -0.18 |
|  | P |  |  |  |  |  |  |  |  |  |  |  |  | -0.001 | -0.139 | -0.274** | -0.074 |
| **TW** | G |  |  |  |  |  |  |  |  |  |  |  |  | 1 | -0.2847* | 0.0206 | 0.0617 |
|  | P |  |  |  |  |  |  |  |  |  |  |  |  |  | -0.075 | 0.093 | 0.105 |
| **CWP** | G |  |  |  |  |  |  |  |  |  |  |  |  |  | 1 | 0.0743 | -0.007 |
|  | P |  |  |  |  |  |  |  |  |  |  |  |  |  |  | 0.099 | 0.814 |
| **HI** | G |  |  |  |  |  |  |  |  |  |  |  |  |  |  | 1 | 0.664** |
|  | P |  |  |  |  |  |  |  |  |  |  |  |  |  |  |  | 0.618** |
| **SWP** | G |  |  |  |  |  |  |  |  |  |  |  |  |  |  |  | 1 |
|  | P |  |  |  |  |  |  |  |  |  |  |  |  |  |  |  | 1 |

**Supplementary Table 13** Direct and indirect effects between seed yield and different morpho-physiological traits among 76 sesame genotypes at genotypic and phenotypic levels under stress(WS) conditions in year 2019.

|  |  | **NDF** | **NDM** | **PH** | **NBP** | **LA** | **LDW** | **TDM** | **PST** | **SPAD** | **CT** | **RWC** | **NCP** | **TW** | **CWP** | **HI** |
| --- | --- | --- | --- | --- | --- | --- | --- | --- | --- | --- | --- | --- | --- | --- | --- | --- |
| **NDF** | G | **-0.07574** | -0.01195 | 0.00749 | 0.00985 | 0.00295 | 0.01308 | -0.03049 | -0.00140 | 0.00482 | -0.00278 | -0.00400 | -0.00250 | 0.00442 | 0.00277 | -0.20229 |
|  | P | -0.04195 | -0.00431 | 0.00053 | -0.00075 | 0.00046 | -0.00031 | 0.07958 | -0.00261 | -0.00041 | -0.01879 | -0.00139 | 0.00014 | -0.00007 | 0.00003 | -0.12764 |
| **NDM** | G | -0.04766 | **-0.01899** | 0.00364 | 0.00884 | -0.00003 | -0.00546 | -0.08763 | -0.00869 | 0.00768 | 0.00816 | -0.00346 | -0.00655 | -0.00180 | 0.00441 | -0.09421 |
|  | P | -0.02767 | -0.00653 | 0.00003 | -0.00056 | -0.00184 | -0.00044 | 0.02778 | -0.0067 | 0.00025 | -0.01346 | -0.00128 | 0.00025 | -0.00007 | 0.0000 | -0.05790 |
| **PH** | G | 0.01318 | 0.00161 | **-0.04303** | -0.00292 | 0.00252 | 0.01234 | -0.07413 | 0.00246 | -0.00073 | -0.0152 | -0.00249 | 0.01477 | 0.00603 | 0.00069 | 0.09087 |
|  | P | 0.00327 | 0.00003 | -0.00683 | 0.00191 | 0.00201 | -0.00001 | -0.01642 | 0.00141 | -0.00067 | -0.00873 | -0.00145 | -0.00032 | 0.00003 | 0.00001 | 0.09646 |
| **NBP** | G | 0.01544 | 0.00347 | -0.00260 | **-0.04832** | 0.00153 | 0.06029 | -0.12375 | -0.00322 | -0.01147 | 0.04572 | 0.00295 | -0.00049 | -0.00054 | 0.00570 | 0.24207 |
|  | P | 0.00173 | 0.00020 | -0.00072 | 0.01829 | -0.00003 | 0.0002 | -0.01240 | -0.00366 | -0.00326 | -0.00759 | 0.00341 | 0.00009 | -0.000050 | 0.000000 | 0.26869 |
| **LA** | G | 0.01046 | -0.00003 | 0.00509 | 0.00347 | **-0.02134** | -0.00295 | 0.01184 | -0.00404 | 0.02056 | -0.02248 | 0.00629 | -0.00017 | -0.00075 | -0.01247 | -0.08359 |
|  | P | 0.00079 | -0.00049 | 0.00056 | 0.00002 | -0.02458 | -0.00025 | 0.05337 | -0.00294 | 0.00329 | -0.01187 | 0.00572 | 0.00005 | -0.00005 | 0.00014 | -0.05467 |
| **LDW** | G | 0.00611 | -0.00064 | 0.00327 | 0.01796 | -0.00039 | **-0.16221** | 0.07620 | 0.01461 | 0.02105 | 0.11615 | -0.00399 | 0.00969 | 0.00428 | -0.00149 | -0.21172 |
|  | P | -0.00584 | -0.00128 | -0.00004 | -0.00225 | -0.00273 | -0.00224 | 0.17776 | 0.00414 | 0.00230 | -0.00250 | -0.00106 | -0.00012 | -0.00006 | 0.00007 | -0.13877 |
| **TDM** | G | 0.00295 | 0.00212 | 0.00407 | 0.00763 | -0.00032 | -0.01578 | **0.78335** | 0.00913 | -0.00842 | 0.00889 | -0.00737 | -0.01027 | 0.00179 | 0.00590 | -0.58742 |
|  | P | 0.00382 | -0.00021 | 0.00013 | -0.00026 | -0.00150 | 0.00046 | 0.87353 | 0.00254 | -0.00253 | -0.01096 | -0.0056 | 0.00035 | -0.00001 | 0.000000 | -0.52855 |
| **PST** | G | -0.00131 | -0.00204 | 0.00227 | -0.00193 | -0.00106 | 0.02935 | -0.08860 | **-0.08075** | 0.01663 | -0.00660 | -0.00737 | -0.01027 | 0.00179 | 0.00590 | -0.58742 |
|  | P | -0.00253 | -0.00092 | 0.00022 | 0.00154 | -0.00167 | 0.00021 | -0.05128 | -0.04325 | 0.00296 | -0.00570 | -0.00179 | -0.00008 | 0.00001 | 0.00012 | 0.00114 |
| **SPAD** | G | 0.00347 | 0.00138 | -0.00030 | -0.00526 | 0.00417 | 0.03242 | 0.06260 | 0.01275 | **-0.10532** | 0.09178 | 0.00169 | -0.01481 | -0.00385 | 0.00253 | -0.01728 |
|  | P | -0.00079 | 0.00008 | -0.00021 | 0.00274 | 0.00371 | 0.00024 | 0.10124 | 0.00587 | -0.02179 | 0.00797 | 0.00189 | 0.00040 | -0.00006 | 0.00000 | 0.00863 |
| **CT** | G | -0.00117 | 0.00086 | -0.00361 | 0.0127 | -0.00266 | 0.10466 | -0.03867 | -0.00296 | 0.05370 | **-0.18002** | 0.00043 | -0.00531 | 0.01508 | 0.01477 | -0.38133 |
|  | P | -0.01551 | -0.00173 | -0.00117 | 0.00273 | -0.00574 | -0.00011 | 0.18842 | -0.00485 | 0.00342 | -0.05080 | 0.00314 | 0.00023 | -0.00009 | 0.00004 | -0.11268 |
| **RWC** | G | 0.00706 | 0.00153 | 0.00249 | -0.00332 | -0.00312 | 0.01505 | -0.13440 | 0.00517 | -0.00415 | -0.00181 | **0.04295** | -0.00482 | -0.00001 | 0.00262 | 0.21036 |
|  | P | 0.00169 | 0.00024 | 0.00029 | 0.00181 | -0.00407 | 0.00007 | -0.11539 | 0.00225 | -0.00119 | -0.00462 | 0.03450 | 0.00014 | -0.00002 | -0.00001 | 0.22733 |
| **NCP** | G | -0.00359 | -0.00236 | 0.01207 | -0.00045 | -0.00007 | 0.02985 | 0.15286 | 0.00637 | -0.02962 | -0.01815 | 0.00393 | **-0.05265** | 0.00213 | 0.01043 | -0.29070 |
|  | P | -0.00407 | -0.00117 | 0.00156 | 0.00117 | -0.00097 | 0.00020 | 0.22144 | 0.00263 | -0.00626 | -0.00855 | 0.00352 | 0.00140 | 0.00000 | -0.00007 | -0.28543 |
| **TW** | G | 0.00852 | -0.00087 | 0.00659 | -0.00066 | -0.00041 | 0.01764 | -0.03556 | 0.00874 | -0.01031 | 0.06899 | 0.00001 | 0.00285 | **-0.03934** | 0.01492 | 0.02055 |
|  | P | -0.00771 | -0.00113 | 0.00044 | 0.00214 | -0.00337 | -0.00034 | 0.02979 | 0.00151 | -0.00310 | -0.01131 | 0.00181 | 0.00000 | -0.00039 | -0.00004 | 0.09729 |
| **CWP** | G | 0.00400 | 0.00160 | 0.00057 | 0.00525 | -0.00508 | -0.00461 | -0.08819 | -0.01817 | 0.00508 | 0.05074 | -0.00214 | 0.01048 | 0.01120 | **-0.05240** | 0.07413 |
|  | P | -0.00279 | -0.00012 | -0.00017 | 0.00011 | -0.00668 | -0.00032 | 0.00210 | -0.01057 | 0.00007 | -0.00370 | -0.00070 | -0.00020 | 0.00003 | 0.00050 | 0.10363 |
| **HI** | G | 0.01537 | 0.00179 | -0.00392 | -0.01173 | 0.00179 | 0.03444 | -0.46149 | 0.00107 | 0.00183 | 0.06884 | 0.00906 | 0.01535 | -0.00081 | -0.00390 | **0.99712** |
|  | P | 0.00515 | 0.00036 | -0.00063 | 0.00473 | 0.00129 | 0.00030 | -0.44419 | -0.00005 | -0.00018 | 0.00551 | 0.00755 | -0.00038 | -0.00004 | 0.00005 | 1.03944 |

***Residual:** 0.0568

**Supplementary Table 14** Principle component analysis showing the eigen values and proportion of variance under WW and WS condition during the year 2018 and 2019.

|  | | **Year-2018** | | | | | | | | | | |
| --- | --- | --- | --- | --- | --- | --- | --- | --- | --- | --- | --- | --- |
|  |  | **Components** | | | | | | | | | | |
|  |  | **Irrigated condition(WW)** | | | | | | | | | | |
|  |  | 1 | | | 2 | | 3 | | 4 | | 5 | |
| Eigen values (R) | | 5.84 | | | 1.93 | | 1.43 | | 1.29 | | 1.05 | |
| Proportion of variance % | | 36.50 | | | 12.11 | | 8.95 | | 8.06 | | 6.60 | |
| Cumulative variance % | | 36.50 | | | 48.62 | | 57.57 | | 65.64 | | 72.24 | |
|  | | **Terminal drought (WS)** | | | | | | | | | | |
| Eigen values (R) | | 7.80 | | | 1.68 | | 1.49 | | 1.10 | | - | |
| Proportion of variance % | | 48.78 | | | 10.52 | | 9.32 | | 6.90 | | - | |
| Cumulative variance % | | 48.78 | | | 59.30 | | 68.63 | | 75.53 | | - | |
|  | **Year-2019** | | | | | | | | | | | |
|  | **Irrigated condition (WW)** | | | | | | | | | | | |
|  | 1 | | 2 | 3 | | 4 | | 5 | | 6 | | 7 |
| Eigen values (R) | 6.49 | | 2.25 | 1.36 | | 1.29 | | 1.00 | | - | | - |
| Proportion of variance % | 40.57 | | 14.06 | 8.53 | | 8.06 | | 6.26 | | - | | - |
| Cumulative variance % | 40.57 | | 54.64 | 63.18 | | 71.24 | | 77.51 | | - | | - |
|  | **Terminal drought (WS)** | | | | | | | | | | | |
| Eigen values (R) | 2.39 | | 1.79 | 1.56 | | 1.35 | | 1.33 | | 1.24 | | 1.20 |
| Proportion of variance % | 14.95 | | 11.20 | 9.77 | | 8.47 | | 8.35 | | 7.76 | | 7.50 |
| Cumulative variance % | 14.95 | | 26.16 | 35.94 | | 44.41 | | 52.77 | | 60.54 | | 68.04 |

**Supplementary Table 15** Principal component analysis for sesame genotypes under WW and WS conditions during year 2018 and 2019; a non-rotated loading

| **Traits** | **Year-2018** | | | | | | | | | | | | | | | | | | | |
| --- | --- | --- | --- | --- | --- | --- | --- | --- | --- | --- | --- | --- | --- | --- | --- | --- | --- | --- | --- | --- |
|  | **Components** | | | | | | | | | | | | | | | | | | | |
|  | **Irrigated condition (WW)** | | | | | | | | | | | **Terminal drought (WS)** | | | | | | | | |
|  | PC1 | | PC2 | | PC3 | | PC4 | | PC5 | | | PC1 | | PC2 | | | PC3 | | PC4 | |
| NDF | 0.031 | | 0.777 | | -0.136 | | 0.015 | | -0.108 | | | -0.170 | | 0.872 | | | 0.000 | | -0.001 | |
| NDM | -0.136 | | 0.607 | | -0.178 | | 0.171 | | 0.312 | | | -0.200 | | 0.849 | | | 0.118 | | 0.059 | |
| PH | 0.317 | | 0.573 | | 0.400 | | 0.061 | | -0.302 | | | 0.398 | | 0.103 | | | -0.627 | | 0.020 | |
| NBP | 0.876 | | 0.063 | | -0.032 | | -0.024 | | 0.092 | | | 0.894 | | 0.118 | | | -0.047 | | 0.044 | |
| LA | 0.862 | | -0.071 | | 0.120 | | -.0173 | | 0.167 | | | 0.950 | | 0.058 | | | 0.020 | | -0.068 | |
| LDW | 0.388 | | 0.090 | | 0.417 | | -0.366 | | 0.636 | | | 0.510 | | -0.192 | | | 0.419 | | -0.101 | |
| TDM | -0.826 | | 0.221 | | 0.248 | | -0.080 | | 0.242 | | | -0.941 | | -0.005 | | | -0.086 | | 0.002 | |
| PST | -0.742 | | 0.043 | | 0.247 | | -0.021 | | 0.295 | | | -0.841 | | -0.100 | | | -0.170 | | -0.131 | |
| SPAD | -0.032 | | -0.364 | | 0.333 | | 0.516 | | 0.030 | | | 0.099 | | 0.012 | | | 0.243 | | 0.855 | |
| CT | 0.153 | | -0.237 | | 0.578 | | 0.508 | | -0.043 | | | -0.404 | | -0.144 | | | 0.540 | | 0.311 | |
| RWC | 0.852 | | 0.032 | | -0.029 | | 0.027 | | 0.223 | | | 0.946 | | -0.076 | | | 0.062 | | -0.010 | |
| SWP | 0.925 | | -0.062 | | 0.056 | | -0.003 | | 0.054 | | | 0.957 | | -0.008 | | | 0.073 | | -0.077 | |
| NCP | 0.461 | | 0.574 | | 0.280 | | 0.167 | | -0.207 | | | 0.964 | | 0.097 | | | 0.028 | | 0.027 | |
| TW | -0.070 | | 0.172 | | -0.178 | | 0.657 | | 0.346 | | | 0.140 | | 0.300 | | | 0.430 | | -0.230 | |
| CWP | -0.294 | | 0.026 | | 0.588 | | -0.317 | | -0.208 | | | -0.385 | | -0.034 | | | 0.568 | | -0.420 | |
| HI | 0.958 | | -0.134 | | -0.071 | | 0.030 | | -0.089 | | | 0.971 | | -0.002 | | | 0.056 | | -0.062 | |
| **Traits** | **Year-2019** | | | | | | | | | | | | | | | | | | | |
|  | **Components** | | | | | | | | | | | | | | | | | | | |
|  | **Irrigated condition (WW)** | | | | | | | | | **Terminal drought (WS)** | | | | | | | | | | |
|  | PC1 | PC2 | | PC3 | | PC4 | | PC5 | | PC1 | PC2 | | PC3 | | PC4 | PC5 | | PC6 | | PC7 |
| NDF | 0.383 | 0.816 | | 0.240 | | -0.171 | | -0.182 | | 0.616 | -0.062 | | 0.533 | | 0.006 | 0.288 | | 0.099 | | 0.141 |
| NDM | 0.431 | 0.758 | | 0.211 | | -0.266 | | -0.221 | | 0.544 | -0.134 | | 0.562 | | 0.084 | 0.382 | | 0.016 | | 0.128 |
| PH | 0.141 | 0.192 | | -0.414 | | -0.504 | | 0.461 | | -0.222 | -0.177 | | -0.128 | | -0.348 | 0.094 | | 0.551 | | 0.005 |
| NBP | 0.872 | 0.046 | | -0.211 | | 0.009 | | 0.028 | | -0.397 | 0.151 | | 0.291 | | 0.025 | -0.290 | | 0.280 | | 0.256 |
| LA | 0.925 | 0.015 | | 0.000 | | 0.062 | | 0.179 | | 0.126 | -0.367 | | -0.096 | | 0.571 | -0.292 | | -0.203 | | -0.211 |
| LDW | 0.880 | -0.001 | | 0.051 | | 0.060 | | 0.191 | | 0.148 | -0.150 | | -0.385 | | 0.269 | 0.597 | | -0.077 | | -0.165 |
| TDM | -0.836 | 0.325 | | -0.045 | | 0.079 | | -0.031 | | 0.364 | 0.454 | | -0.435 | | 0.446 | 0.011 | | 0.374 | | 0.243 |
| PST | -0.778 | 0.229 | | -0.244 | | 0.206 | | -0.066 | | 0.099 | -0.435 | | 0.151 | | 0.004 | -0.315 | | -0.082 | | 0.537 |
| SPAD | -0.024 | 0.091 | | 0.364 | | 0.750 | | 0.103 | | -0.133 | 0.655 | | 0.121 | | -0.089 | 0.040 | | -0.302 | | 0.265 |
| CT | 0.009 | -0.062 | | 0.730 | | -0.190 | | 0.517 | | 0.412 | -0.242 | | 0.149 | | 0.143 | -0.402 | | 0.560 | | -0.189 |
| RWC | 0.175 | -0.285 | | 0.379 | | -0.292 | | -0.550 | | -0.257 | -0.026 | | 0.259 | | 0.274 | -0.278 | | -0.180 | | -0.418 |
| SWP | 0.802 | -0.026 | | -0.220 | | 0.290 | | -0.110 | | -0.567 | 0.244 | | 0.086 | | 0.574 | 0.214 | | 0.342 | | 0.198 |
| NCP | 0.820 | 0.136 | | -0.195 | | 0.077 | | -0.123 | | 0.373 | 0.557 | | 0.123 | | 0.119 | -0.359 | | -0.192 | | 0.073 |
| TW | -0.465 | 0.437 | | -0.275 | | 0.052 | | 0.037 | | 0.051 | 0.226 | | 0.383 | | 0.262 | 0.136 | | 0.012 | | -0.302 |
| CWP | 0.078 | 0.693 | | 0.076 | | 0.288 | | 0.150 | | -0.069 | -0.470 | | -0.171 | | 0.312 | 0.017 | | -0.257 | | 0.513 |
| HI | 0.935 | -0.169 | | -0.080 | | 0.145 | | -0.052 | | -0.781 | -0.185 | | 0.437 | | 0.131 | 0.197 | | -0.005 | | 0.004 |

**Supplementary Table 16** Constituents of 10 clusters of 76 sesame accessions during year 2018 under WW and WS conditions

| **Clusters** | **Irrigated (WW)** | | **Terminal drought (WS)** | |
| --- | --- | --- | --- | --- |
|  | **Number of genotypes** | **Genotypes** | **Number of genotypes** | **Genotypes** |
| Cluster 1 | 11 | GT-10, IC 110221, IC 132171, IC 132383, IC 17476-1, IC 204861, IC 205206, IC 205311, IC 205791, IC 23279, IC 96116 | 10 | GT-10, IC 204300, IC 204572, IC 204706, IC 204747, IC 204842, IC 205304, IC 205476, IC 23279, IC 96126, |
| Cluster 2 | 8 | IC 131497, IC 131943, IC 132167, IC 132207, IC 132389, IC 205285, IC 41920, IC 96229 | 14 | IC 110221, IC 132186, IC 132383, IC 132386, IC 132387, IC 203962, IC 204677, IC 205791, IC 54035, IC 74188, IC 96127, IC 96231, IC 96240, IC 131497, |
| Cluster 3 | 14 | IC 131500, IC 131878, IC 204045, IC 204046, IC 204300, IC 204406, IC 204611, IC 204677, IC 205476, IC 205724, IC 42999, IC 74188, IC 96127, IC 96233 | 15 | IC 131500, IC 131559, IC 132293, IC 132300, IC 132389, IC 132410, IC 132558, IC 17476-1, IC 204445, IC 204611, IC 204679, IC 23297, IC 42999, IC 43033, IC 81564 |
| Cluster 4 | 19 | IC 131546, IC 131953, IC 132186, IC 132293, IC 132300, IC 132387, IC 132558, IC 203962, IC 204572, IC 204666, IC 204706, IC 204747, IC 204753, IC 204789, IC 204842, IC 204849, IC 23297, IC 96231, IC 96240 | 8 | IC 131546, IC 131936, IC 132171, IC 16244, IC 204280, IC 205209, IC 205285, IC 73576, |
| Cluster 5 | 2 | IC 131559, IC 205304 | 12 | IC 131878, IC 131943, IC 131953,IC 132167, IC 132176, IC 204406, IC 205471, IC 43036, IC 96229, IC 96232, IC 96233, TKG-22 |
| Cluster 6 | 5 | IC 131936, IC 16244, IC 205363, IC 43036, IC 81564 | 5 | IC 132207, IC 204849, IC 205479, IC 204045, IC 204046, |
| Cluster 7 | 12 | IC 132176, IC 132410, IC 204445, IC 204679, IC 205209, IC 205471, IC 205479, IC 43033, IC 54035, IC 96126, IC 96232, TKG-22 | 2 | IC 205353, IC 205363 |
| Cluster 8 | 1 | IC 132386, | 5 | IC 204545, IC 204753, IC 204861, IC 205724, IC 41920, |
| Cluster 9 | 3 | IC 204280, IC 204545, IC 205353 | 2 | IC 204666, IC 96116 |
| Cluster 10 | 1 | IC 73576 | 3 | IC 204789, IC 205206, IC 205311 |

**Supplementary Table 17** Constituents of 10 clusters of 76 accessions of sesame during year 2019 under WW and WS conditions

| **Clusters** | **Irrigated (WW)** | | **Terminal drought (WS)** | |
| --- | --- | --- | --- | --- |
|  | **Number of genotypes** | **Genotypes** | **Number of genotypes** | **Genotypes** |
| Cluster 1 | 10 | GT-10, IC 131497, IC 131500, IC 132410, IC 16244, IC 204545, IC 204677, IC 204706, IC 23297, IC 73576, | 19 | GT-10, IC 131878, IC 131953, IC 132186, IC 132207, IC 132410, IC 16244, IC 17476-1, IC 204280, IC 204300, IC 204545, IC 204666, IC 204747, IC 204861, IC 205304, IC 41920, IC 54035, IC 96231, TKG-22 |
| Cluster 2 | 11 | IC 110221, IC 203962, IC 204280, IC 204445, IC 204572, IC 205209, IC 205285, IC 205479, IC 23279, IC 96116, IC 96231 | 9 | IC 110221, IC 132167, IC 204753, IC 205285, IC 205791, IC 23297, IC 74188, IC 96229, IC 96233 |
| Cluster 3 | 11 | IC 132167, IC 132176, IC 132207, IC 132383, IC 17476-1, IC 204046, IC 204849, IC 205206, IC 54035, IC 81564, IC 96127, | 10 | IC 131497, IC 132176, IC 204406, IC 204611, IC 204789, IC 204849, IC 205724, IC 43036, IC 73576, IC 96240 |
| Cluster 4 | 15 | IC 131546, IC 131943, IC 131953, IC 132386, IC 132387, IC 132558, IC 204666, IC 204842, IC 205304, IC 205363, IC 205471, IC 205724, IC 42999, IC 43036, IC 96229, | 9 | IC 131500, IC 131546, IC 132300, IC 132383, IC 132386, IC 132387, IC 132558, IC 205311, IC 43033 |
| Cluster 5 | 4 | IC 131559, IC 132171, IC 132389, IC 74188, | 6 | IC 131559, IC 131943, IC 132293, IC 132389, IC 204842, IC 96232 |
| Cluster 6 | 7 | IC 131878, IC 204045, IC 204679, IC 204789, IC 204861, IC 96232, IC 96240 | 5 | IC 131936, IC 204572, IC 205209, IC 205353, IC 96127 |
| Cluster 7 | 4 | IC 131936, IC 132186, IC 205476, IC 96126, | 6 | IC 132171, IC 204045, IC 204679, IC 205471, IC 42999, IC 96126 |
| Cluster 8 | 4 | IC 132293, IC 204300, IC 204611, IC 41920, | 6 | IC 203962, IC 204445, IC 204677, IC 205206, IC 205363, IC 23279 |
| Cluster 9 | 9 | IC 132300, IC 204406, IC 204747, IC 205311, IC 205353, IC 205791, IC 43033, IC 96233, TKG-22 | 1 | IC 204046 |
| Cluster 10 | 1 | IC 204753 | 4 | IC 204706, IC 205476, IC 205479, IC 96116 |

**Supplementary Table 18** Cluster means of 10 clusters of 76 sesame genotypes during year 2018 under WW and WS conditions

|  | **Irrigated (WW)** | | | | | | | | | |
| --- | --- | --- | --- | --- | --- | --- | --- | --- | --- | --- |
| **Traits** | **Cluster 1** | **Cluster 2** | **Cluster 3** | **Cluster 4** | **Cluster 5** | **Cluster 6** | **Cluster 7** | **Cluster 8** | **Cluster 9** | **Cluster 10** |
| NDF | 41.71 | 41.19 | 41.38 | 41.02 | 41.33 | 40.49 | 39.00 | 40.79 | 41.79 | 40.24 |
| NDM | 97.05 | 95.31 | 94.44 | 94.11 | 97.33 | 100.66 | 95.00 | 93.26 | 100.06 | 97.19 |
| PH | 74.60 | 74.32 | 69.01 | 75.90 | 86.30 | 67.30 | 67.57 | 80.14 | 78.46 | 73.35 |
| NBP | 5.65 | 4.93 | 4.03 | 5.42 | 6.50 | 3.70 | 6.20 | 6.22 | 4.52 | 5.23 |
| LA | 773.84 | 603.0 | 429.60 | 856.62 | 1248.31 | 314.34 | 1126.35 | 972.14 | 491.56 | 692.08 |
| LDW | 7.87 | 6.95 | 5.49 | 8.35 | 9.70 | 5.73 | 7.20 | 7.36 | 6.83 | 8.11 |
| TDM | 20.18 | 21.62 | 24.88 | 20.81 | 16.50 | 25.41 | 14.63 | 15.50 | 23.52 | 20.30 |
| PST | 9.74 | 9.86 | 12.76 | 10.41 | 7.65 | 12.61 | 9.00 | 7.44 | 11.57 | 9.64 |
| SPAD | 47.73 | 48.65 | 46.07 | 48.02 | 40.60 | 55.05 | 45.25 | 51.75 | 47.49 | 47.50 |
| CT | 29.90 | 30.16 | 30.30 | 30.12 | 31.06 | 29.36 | 31.95 | 31.06 | 30.98 | 29.76 |
| RWC | 80.77 | 75.14 | 64.18 | 82.30 | 86.43 | 68.28 | 88.87 | 86.36 | 74.02 | 80.50 |
| SWP | 7.58 | 6.79 | 4.99 | 8.33 | 11.65 | 4.61 | 11.00 | 10.39 | 5.97 | 7.47 |
| NCP | 87.92 | 93.63 | 65.83 | 97.51 | 122.50 | 73.28 | 106.63 | 110.29 | 107.47 | 81.10 |
| TW | 3.60 | 3.75 | 3.48 | 3.54 | 4.18 | 4.08 | 3.91 | 3.28 | 4.12 | 3.48 |
| CWP | 7.85 | 8.71 | 8.53 | 8.57 | 7.46 | 9.30 | 8.51 | 7.75 | 8.22 | 8.60 |
| HI | 27.27 | 24.00 | 16.79 | 28.91 | 41.38 | 15.37 | 42.91 | 40.10 | 20.31 | 26.97 |
| **Terminal drought (WS)** | | | | | | | | | | |
| NDF | 40.27 | 42.33 | 39.76 | 41.99 | 40.33 | 41.66 | 41.36 | 39.42 | 40.22 | 41.49 |
| NDM | 93.25 | 92.75 | 93.43 | 91.11 | 94.00 | 95.36 | 93.77 | 90.33 | 92.55 | 94.79 |
| PH | 69.28 | 60.34 | 54.33 | 66.66 | 64.10 | 56.20 | 61.92 | 62.55 | 62.40 | 61.84 |
| NBP | 5.24 | 3.54 | 3.85 | 6.00 | 6.10 | 3.39 | 4.18 | 5.10 | 2.96 | 4.64 |
| LA | 655.65 | 360.07 | 403.38 | 739.06 | 795.48 | 319.57 | 458.29 | 578.44 | 268.30 | 516.53 |
| LDW | 4.93 | 4.38 | 4.95 | 6.33 | 5.96 | 4.05 | 4.99 | 5.35 | 3.62 | 4.88 |
| TDM | 13.11 | 18.02 | 17.28 | 10.68 | 9.44 | 20.15 | 16.01 | 14.39 | 24.86 | 15.14 |
| PST | 4.34 | 8.33 | 7.86 | 2.59 | 5.39 | 9.37 | 6.67 | 5.50 | 9.83 | 5.53 |
| SPAD | 55.51 | 54.94 | 54.35 | 53.89 | 53.31 | 54.23 | 53.42 | 54.70 | 55.60 | 54.72 |
| CT | 32.62 | 33.18 | 33.23 | 31.67 | 30.60 | 33.15 | 33.85 | 32.88 | 32.82 | 32.64 |
| RWC | 79.21 | 57.85 | 62.39 | 82.62 | 83.47 | 58.15 | 65.83 | 76.02 | 48.04 | 68.22 |
| SWP | 6.21 | 3.70 | 4.26 | 7.59 | 7.99 | 3.48 | 4.40 | 5.59 | 2.50 | 5.16 |
| NCP | 83.60 | 61.05 | 61.47 | 92.69 | 98.63 | 56.13 | 66.67 | 75.36 | 47.94 | 71.17 |
| TW | 3.12 | 3.22 | 3.13 | 3.57 | 3.31 | 3.29 | 3.12 | 3.25 | 2.54 | 3.18 |
| CWP | 4.28 | 4.92 | 5.29 | 3.27 | 3.58 | 4.94 | 4.70 | 4.62 | 5.64 | 4.92 |
| HI | 32.09 | 17.20 | 19.93 | 41.53 | 45.85 | 14.96 | 21.61 | 28.04 | 9.17 | 25.42 |

**Supplementary Table 19** Cluster means of 10 clusters of 76 sesame genotypes during year 2019 under WW and WS conditions

|  | **Irrigated (WW)** | | | | | | | | | |
| --- | --- | --- | --- | --- | --- | --- | --- | --- | --- | --- |
| **Traits** | **Cluster 1** | **Cluster 2** | **Cluster 3** | **Cluster 4** | **Cluster 5** | **Cluster 6** | **Cluster 7** | **Cluster 8** | **Cluster 9** | **Cluster 10** |
| NDF | 43.12 | 44.00 | 38.59 | 42.44 | 41.02 | 39.61 | 41.91 | 42.24 | 40.34 | 40.08 |
| NDM | 102.86 | 104.99 | 95.33 | 101.33 | 98.87 | 97.29 | 100.66 | 101.83 | 98.07 | 97.05 |
| PH | 75.19 | 73.10 | 72.98 | 78.71 | 70.79 | 72.15 | 70.57 | 80.22 | 74.90 | 73.55 |
| NBP | 5.94 | 6.40 | 3.56 | 6.16 | 3.92 | 5.61 | 6.20 | 5.75 | 5.23 | 4.85 |
| LA | 848.55 | 1176.59 | 397.55 | 1063.18 | 488.25 | 790.91 | 1014.52 | 926.82 | 679.26 | 583.05 |
| LDW | 8.85 | 10.59 | 4.68 | 10.70 | 4.72 | 7.39 | 10.68 | 9.19 | 6.95 | 5.68 |
| TDM | 20.78 | 15.39 | 29.37 | 17.20 | 27.36 | 20.49 | 19.53 | 21.30 | 22.50 | 24.43 |
| PST | 11.14 | 8.91 | 14.91 | 9.38 | 13.56 | 10.88 | 9.64 | 10.90 | 11.31 | 11.94 |
| SPAD | 46.11 | 55.65 | 46.81 | 50.25 | 47.60 | 47.56 | 47.94 | 45.84 | 48.17 | 49.35 |
| CT | 29.03 | 29.27 | 29.05 | 29.72 | 29.50 | 29.34 | 30.06 | 29.04 | 29.55 | 29.11 |
| RWC | 72.42 | 85.60 | 80.01 | 72.16 | 70.41 | 74.05 | 78.71 | 73.32 | 73.50 | 73.26 |
| SWP | 8.27 | 12.72 | 5.57 | 10.29 | 7.03 | 8.37 | 10.02 | 8.69 | 6.82 | 7.17 |
| NCP | 117.80 | 149.22 | 65.78 | 134.36 | 73.05 | 103.38 | 117.32 | 91.15 | 85.56 | 84.01 |
| TW | 3.82 | 3.31 | 3.57 | 3.20 | 3.93 | 3.32 | 3.34 | 3.21 | 3.55 | 3.78 |
| CWP | 10.76 | 11.96 | 10.32 | 11.45 | 9.83 | 10.20 | 10.11 | 10.27 | 9.38 | 10.09 |
| HI | 28.43 | 45.25 | 15.94 | 37.53 | 20.54 | 29.03 | 34.21 | 29.63 | 23.53 | 22.78 |
| **Terminal drought (WS)** | | | | | | | | | | |
| NDF | 39.60 | 41.58 | 40.63 | 42.14 | 41.22 | 40.79 | 41.40 | 35.99 | 40.10 | 40.73 |
| NDM | 93.72 | 93.03 | 94.21 | 95.11 | 92.44 | 93.86 | 94.58 | 89.66 | 91.50 | 94.81 |
| PH | 59.18 | 61.32 | 63.32 | 58.19 | 62.96 | 65.16 | 63.51 | 60.30 | 62.54 | 65.25 |
| NBP | 4.43 | 4.02 | 4.60 | 3.82 | 4.20 | 4.09 | 4.40 | 3.80 | 4.36 | 4.04 |
| LA | 644.54 | 419.12 | 368.29 | 502.34 | 689.33 | 234.20 | 460.56 | 770.47 | 305.25 | 574.09 |
| LDW | 4.63 | 5.03 | 4.94 | 4.66 | 5.33 | 4.60 | 4.30 | 5.04 | 4.14 | 4.64 |
| TDM | 17.64 | 15.88 | 17.87 | 14.91 | 18.23 | 16.11 | 14.71 | 14.56 | 18.35 | 18.16 |
| PST | 8.77 | 7.69 | 7.98 | 6.93 | 4.35 | 6.03 | 7.79 | 11.97 | 6.12 | 7.35 |
| SPAD | 50.47 | 54.30 | 54.47 | 51.60 | 56.80 | 57.15 | 53.45 | 53.85 | 57.63 | 55.09 |
| CT | 33.52 | 32.73 | 33.74 | 33.78 | 33.05 | 32.08 | 33.73 | 32.50 | 32.85 | 33.80 |
| RWC | 66.32 | 59.73 | 67.31 | 68.39 | 73.08 | 64.28 | 62.28 | 71.66 | 62.11 | 65.26 |
| SWP | 4.98 | 4.62 | 4.99 | 4.64 | 5.14 | 5.84 | 4.06 | 4.07 | 5.15 | 5.49 |
| NCP | 75.18 | 63.33 | 71.56 | 73.68 | 66.31 | 67.60 | 70.49 | 58.40 | 78.90 | 71.69 |
| TW | 3.12 | 3.25 | 2.92 | 3.29 | 3.39 | 3.16 | 3.09 | 2.59 | 3.05 | 3.32 |
| CWP | 6.44 | 5.65 | 5.48 | 6.21 | 4.90 | 4.89 | 5.62 | 8.79 | 5.90 | 6.57 |
| HI | 23.16 | 23.79 | 21.82 | 23.47 | 21.58 | 26.84 | 21.94 | 21.87 | 22.23 | 23.70 |

**Supplementary Table 20** Mean and range values of genetic diversity measures in the sesame genotypes based on 48 polymorphic simple sequence repeat (SSR) loci

| **SSR locus** | **N_A_** | **H_e_** | **PIC** |
| --- | --- | --- | --- |
| SIM4 | 2 | 0.38 | 0.3 |
| SIM5 | 2 | 0.36 | 0.3 |
| SIM8 | 3 | 0.04 | 0.04 |
| SIM12 | 2 | 0.08 | 0.07 |
| SIM14 | 4 | 0.29 | 0.27 |
| SIM16 | 2 | 0.12 | 0.12 |
| SIM17 | 2 | 0.08 | 0.07 |
| SIM19 | 3 | 0.51 | 0.46 |
| SIM25 | 2 | 0.39 | 0.31 |
| SIM28 | 2 | 0.22 | 0.2 |
| SIM34 | 2 | 0.45 | 0.34 |
| SIM37 | 3 | 0.38 | 0.34 |
| SIM38 | 2 | 0.45 | 0.35 |
| SIM39 | 3 | 0.29 | 0.27 |
| SIM52 | 2 | 0.25 | 0.22 |
| SIM53 | 3 | 0.51 | 0.43 |
| SIM57 | 2 | 0.34 | 0.29 |
| SIM61 | 2 | 0.05 | 0.05 |
| SIM63 | 2 | 0.15 | 0.13 |
| SIM67 | 2 | 0.21 | 0.19 |
| SIM74 | 2 | 0.39 | 0.31 |
| SIM75 | 2 | 0.28 | 0.24 |
| SIM76 | 2 | 0.18 | 0.17 |
| SIM78 | 2 | 0.05 | 0.05 |
| SIM79 | 2 | 0.12 | 0.12 |
| SIM86 | 2 | 0.39 | 0.31 |
| SIM91 | 2 | 0.24 | 0.21 |
| SIM94 | 2 | 0.09 | 0.09 |
| SIM96 | 2 | 0.01 | 0.12 |
| SIM98 | 2 | 0.47 | 0.36 |
| SIM107 | 4 | 0.15 | 0.14 |
| SIM109 | 2 | 0.28 | 0.24 |
| SIM116 | 3 | 0.39 | 0.32 |
| SIM117 | 2 | 0.15 | 0.13 |
| SIM119 | 3 | 0.2 | 0.19 |
| SIM120 | 2 | 0.03 | 0.03 |
| SIM123 | 2 | 0.1 | 0.09 |
| SIM124 | 2 | 0.36 | 0.3 |
| SIM126 | 2 | 0.26 | 0.22 |
| SIM127 | 3 | 0.13 | 0.12 |
| SIM139 | 2 | 0.24 | 0.21 |
| SIM141 | 3 | 0.45 | 0.35 |
| SIM145 | 4 | 0.29 | 0.28 |
| SIM152 | 2 | 0.27 | 0.28 |
| SIM160 | 2 | 0.1 | 0.1 |
| SIM197 | 2 | 0.02 | 0.11 |
| SIM201 | 2 | 0.39 | 0.31 |
| SIM216 | 2 | 0.3 | 0.26 |
| **Mean** | **2.31** | **0.25** | **0.22** |
| **Range** | **2-4** | **0.04-0.51** | **0.03-0.46** |

**Supplementary Table 21** SSR locus used for genetic diversity studies in sesame

| **Primer** | **Forward primer** | **Reverse primer** |
| --- | --- | --- |
| SIM4 | TTTCCATAAGGGGGTATTTGC | GGGATCGATGGTCTAAAAATTC |
| SIM5 | TGGAATGGTCAAATGGGTTT | CTGCCTCATCGTTACACCCT |
| SIM6 | ACATGTGCTAACGTGGACGA | TAGAAAAACGCAAGCAACCC |
| SIM7 | AGATGCATTCCCTGGACATT | TTTGGTATGCAAGGATGCAA |
| SIM8 | TCGAGCTGAAGATCGACAAG | TTGCTTTTTGGTTGTTGTGG |
| SIM9 | GGACTCATTCAGTGGACCGT | TTCTTCCCACCTTCAACACA |
| SIM12 | TCCCATCATTCAATTGGACA | GCATGCAAAGACGTGACAAC |
| SIM14 | CAACGTAAATTTTGTATTTTACCAAT | CAAACGACCCCATACTCTTTG |
| SIM16 | TTTTTGGGAAGAAATTGCATAAA | CCCTTTGGGATGGCTACATA |
| SIM17 | ATGAAACCTAACCAGGGGGA | GAGCTGTAGGTGGTTCAGGC |
| SIM19 | GACCCACATCGAATGTGAAA | TGAACTCTTCAACTATACAATGGGA |
| SIM22 | CACCACCACTAAGACAGCGA | TGTGCAGATCATGATAGGGG |
| SIM25 | TATGTCGGCCGTCGTATCTT | ACATTCTTCCCTAGCTCGCA |
| SIM25 | TATGTCGGCCGTCGTATCTT | ACATTCTTCCCTAGCTCGCA |
| SIM28 | GGCACCAAGTTTTGATGGTT | TTTTGAGATGGTTGAATGGG |
| SIM33 | GGGCCTATCCCAAGTATTGA | GCTTGCGTCCCAAAAATAAA |
| SIM34 | TAAGGAGGCCACTTGCTCAT | AGTGTTGGGTGAGGGAAAAA |
| SIM38 | TATGAACCCAACTTGCACCA | CATTTTGTCTCCTTTCTTTTTAAGG |
| SIM52 | CTCGATTCTGGACGGATGAT | AGCGGGTGTTTGATTTGAAG |
| SIM53 | TCGACGCTATTCTTTCACAAG | AAAAGGGGTTGAGTGTCGAA |
| SIM61 | TACTGGAACTTGGTACGCCC | GCATAACCAAACATCTAGTGGC |
| SIM63 | CTTGTTGTGGTCTTGGACCC | GGACAGCAAGAGCATGATGA |
| SIM67 | GCACTCCACTTTGGGTAAGC | TACCAAGGATCGGCCTTATG |
| SIM74 | GCACCCTTAACTTGGCATTC | TTGATGCAGTTATGTGCTCACT |
| SIM78 | CCAGACCCAAACCCAATAGA | TGCATTTAAGGCTGTGCAAC |
| SIM79 | AATTTATCCGCCTGCACAAC | CGACATTGCATAGATGAGCG |
| SIM86 | TCAGTACCTTAAATCAAGCCGA | GTGGGCGCAGTTAGCTGTA |
| SIM91 | CCCCTGACCTTTCAGTTACG | ATCCTATGCACCACACAGCA |
| SIM98 | CTTTGATTGGGCCACCCTA | TGTTTGTTCTTCTTCCCCCA |
| SIM107 | CCTTGCCAATGAAACTTTCTC | AATCATTTCCAGTGGGGACA |
| SIM109 | CTGCCATTGCATCATCAACT | GGCGAGGTAGGCACAGATTA |
| SIM116 | AATTGTCCTTCTCGGTGGTG | CTGCATCAGGATCTCCGAA |
| SIM117 | AAGGGTGGGAGGAGAGAGAA | AACGGTTTGGACAAAGATCG |
| SIM120 | GCGTTGTAATAACAGGGCGA | TGATTGGTTTGATTCGGTCA |
| SIM123 | AAGCAATGCTGCTGCTACAA | CCCCTACCCCAAACCTAAAC |
| SIM124 | TGGCACCATGATAGGACAAA | AAACTTGATCACCCACAAAAC |
| SIM126 | TGTCCGTGTTTCAGCTTTTCT | CAAATTGCGGGCTCATCTAT |
| SIM127 | AAGGAAGCGAAGAGAATGGG | GCGTGGTTTCTTGAACGATAA |
| SIM137 | TTATGGGGAGAACTAGGGGC | TGGGGTTTCCATTATTGCTC |
| SIM139 | GAGAGAGATTTTGAGGCGGA | TGTTGATGGTGTCTGGTATTGA |
| SIM140 | AGTTATTTTAAAGGGTCTTCTCGTT | AAGAGTCTGCCCATGACACC |
| SIM141 | CACGTAATTCATCGTGATCCA | AGAAGTTTGAGTTTGGCGGA |
| SIM142 | TGTCCACATCAAATGTCCCT | TGTGGAACTCGAATTTGTGAA |
| SIM143 | GCAGAAATAGAACTTGGAGGGA | TCTGCTAGAACATCGAAACCAA |
| SIM144 | AATTACAAGGCGGGTAGCCT | CAACTTCTTCATGTTCGTCCTT |
| SIM145 | ATCCCCTGAGGAGTGTCGAT | GCACAAACATCCCTGACCTT |
| SIM146 | GCACCACAATTGTCGAAGG | TTCATCTCACAAGTTCCACCA |
| SIM147 | AAAGCACGAATTGATGCCC | GCAATTAATCGTTTAGTTCCATATT |
| SIM152 | GAGCCAAATCTTAAGGGTTTCA | TTGTGAGGATCGGAAAGACC |
| SIM154 | CGTATGACGTTAACTTCGCAGA | AAAAAGGAGGTGGCGATTAGA |
| SIM155 | TTTTCTCCTATTGCGCTCGT | GGACCCCAACAACAGCTAAA |
| SIM156 | TTAGGGCCTAGGGTACGGAT | TGCATCACTTTGTGGAGTGG |
| SIM157 | CACCACCCCATTGGACTAAC | GAGAAGGGGATTTTGCTGTG |
| SIM160 | CCATACTGCTGCTGTTGCTG | TCCATCAAGAGTTTCTCGGG |
| SIM161 | GTGGCGGAGTACAGTTTGGT | TCATTTTCAATGTCACAGGAGG |
| SIM163 | GTAACCCTATTCCCCCGTGT | GCCAGAAAACAATCAAGGGA |
| SIM164 | TCAAACAAAAGTGACAGCCAA | CATGCATGAGTCGTAACCAGA |
| SIM166 | GGACGCATACACAACCACAT | TTTCGATAACCGAGGAATGG |
| SIM167 | CCTCCCTAGATTTTCGATTGC | GGCACATGTTCACAAATCCA |
| SIM193 | GCCAAAACAAAGGATTCAAGA | TGAGCTTTGTGTGACCATGA |
| SIM195 | TGGTCCGATTAACTCATGTTG | CTGCAACCCAAACCTAGGAG |
| SIM196 | TCACCAACTTCTCATTCCAAAA | GGGTTGTTTGCATTTGTTCC |
| SIM197 | CAATGGATAAGGAGGCGAAA | ATCGGTCATTCCAATCCTCA |
| SIM198 | CATAAAACCAGCAGAGCAGC | TAAACCTCGACCTGACGCTT |
| SIM199 | TCCATCGATGCTGGACTGTA | CAAGTTAAATATATGCTGTACATGTGG |
| SIM201 | TCATTAACCCATCATTGCGA | TGCTCACACATAACAGTTGGG |
| SIM202 | ACTTGCAATTTTAGTCCGGC | CCAAACCAAAACGTACCCAC |
| SIM204 | TTGACTTGCCTCATGCATTT | TGCAGTTCAATTGTCTGGGA |
| SIM205 | ATTATGCGCGTCAGTGTGTG | CGCTATAATATTTGGGGGCA |
| SIM207 | TGTTGTTTGACCGTCTTCCA | TCGGGCTAGAAACCAACAGT |
| SIM208 | TTTAATAATTCATTTGATGGATGG | ACTTCAAGTATGCGTTGGGC |
| SIM213 | CCAACAAGATGGAATGTCCC | AGGACAAGTCAATGGATGAAA |
| SIM215 | GCCAGAGGTGGTCACAATTT | GCTTCTCAACTATTCCTCCCTG |
| SIM216 | CTTGACCTAATTGGGAGGGG | TGGGAGTATTGGGGTCTTCA |
| SIM218 | ATAATGCGACCCATCCTCAA | CCCACCGTCTCTAACCAAGA |
