# Supplementary material for: Identifying Traits Associated With Terminal Drought Tolerance in Sesame (Sesamum indicum L.) Genotypes
Source: Front Plant Sci. 2021 Dec 10;12:739896. doi: 10.3389/fpls.2021.739896 (PMC8709571; doi:10.3389/fpls.2021.739896)
Supplement: Supplementary file 2 [file Data_Sheet_2.docx]

**
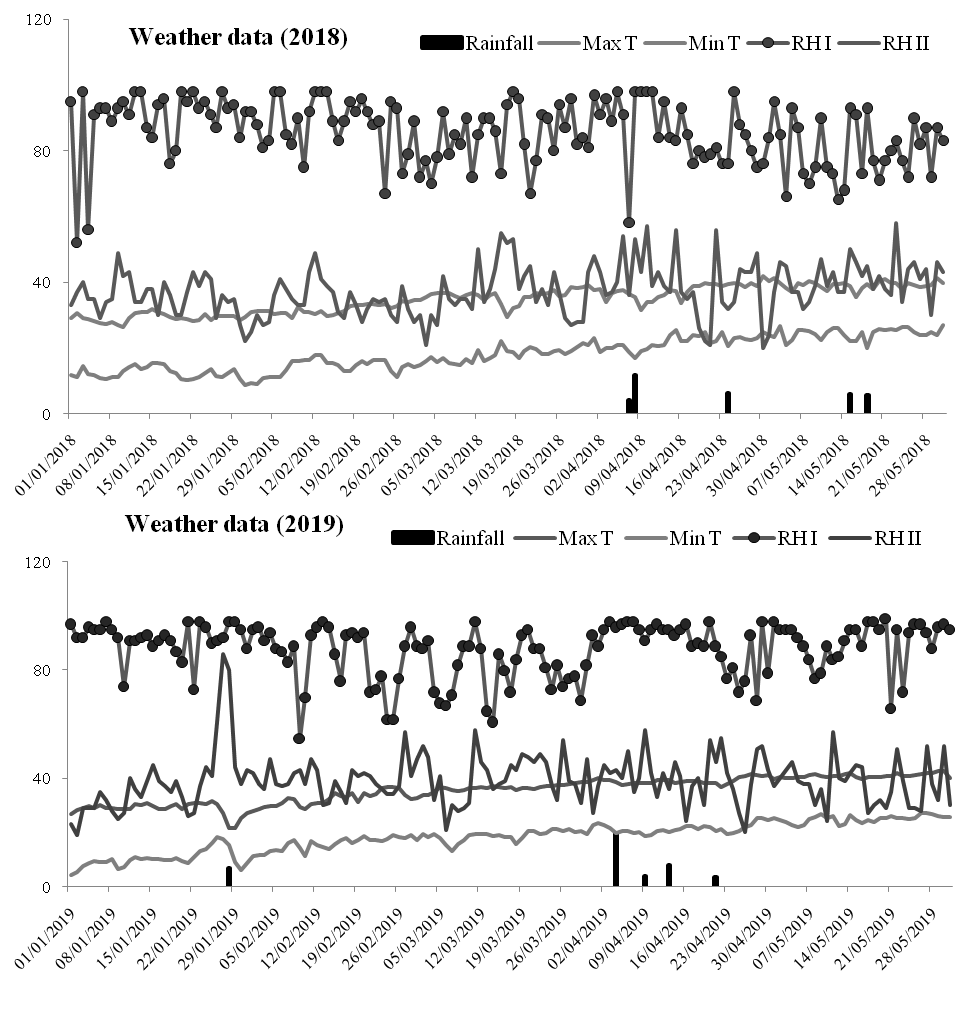
**

**Supplementary Figure 1.** Weather data *i.e.*, rainfall (mm), maximum relative humidity I and minimum II (%); minimum and maximum temperature (^o^C) during late *rabi* season in year 2018 and 2019.


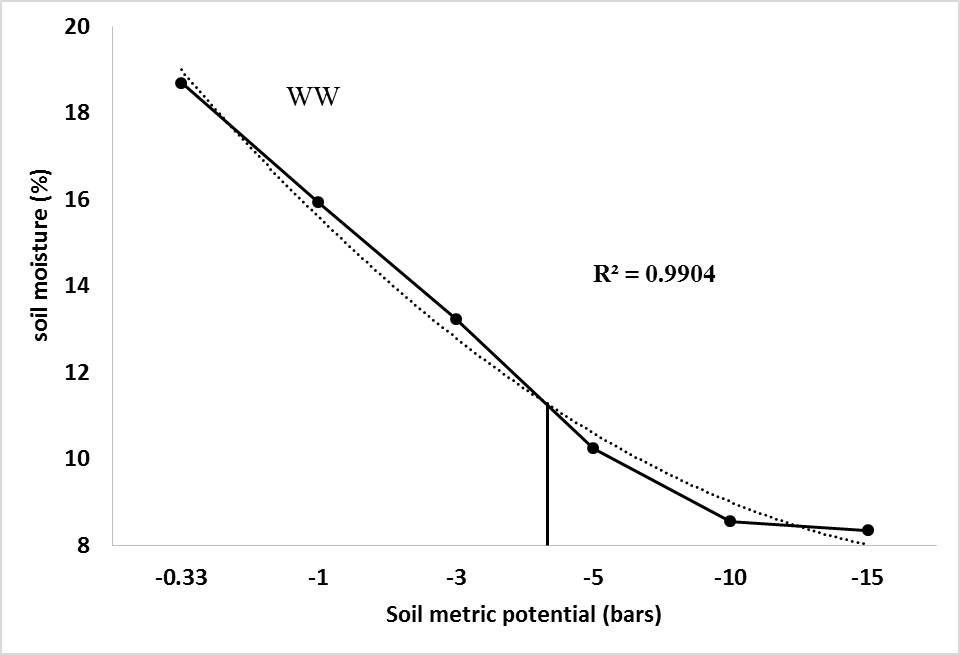


WS

**Supplementary Figure 2:** Soil moisture (%) average values under WW and WS conditions, fitted in to the soil surface tensiometer curve to draw the accurate soil metric potential (bars) of both sesame growing seasons. The dotted line (- - -) represent *rabi* season 2018 and unbroken (__) line represent the *rabi* 2019 of both water treatments.
